# IL1β Functions as a Tumor Suppressor in Papillary Thyroid Carcinoma via the SMDT1-MAPK Signaling Axis

**DOI:** 10.3390/cancers18182918

**Published:** 2026-09-09

**Authors:** Tenghong Liu, Liyong Zhang, Zhijun Chen, Shaojun Cai, Wenxin Zhao

**Affiliations:** Department of Thyroid Surgery, Fujian Medical University Union Hospital, Fuzhou 350001, China; liutenghong@fjmu.edu.cn (T.L.); zly2021@fjmu.edu.cn (L.Z.); czj1201@fjmu.edu.cn (Z.C.); caishaojun@fjmu.edu.cn (S.C.)

**Keywords:** papillary thyroid carcinoma, *IL1β*, tumor progression, MAPK signaling pathway, *SMDT1*

## Abstract

**Background:** Papillary thyroid carcinoma (PTC) is the most common malignant tumor of the thyroid gland. Although most patients have favorable outcomes, a subset of patients develop aggressive disease characterized by lymph node metastasis and recurrence. Increasing evidence indicates that inflammatory factors participate in tumor progression. However, the expression pattern and functional role of interleukin-1 beta (IL1β) in PTC remain unclear. This study aimed to investigate the expression, biological function, and underlying molecular mechanisms of IL1β in PTC progression. **Methods:** IL1β expression and its clinical relevance were evaluated using public databases, 152 paired PTC and adjacent non-cancerous tissue samples, and PTC cell lines. Functional assays, including cell proliferation, colony formation, wound-healing, transwell invasion, and flow cytometry assays, were performed to investigate the effects of IL1β on malignant phenotypes. RNA sequencing, proteomic analysis, co-immunoprecipitation assays, rescue experiments, and a xenograft mouse model were conducted to identify and validate the downstream molecular mechanisms of IL1β. **Results:** IL1β expression was significantly reduced in PTC tissues and cell lines and was negatively associated with lymph node metastasis. Overexpression or recombinant protein treatment of IL1β inhibited PTC cell proliferation, migration, invasion, epithelial–mesenchymal transition, and tumor growth, while promoting apoptosis. Mechanistically, IL1β increased the expression of *SMDT1* gene and suppressed MAPK signaling activity by reducing MEK/ERK phosphorylation. Knockdown of *SMDT1* gene partially reversed the inhibitory effects of IL1β on PTC cell progression and MAPK pathway activation. **Conclusions:** This study demonstrates that IL1β functions as a tumor suppressor in PTC through regulation of the SMDT1-MAPK signaling axis. These findings provide new insights into the role of inflammatory regulation in thyroid cancer (THCA) progression and suggest that IL1β-related molecular pathways may represent potential biomarkers or therapeutic targets for aggressive PTC.

## Simple Summary

Papillary thyroid carcinoma (PTC) is the most common type of thyroid cancer (THCA), and although most patients have a good prognosis, some patients develop aggressive disease with lymph node metastasis and recurrence. Inflammation-related factors are increasingly recognized as important regulators of tumor development, but their roles in PTC remain unclear. In this study, we found that the inflammatory molecule interleukin-1 beta (IL1β) was reduced in PTC tissues and was associated with lymph node metastasis. Increasing IL1β levels inhibited tumor cell growth, migration, invasion, and promoted cancer cell death. Further investigation revealed that IL1β suppressed tumor progression by increasing *SMDT1* gene expression and reducing MAPK signaling activity. These findings provide new insights into how inflammatory signals regulate THCA progression and suggest potential biomarkers and therapeutic targets for patients with aggressive PTC.

## 1. Introduction

Papillary thyroid carcinoma (PTC) represents the predominant histological subtype of thyroid malignancies and accounts for the majority of thyroid cancer (THCA) cases [1]. Although most patients with PTC exhibit favorable clinical outcomes after standard treatment, a subset of patients develop aggressive disease characterized by cervical lymph node metastasis, local invasion, recurrence, and reduced survival [2]. The increasing use of thyroid ultrasonography and fine-needle aspiration has contributed to a continuous rise in PTC detection; however, accurately identifying patients at high risk of disease progression remains challenging [3]. Therefore, elucidating the molecular mechanisms underlying PTC aggressiveness is essential for improving risk stratification and developing more effective therapeutic strategies.

Accumulating evidence indicates that tumor progression is not solely determined by intrinsic genetic alterations but is also profoundly influenced by interactions between tumor cells and the surrounding microenvironment. Among these regulatory factors, chronic inflammation has emerged as a critical contributor to cancer initiation and progression by modulating tumor cell proliferation, apoptosis, invasion, metastasis, and immune responses [4]. The thyroid gland is particularly susceptible to inflammatory regulation due to its close association with autoimmune processes and immune-mediated disorders [5,6,7]. However, the precise molecular mechanisms by which inflammatory signals influence PTC progression remain incompletely understood.

Interleukin-1 beta (IL1β) is a classical pro-inflammatory cytokine that plays diverse and context-dependent roles in tumor biology. Through binding to the interleukin-1 receptor, IL1β activates multiple downstream signaling pathways, including nuclear factor kappa B (NF-κB) and mitogen-activated protein kinase (MAPK) pathways, thereby regulating inflammatory responses, cellular survival, and tumor behavior [8]. Previous studies have predominantly suggested that IL1β promotes tumor progression by enhancing invasion, angiogenesis, and immunosuppressive microenvironments in several malignancies [9,10,11,12]. However, increasing evidence indicates that the biological function of IL1β varies depending on tumor type and cellular context, and IL1β may also exert antitumor effects by inducing apoptosis or enhancing immune-mediated tumor suppression [13,14,15]. Nevertheless, the expression pattern, clinical significance, and functional role of IL1β in PTC remain largely unclear.

The MAPK signaling pathway is a central oncogenic pathway involved in THCA development and progression. Aberrant activation of MAPK signaling, particularly through alterations affecting the BRAF/MEK/ERK cascade, promotes THCA cell proliferation, survival, invasion, and dedifferentiation [16]. Accordingly, targeting key components of the MAPK pathway has become an important therapeutic strategy for advanced THCA [17,18]. Although inflammatory cytokines can regulate MAPK activity in various tumor contexts [14,19], whether IL1β regulates PTC progression through modulation of MAPK signaling remains unknown.

Mitochondrial homeostasis has recently been recognized as an important determinant of cancer cell fate by regulating cellular metabolism, calcium signaling, and apoptosis [20]. Single-pass membrane protein with aspartate-rich tail 1 (SMDT1), an essential component of the mitochondrial calcium uniporter complex, plays a critical role in mitochondrial calcium regulation and cellular stress responses [21,22]. Previous studies have suggested that abnormal SMDT1 expression may influence tumor cell proliferation and metastatic potential [23]; however, its biological function and regulatory mechanism in PTC remain poorly characterized. Whether SMDT1 serves as a molecular link between inflammatory signals and oncogenic pathways in THCA has not been investigated.

Based on these observations, we hypothesized that IL1β may regulate PTC progression through a previously unidentified molecular mechanism involving SMDT1 and MAPK signaling. In this study, we systematically investigated the expression pattern and biological function of IL1β in PTC using clinical specimens, cellular models, multi-omics analyses, and in vivo experiments. We further identified SMDT1 as a downstream mediator of IL1β and demonstrated that IL1β suppresses PTC progression through inhibition of MAPK signaling. Our findings reveal a previously unrecognized IL1β-SMDT1-MAPK regulatory axis in PTC and provide new insights into the context-dependent role of inflammatory factors in THCA progression.

## 2. Materials and Methods

### 2.1. Expression Analysis and Survival Analysis of IL1β and SMDT1 Gene in Pan-Cancer

We performed a pan-cancer expression analysis of *IL1β* and *SMDT1* gene using the Gene Expression Profiling Interactive Analysis 3 (GEPIA3) database (https://gepia3.bioinfoliu.com/; accessed on 1 January 2026), with special emphasis on THCA tumor tissues.

Survival analyses used The Cancer Genome Atlas (TCGA) dataset retrieved from the Home-for-Researchers platform (https://www.home-for-researchers.com/; access date: 25 August 2026). For *IL1β gene*, the surv_cutpoint function in the survminer package was used to calculate the optimal cut-off value for grouping, and its relationship with disease-specific survival (DSS) was assessed. For *SMDT1 gene*, samples were divided into high-expression and low-expression groups at the median, and its association with disease-free survival (DFS) was assessed. In both analyses, Kaplan–Meier (KM) curves were plotted, survival differences were assessed using the log-rank test, and hazard ratios (HRs) with 95% confidence intervals (CIs) were calculated by the univariate Cox regression.

### 2.2. Clinical Samples

From September 2024 to September 2025, 152 pairs of tumor tissues and matched paracancerous non-tumor thyroid tissues were obtained from PTC patients diagnosed and treated at the Fujian Medical University Union Hospital. All these tissues were immediately stored in liquid nitrogen until subsequent processing. The distance between the paracancerous normal tissues and the tumor was at least 3 cm. Before surgery, none of the patients had received radiotherapy, chemotherapy, or immunotherapy, and all had a single type of cancer.

### 2.3. Immune Infiltrate Analysis of IL1β in THCA

The Tumor Immune Estimation Resource (TIMER; https://cistrome.shinyapps.io/timer/; accessed on 1 January 2026) database was used to examine immune infiltrates in THCA. The relationships between IL1β expression and the abundance of immune infiltrates were assessed using Spearman’s correlation coefficients.

### 2.4. Quantitative Real-Time Polymerase Chain Reaction (qRT-PCR)

Detailed experimental protocols were reported in a previous study [24]. Total RNA was extracted from the samples using a rapid RNA extraction kit. RNA concentration and purity were determined with an ultraviolet spectrophotometer (Thermo Fisher Scientific, Wilmington, DE, USA). cDNA was synthesized via a reverse transcription kit, followed by qRT-PCR on a 7500 Real-Time PCR system (Applied Biosystems, Foster City, CA, USA). Relative gene expression was normalized to *β-actin*, and all reactions were performed with at least three biological replicates. Primer sequences are provided in Supplementary Table S1.

### 2.5. Immunohistochemical (IHC) and Immunohistochemical Score (H-SCORE)

Immunohistochemical staining was performed as previously described [25]. Briefly, 5 μm paraffin-embedded sections were dewaxed, rehydrated through graded ethanol, and rinsed in distilled water. After antigen retrieval and blocking, sections were incubated with primary antibodies overnight at 4 °C, incubated the following day with secondary antibodies, and developed with 3,3′-diaminobenzidine (DAB). Antibody details are provided in Supplementary Table S2. After hematoxylin counterstaining, the sections were dehydrated and mounted for microscopic examination. Images were acquired with a Leica microscope (Leica Microsystems, Wetzlar, Germany). Staining was scored semi-quantitatively by percentage of positive cells (0 = 0%, 1 = 1–24%, 2 = 25–49%, 3 = 50–74%, 4 = 75–100%) and by intensity (0 = negative, 1 = weak, 2 = moderate, 3 = strong). The final H-SCORE was calculated by multiplying the proportion score by the intensity score [26].

### 2.6. Cell Culture

PTC cell lines (BCPAP, KTC-1, and TPC-1) and the normal thyroid follicular epithelial cell line (Nthy-ori3-1) were all purchased from iCell Bioscience Inc. (Shanghai, China). Cells were cultured in RPMI-1640 medium (Gibco, Shanghai, China) supplemented with 10% fetal bovine serum (FBS) and 1% penicillin-streptomycin at 37 °C with 5% CO_2_.

### 2.7. Western Blotting (WB)

The protocol matched that of the previous study [25]. Total proteins were extracted from tissue and cell samples using Radioimmunoprecipitation Assay buffer (RIPA) lysis buffer. Protein concentrations were measured using a bicinchoninic acid (BCA) Protein Assay Kit (Beyotime, Haimen, China). Equal amounts of protein (20 μg) were separated by sodium dodecyl sulfate–polyacrylamide gel electrophoresis (SDS-PAGE) and transferred onto polyvinylidene fluoride (PVDF) membranes (Millipore, Billerica, MA, USA). After blocking with 5% BSA blocking buffer (Solarbio, Beijing, China), the membranes were incubated with primary antibodies at 4 °C overnight. The membranes were then incubated with secondary antibodies at room temperature for 1 h. Protein bands were visualized using a high-sensitivity chemiluminescence kit (Beyotime, Jiangsu, China), and band intensities were quantified using ImageJ software (version 2.0).

### 2.8. Cell Counting Kit-8 (CCK-8) Assay

The PTC cells were plated into a 96-well plate (2 × 10^3^ cells/well) and cultured overnight at 37 °C with 5% CO_2_. The recombinant IL1β protein (MCE, Monmouth Junction, NJ, USA) was added to the cells with a concentration gradient of 0–200 ng/mL. After 48 h, 10 µL of the CCK-8 reagent (Dojindo, Kumamoto, China) was added to each well, and the cells were incubated at 37 °C for 4 h. A multifunctional microplate reader (BioTek Instruments Inc., Winooski, VT, USA) was used to measure the absorbance of each well at 450 nm to calculate the cell viability and determine the half-maximal inhibitory concentration (IC_50_) of the recombinant IL1β protein.

To evaluate the proliferation of cells over time, the treated cells were seeded into 96-well plates (2 × 10^3^ cells/well). The CCK-8 reagent was used to detect the cells at 24, 48, 72, and 96 h, respectively. A microplate reader was used to record the absorbance values at 450 nm.

### 2.9. Cell Treatment

BCPAP cells were seeded in 6-well plates (5 × 10^4^ cells/well), after treatment with serum-free RPMI 1640 medium, and incubated overnight at 37 °C with 5% CO_2_. The following day, different doses of the recombinant IL1β protein, IL-1 receptor antagonist (IL-1RA) (MCE, USA) or the MAPK activator C16-PAF (MCE, USA) were added to the cells.

### 2.10. Wound-Healing Assay

PTC cells were seeded in 6-well plates (5 × 10^5^ cells/well) and cultured to 90% confluence. Wounds were scratched using a 200 µL plastic pipette tip. After phosphate-buffered saline (PBS) wash performed 2–3 times, cells were maintained in serum-free RPMI 1640 medium for the duration of the assay. Wounded areas were photographed by microscopy at 0 h and 24 h, respectively.

### 2.11. Transwell Assay

After overnight incubation in serum-free medium, the cell concentration was adjusted to 2 × 10^5^ cells/mL. 200 µL Cells were seeded into Matrigel-coated Transwell chambers with serum-free medium, while the lower chamber contained 500 µL complete medium with 10% FBS. After incubation, invaded cells were fixed with 4% paraformaldehyde and visualized by crystal violet staining. Invasion was quantified as the average number of cells in five randomly selected microscopic fields.

### 2.12. Cell Transfection

To knock down the expression of specific genes in cells, PTC cells were seeded into 6-well plates at a density of 5 × 10^4^ cells per well and cultured overnight in complete medium to reach approximately 70–80% confluence. Transfection was performed using Lipofectamine 3000 (Invitrogen, Carlsbad, CA, USA), and short interfering RNAs (siRNAs) specifically targeting the genes were introduced into the cells. Cells were harvested for further analysis 48 h after transfection. For gene overexpression experiments, PTC cells were seeded into 24-well plates at 2 × 10^4^ cells/well and allowed to attach overnight until reaching approximately 20–30% confluency before viral transduction. Transfection was performed using HitransG P infection enhancer, overexpression lentivirus, and serum-free medium. After 16 h of culture, the medium was replaced with complete medium for continued culture for 2–3 days. Subsequently, 2 µg/mL of puromycin was added to each well to select stably transfected cells.

All siRNAs required for this study were purchased from GenePharma Co., Ltd. (Shanghai, China), and the sequences are detailed in Supplementary Table S3. The overexpression lentivirus encoding *IL1β* gene was purchased from Genechem Inc. (Shanghai, China). The transcript is NM_000576.3, the vector number is GV341, and the element order is Ubc-MCS-3FLAG-SV40-puromycin. Transfection procedures were conducted according to established experimental protocols. The efficiency of gene modulation was subsequently confirmed via qRT-PCR and WB analyses.

### 2.13. Colony Formation Assays

Cells (1000 per well) were plated in culture plates for 2 weeks at 37 °C in a humidified environment with 5% CO_2_ and stained with 1% crystal violet staining solution for 10–20 min. The stained colonies were imaged using a camera and counted using a microscope.

### 2.14. Flow Cytometry

Flow cytometry was employed to analyze cell apoptosis. PTC cells were seeded into 6-well plates at a density of 5 × 10^5^ cells per well and cultured overnight. After digestion with trypsin without ethylenediaminetetraacetic acid (EDTA) and washing with PBS, 5 µL of propidium iodide (PI) and 5 µL of Annexin V-fluorescein isothiocyanate (FITC) solution were added. The cells were incubated at room temperature in the dark for 15 min before apoptosis analysis. Subsequently, the staining status was detected using a BD Accuri C6 Plus flow cytometer (BD Biosciences, San Jose, CA, USA), and the results were analyzed with FlowJo software (version 10.10).

### 2.15. RNA-Sequencing (RNA-seq) Analysis

Total RNA was extracted from cells in the control group (Vector) and the *IL1β* gene overexpression group (oe-*IL1β*), and the samples were sent to Novogene Co., Ltd. (Beijing, China) for RNA-seq analysis. Five biological replicate samples were set for each cell line in each group to ensure the reliability of the data. Bioinformatics analysis was performed as previously described [25]. Differentially expressed genes were identified using DESeq2 (version 1.48.1). To account for multiple hypothesis testing, raw *p* values were adjusted using the Benjamini–Hochberg procedure to control the false discovery rate (FDR). Genes with an adjusted *p* value (FDR) < 0.05 and the predefined fold-change threshold were considered significantly differentially expressed.

### 2.16. Proteomic Analysis

To identify potential downstream proteins regulated by IL1β in PTC cells, quantitative proteomic analysis was performed using cells from the control group (Vector) and the *IL1β gene* overexpression group (oe-*IL1β*). Total cellular proteins were extracted from each group, and three independent biological replicates were prepared for each condition. The extracted protein samples were subsequently submitted to Genechem Inc. (Shanghai, China) for proteomic profiling.

#### 2.16.1. Mass Spectrometry Analysis

The peptides from each sample were analyzed by Orbitrap^TM^ Astral^TM^ mass spectrometer connected to a Vanquish Neo system liquid chromatography (Thermo Fisher Scientific, Bremen, Germany) in the data-independent acquisition (DIA) mode.

#### 2.16.2. Data Analysis

Proteomic data were processed by Spectronaut (Biognosys AG, Schlieren, Zurich, Switzerland). Identification was controlled at a Q-value cutoff of 1% at the precursor and protein levels. For differential protein expression analysis, *p* values obtained from comparisons between experimental groups were corrected for multiple testing using the Benjamini–Hochberg procedure. Proteins meeting the predefined fold-change threshold and an adjusted *p* value (FDR) < 0.05 were considered significantly differentially expressed. The database used in this project is: uniprot_homo_20250416_205003_9606_all.

### 2.17. Co-Immunoprecipitation (Co-IP) Assays

The control group (Vector) cells and the *IL1β gene* overexpression group (oe-*IL1β*) cells were lysed using IP lysis buffer containing protease inhibitors (Thermo Fisher Scientific, USA). The lysates were incubated with antibodies or immunoglobulin G (IgG) as a negative control overnight at 4 °C to form immune complexes. The specific antibodies required are also detailed in Supplementary Table S2. Then, Protein A/G agarose beads (Thermo Fisher Scientific, USA) were added and incubated at 4 °C for 4 h, followed by washing with the lysis buffer. Finally, the detection was performed by WB.

### 2.18. Nude Mouse Xenograft Tumor Model

Twelve male BALB/c nude mice, aged 3–5 weeks, were purchased and acclimated in a specific pathogen-free (SPF) animal room for one week. They were then randomly assigned to two groups of six mice each. KTC-1 cells were transfected with a lentivirus overexpressing *IL1β* gene (oe-*IL1β*) or with an empty vector (Vector). Next, 200 µL of normal saline containing 2 × 10^6^ tumor cells was injected subcutaneously into the right flank of each mouse to establish xenograft tumors. Tumor growth was monitored closely, mice were euthanized if tumor volume exceeded 2000 mm^3^, if the tumor reached 10% of body weight, if body weight fell by more than 20–25%, or if signs of cachexia or severe distress appeared. Tumor volume was measured every 5 days for 4 consecutive weeks and calculated as volume = 0.5 × length × width^2^. After 4 weeks, the nude mice were euthanized under deep anesthesia induced by intraperitoneal injection of 1% sodium pentobarbital at 0.1–0.2 mL per 10 g body weight (100–200 mg/kg). Deep anesthesia was confirmed by a relaxed posture and the absence of a hind-limb pinch response, and euthanasia was completed by cervical dislocation. Tumors were excised, weighed, and stored for subsequent analyses.

### 2.19. Statistical Analysis

All data were analyzed using GraphPad Prism 8.0.2 and R (version 4.5.2). The normality of continuous data was assessed using the Shapiro–Wilk test before parametric statistical analyses. Statistical significance between two groups was determined using Student’s *t*-test, while one-way ANOVA was used for multiple group comparisons. The chi-square test was used to analyze the correlation between *IL1β* gene expression levels and clinical pathological characteristics. Each experiment was independently performed with at least three biological replicates. Quantitative results are expressed as mean ± standard deviation (SD). Differences were considered statistically significant when the two-sided *p* value was below 0.05.

## 3. Results

### 3.1. Expression Analysis and Survival Analysis of the IL1β Gene in THCA

**Figure 1:**
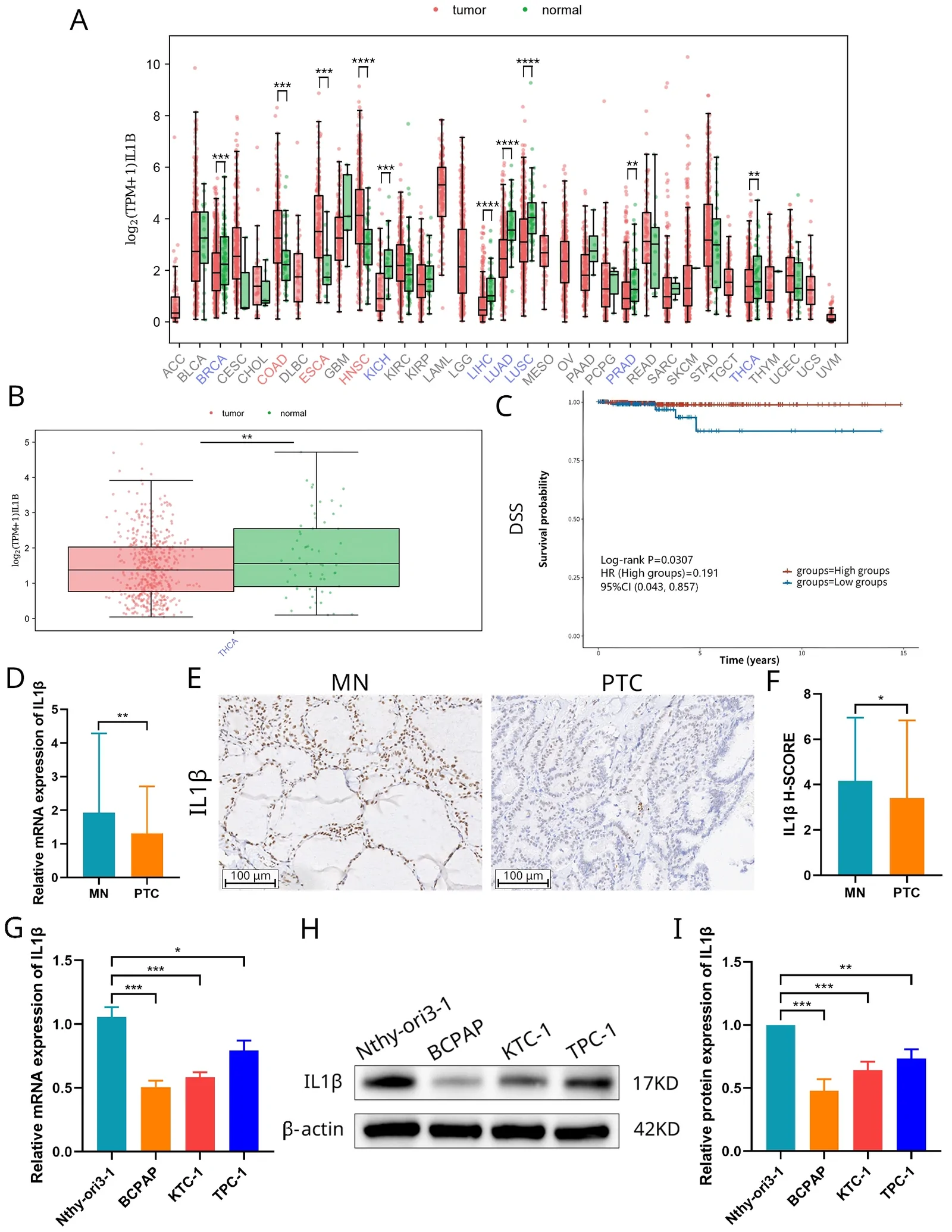
Expression analysis and survival analysis of the *IL1β* gene in THCA. (**A**) *IL1β* gene expression across tumor types. Red text color indicates significantly high expression of *IL1β* gene in the tumor, while blue text color indicates significantly low expression of *IL1β* gene in the tumor. (**B**) *IL1β* gene expression in THCA. (**C**) KM survival curves for DSS associated with *IL1β* gene expression levels. (**D**) *IL1β* mRNA levels in PTC and MN tissues measured by qRT-PCR (mean ± SD, n = 152). (**E**) IL1β protein levels in PTC and MN tissues assessed by IHC (mean ± SD, n = 152). (**F**) Comparison of IHC scores (H-SCORE) for IL1β protein in PTC and MN tissues (mean ± SD, n = 152). (**G**) *IL1β* mRNA levels in a normal thyroid follicular epithelial cell line (Nthy-ori3-1) and PTC cell lines (BCPAP, KTC-1, and TPC-1) measured by qRT-PCR (mean ± SD, n = 3). (**H**,**I**) IL1β protein levels in the Nthy-ori3-1 cell line and PTC cell lines assessed by WB (mean ± SD, n = 3). * indicates *p* < 0.05; ** indicates *p* < 0.01; *** indicates *p* < 0.001; **** indicates *p* < 0.0001. The whole blots (uncropped blots) are shown in the Supplementary Files.

Based on the TCGA dataset from the GEPIA3 database, a pan-cancer expression analysis of *IL1β* gene was conducted. The complete data are presented in Supplementary Table S4. The results demonstrated that there were significant differences in the expression of *IL1β* gene across various cancer types, with significant downregulation in THCA (*p* = 0.00868) (Figure 1A,B). Using the TCGA dataset via the Home-for-Researchers platform, the correlation between *IL1β* gene expression and DSS in THCA patients was assessed using KM survival analysis. THCA patients with high *IL1β* gene expression exhibited significantly better DSS compared with those with low expression (*p* = 0.0307) (Figure 1C).

Consistently, qRT-PCR and IHC analyses of 152 paired clinical samples confirmed significantly lower IL1β expression in PTC tissues than in matched non-tumorous tissues (MN) (Figure 1D–F). Similar downregulation was observed in PTC cell lines, particularly BCPAP and KTC-1, which were selected for subsequent experiments (Figure 1G–I).

According to the criteria of the eighth edition of the TNM staging system (TNM-8) jointly developed by the American Joint Committee on Cancer (AJCC) and the Union for International Cancer Control (UICC) [27], using 2^−ΔΔCT^ > 1 of the *IL1β* gene in PTC tissue as the cut-off value for the expression level, the samples were divided into a high-expression group and a low-expression group to analyze the correlation between the *IL1β* gene expression level and clinicopathological features. As shown in Supplementary Table S5, the expression level of *IL1β* gene was significantly negatively correlated with the number of lymph node metastases (*p* = 0.0055), suggesting that IL1β may act as a potential tumor suppressor factor in PTC.

### 3.2. Correlation Between IL1β Expression and Immune Cell Infiltration in THCA

We also examined the relationship between IL1β expression and six types of tumor-infiltrating immune cells using the TIMER database. As shown in Supplementary Figure S1, IL1β expression correlated significantly with B cells (r = 0.351, *p* = 2.12 × 10^−15^), CD8^+^ T cells (r = 0.32, *p* = 4.53 × 10^−13^), CD4^+^ T cells (r = 0.172, *p* = 1.29 × 10^−4^), macrophages (r = 0.285, *p* = 1.43 × 10^−10^), neutrophils (r = 0.541, *p* = 1.88 × 10^−38^), and dendritic cells (r = 0.572, *p* = 1.97 × 10^−43^). These findings suggest that IL1β detected in THCA may originate from both tumor cells and infiltrating immune cells, highlighting the complexity of IL1β regulation within the tumor microenvironment.

### 3.3. Recombinant IL1β Protein Can Inhibit the Survival and Metastatic Ability of BCPAP Cells

**Figure 2:**
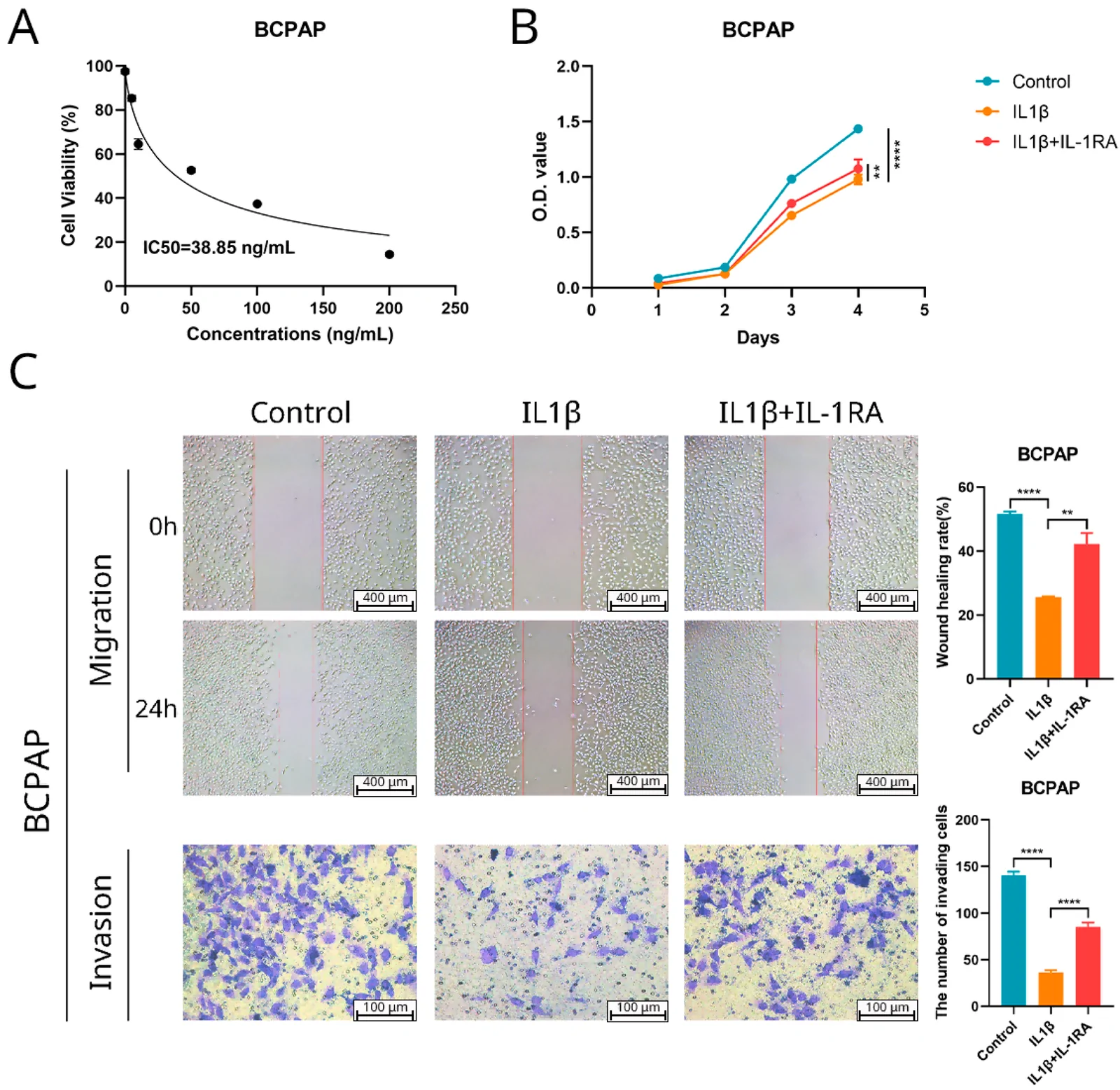
Effects of recombinant IL1β protein on the malignant phenotype of BCPAP cells. (**A**) Effect of recombinant IL1β protein on the viability of BCPAP cells (mean ± SD, n = 3). (**B**) Effects of recombinant IL1β protein and IL-1RA on the proliferation ability of BCPAP cells (mean ± SD, n = 3). (**C**) Effects of recombinant IL1β protein and IL-1RA on the migration and invasion abilities of BCPAP cells (mean ± SD, n = 3). ** indicates *p* < 0.01; **** indicates *p* < 0.0001.

Then, we treated the THCA cell line BCPAP with recombinant IL1β protein at concentrations ranging from 0 to 200 ng/mL to activate extracellular IL1β receptor-dependent signaling, and assessed cell viability. The CCK-8 assay revealed a dose-dependent inhibition of viability, with an IC_50_ of 38.85 ng/mL, which was used for subsequent experiments (Figure 2A).

Functional assays demonstrated that recombinant IL1β protein significantly suppressed BCPAP cell proliferation, migration, and invasion, whereas these effects were partially reversed by the IL-1RA (Figure 2B,C).

### 3.4. IL1β Suppresses the Proliferation, Migration, and Invasion Abilities of PTC Cells

**Figure 3:**
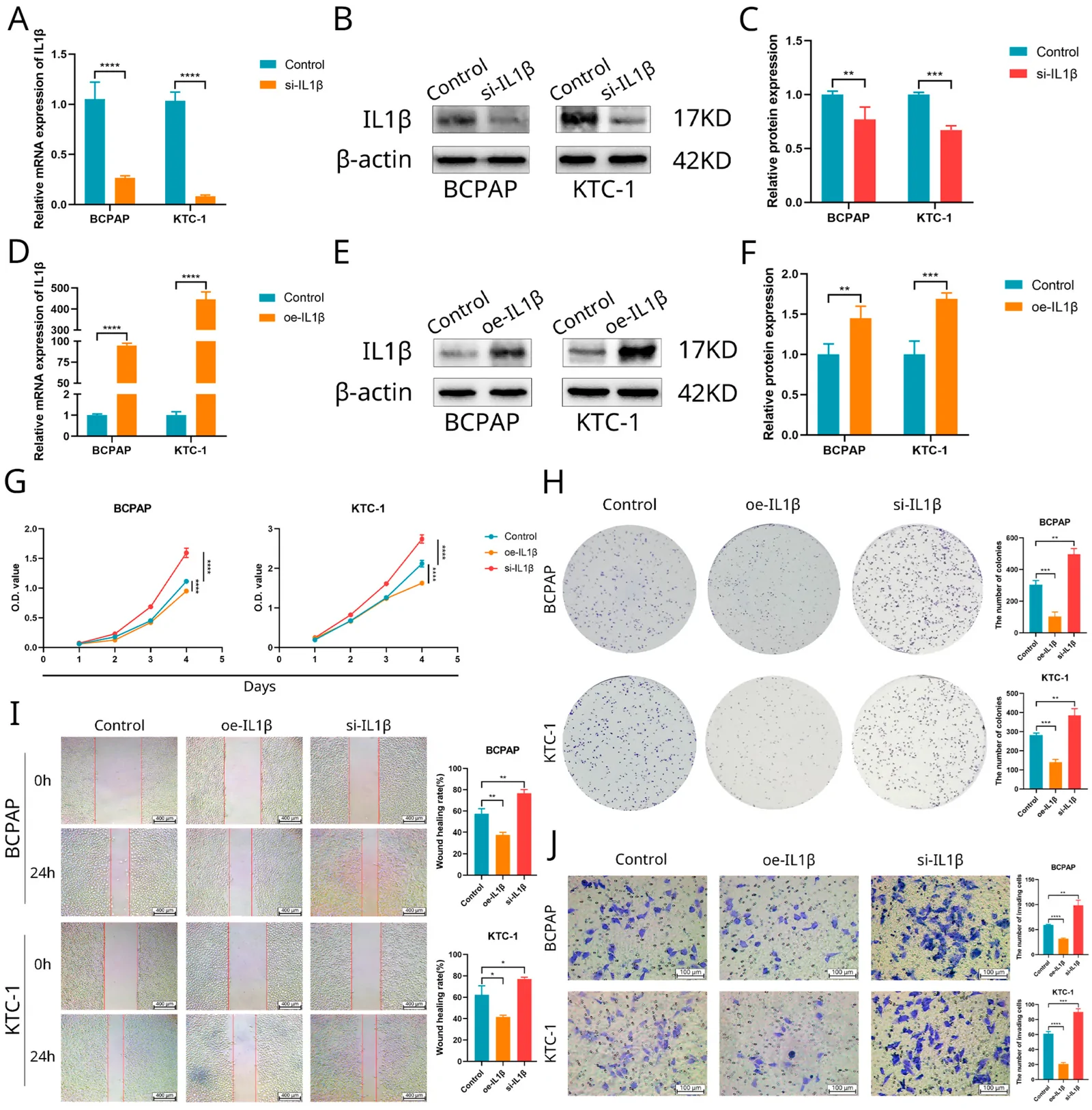
Inhibitory effect of IL1β on the malignant phenotype of PTC cells. (**A**–**C**) The transfection efficiency of siRNA was detected by qRT-PCR and WB (mean ± SD, n = 4). (**D**–**F**) The transfection efficiency of overexpression lentivirus was detected by qRT-PCR and WB (mean ± SD, n = 4). (**G**) The effect of IL1β on cell proliferation ability was detected by CCK-8 assay (mean ± SD, n = 3). (**H**) The effect of IL1β on cell colony formation ability was detected by colony formation assay (mean ± SD, n = 3). (**I**) The effect of IL1β on cell migration ability was detected by wound-healing assay (mean ± SD, n = 3). (**J**) The effect of IL1β on cell invasion ability was detected by transwell assay (mean ± SD, n = 3). * indicates *p* < 0.05; ** indicates *p* < 0.01; *** indicates *p* < 0.001; **** indicates *p* < 0.0001. The whole blots (uncropped blots) are shown in the Supplementary Files.

To distinguish intracellular IL1β expression effects from extracellular IL1β-mediated signaling, we generated control group (Control), *IL1β* gene knockdown group (si-*IL1β*), and *IL1β* gene overexpression group (oe-*IL1β*) lines by transfecting BCPAP and KTC-1 cells with siRNA or lentivirus. Transfection efficiency was assessed by qRT-PCR and WB. The *IL1β* gene expression was significantly reduced in the si-*IL1β* group (Figure 3A–C), and was significantly elevated in the oe-*IL1β* group compared with Control group (Figure 3D–F).

Functionally, CCK-8 assays showed that, compared with the Control group, *IL1β* gene overexpression significantly inhibited cell proliferation, whereas *IL1β* gene knockdown promoted proliferation (Figure 3G). Colony formation assays confirmed the growth-inhibitory effect of IL1β on PTC cells. Compared with the Control group, the number of colonies were markedly reduced in the oe-*IL1β* group but increased in the si-*IL1β* group (Figure 3H).

Given that tumor metastasis is the main cause of poor prognosis in THCA patients [28], we examined how IL1β modulates PTC cell migration and invasion using wound-healing and transwell invasion assays. In the wound-healing assay, *IL1β* gene overexpression significantly reduced the migration rate relative to the Control group, whereas *IL1β* gene knockdown significantly increased it (Figure 3I). Similarly, transwell invasion assay revealed fewer invading cells in the oe-*IL1β* group, and more in the si-*IL1β* group compared with the Control group (Figure 3J).

### 3.5. IL1β Induces Apoptosis in PTC Cells

**Figure 4:**
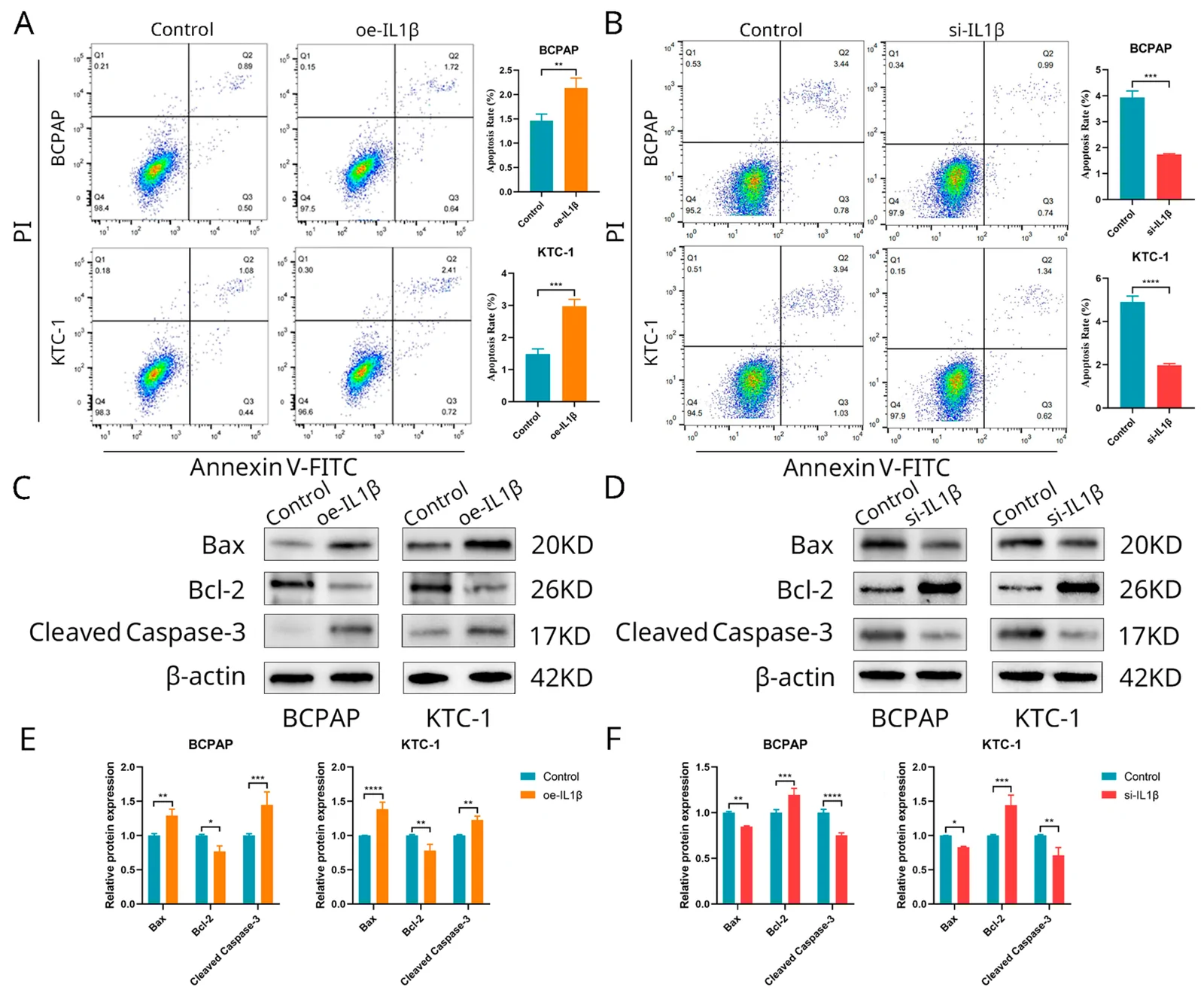
Effect of the IL1β on the apoptosis of PTC cells. (**A**,**B**) The effect of IL1β on the apoptosis of PTC cells was detected by flow cytometry (mean ± SD, n = 3). (**C**–**F**) The effect of IL1β on the expression levels of apoptosis-related proteins in PTC cells was detected by WB (mean ± SD, n = 4). * indicates *p* < 0.05; ** indicates *p* < 0.01; *** indicates *p* < 0.001; **** indicates *p* < 0.0001. The whole blots (uncropped blots) are shown in the Supplementary Files.

We assessed IL1β’s effect on PTC cell apoptosis by flow cytometry. Compared with the Control group, *IL1β* gene overexpression markedly increased the apoptosis rate, whereas *IL1β* gene knockdown significantly reduced it (Figure 4A,B). We then examined apoptosis-related protein levels by WB. Relative to Control, the oe-*IL1β* group showed significant upregulation of pro-apoptotic Bax and Cleaved Caspase-3, and significant downregulation of anti-apoptotic Bcl-2, while the opposite pattern was observed in the si-*IL1β* group (Figure 4C–F).

### 3.6. IL1β Involves in Regulating the Expression of Epithelial–Mesenchymal Transition (EMT)-Related and Matrix Metalloproteinases (MMPs) Proteins in PTC Cells

**Figure 5:**
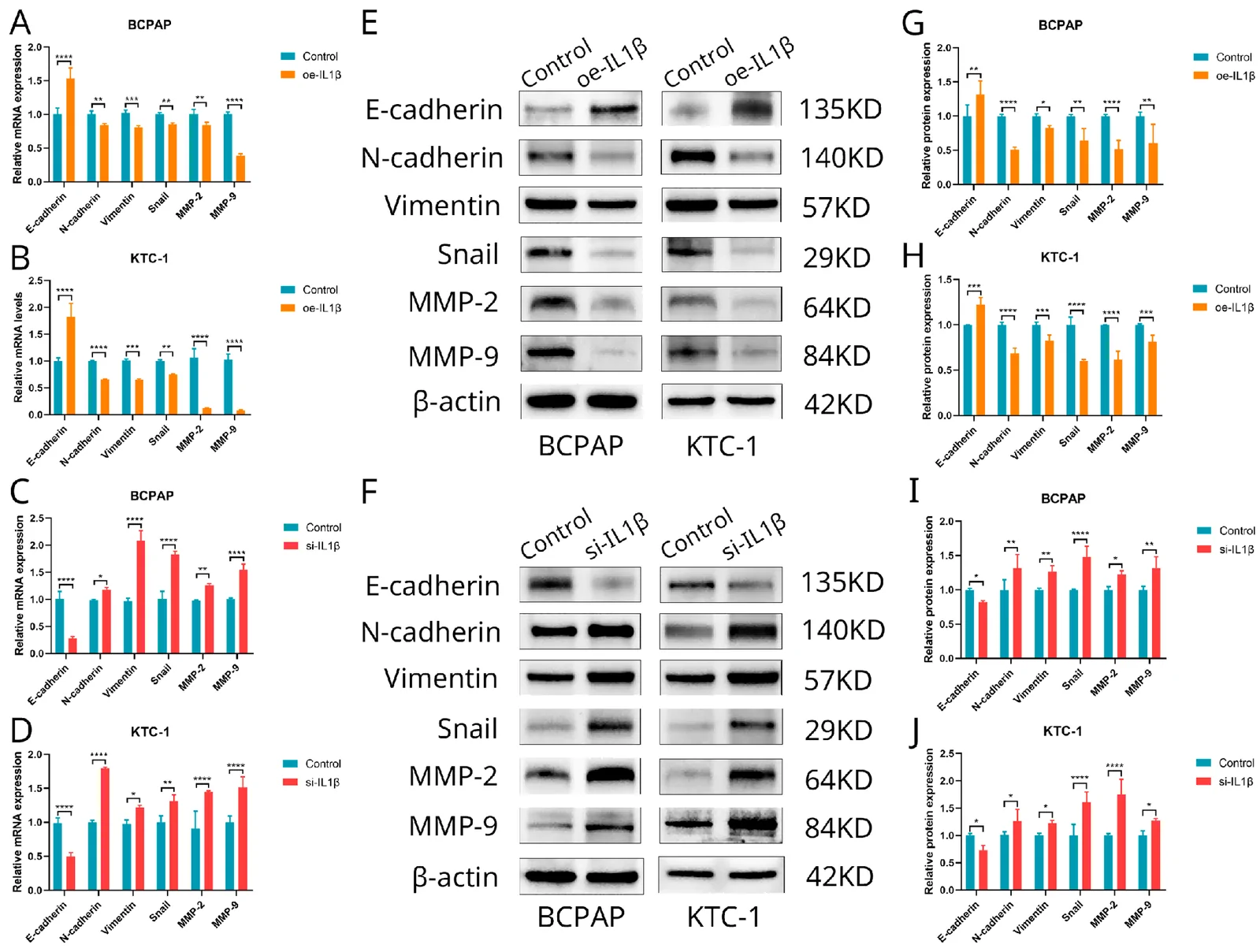
IL1β is involved in regulating the expression of EMT-related proteins and MMPs in PTC cells. (**A**–**D**) The effect of IL1β on the expression levels of EMT-related proteins and MMPs in PTC cells was detected by qRT-PCR (mean ± SD, n = 4). (**E**–**J**) The effect of IL1β on the expression levels of EMT-related proteins and MMPs in PTC cells was detected by WB (mean ± SD, n = 4). * indicates *p* < 0.05; ** indicates *p* < 0.01; *** indicates *p* < 0.001; **** indicates *p* < 0.0001. The whole blots (uncropped blots) are shown in the Supplementary Files.

EMT activation and MMPs overexpression are key drivers of tumor invasion and metastasis [29]. To examine how IL1β influences these processes in PTC cells, we measured EMT-related and MMPs mRNA and proteins by qRT-PCR and WB. Compared with the Control group, *IL1β* gene overexpression decreased mRNA and protein levels of N-Cadherin, Vimentin, Snail, MMP-2, and MMP-9 while increasing the expression of E-Cadherin. Conversely, *IL1β* gene knockdown produced the opposite expression pattern for these genes (Figure 5A–J). The concordant changes at the mRNA and protein levels support a role for IL1β in suppressing PTC progression and metastasis.

### 3.7. RNA-seq Reveals the Downstream Signaling Pathways Regulated by IL1β

**Figure 6:**
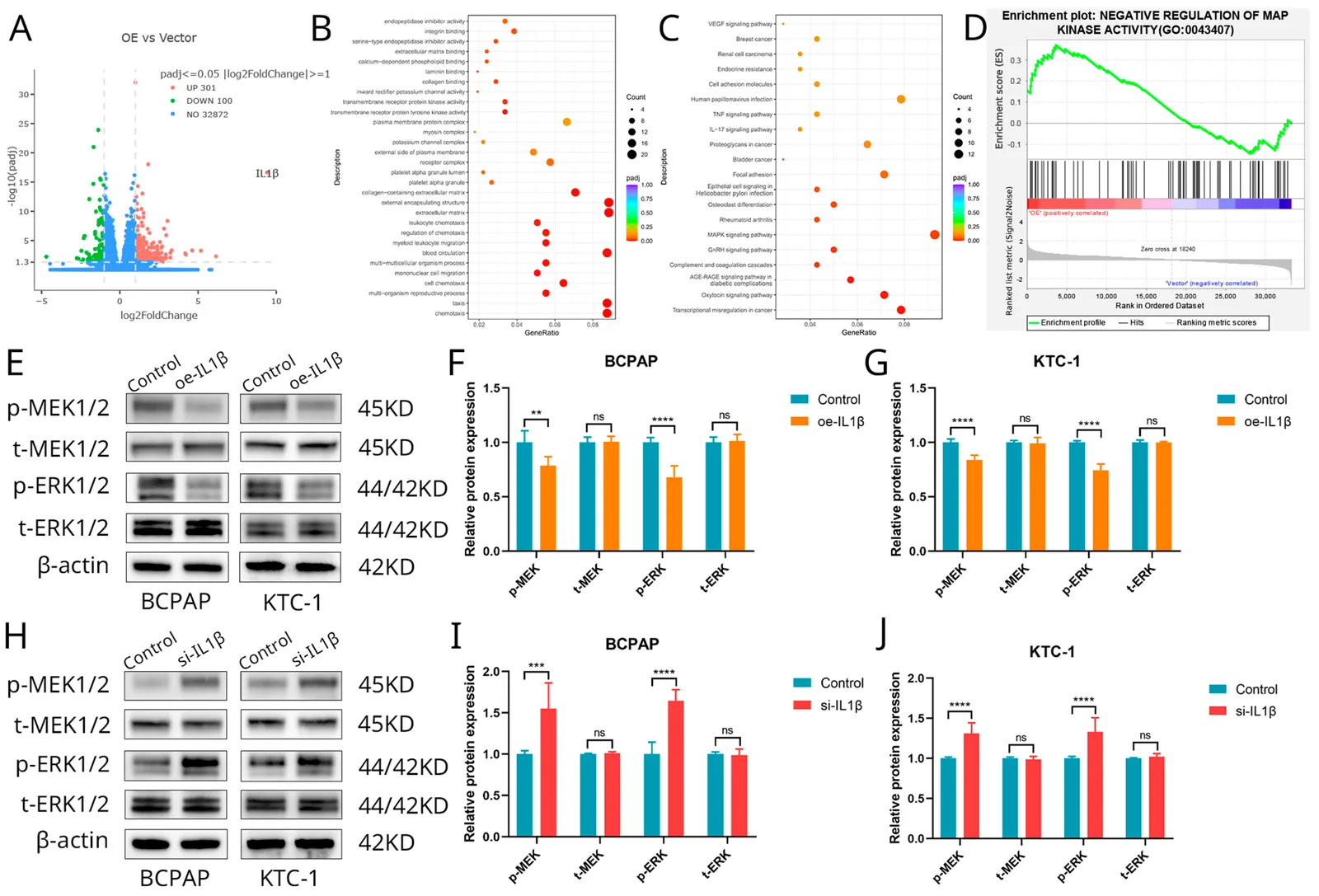
RNA-seq reveals and validates the downstream signaling pathways regulated by IL1β. (**A**) The volcano plot shows the DEGs obtained from RNA-seq of cells in the Vector group and the oe-*IL1β* group (mean ± SD, n = 5). Red indicates significantly upregulated genes, green indicates significantly downregulated genes, and blue indicates genes with no significant difference. The screening criteria were *p* ≤ 0.05 and |log_2_FC| ≥ 1 (FC, fold change). (**B**) GO functional enrichment analysis of DEGs. (**C**) KEGG pathway enrichment analysis of DEGs. (**D**) GSEA shows a significant negative correlation between *IL1β* gene overexpression and MAPK pathway activity. (**E**–**J**) The effect of IL1β on the expression levels of proteins related to the MAPK signaling pathway was detected by WB (mean ± SD, n = 4). ns indicates *p* > 0.05; ** indicates *p* < 0.01; *** indicates *p* < 0.001; **** indicates *p* < 0.0001. The whole blots (uncropped blots) are shown in the Supplementary Files.

To assess how *IL1β* gene overexpression alters the transcriptome of PTC cells, we performed RNA-seq on the control group (Vector) and the oe-*IL1β* group cells. After data normalization, we identified 401 differentially expressed genes (DEGs) between the two groups (Figure 6A). Gene Ontology (GO) enrichment analysis of these DEGs revealed significant overrepresentation of biological processes (Figure 6B). Kyoto Encyclopedia of Genes and Genomes (KEGG) pathway enrichment highlighted multiple signaling pathways (Figure 6C), with the MAPK signaling pathway notably enriched among the DEGs. Gene set enrichment analysis (GSEA) further indicated that MAPK pathway activity is negatively correlated with *IL1β* gene overexpression (Figure 6D). These results provide important leads for further mechanistic studies of IL1β in PTC.

To assess IL1β’s impact on the MAPK pathway, we measured MAPK-related protein levels by WB in the Control group, the oe-*IL1β* group, and the si-*IL1β* group cells. Compared with Control, *IL1β* gene overexpression significantly reduced p-MEK1/2 and p-ERK1/2 protein levels (Figure 6E–G). Conversely, *IL1β* gene knockdown produced a significant increase in p-MEK1/2 and p-ERK1/2 levels (Figure 6H–J). Additionally, we utilized the MAPK activator C16-PAF and found that it could significantly upregulate the expression levels of p-MEK1/2 and p-ERK1/2 proteins in BCPAP cells at a concentration of 10.0 ng/mL (Supplementary Figure S2A,B). In functional experiments, the oe-*IL1β* group could notably suppress the proliferation, migration, and invasion abilities of BCPAP cells. However, when C16-PAF was used in combination, the anti-cancer effect of IL1β was partially reversed (Supplementary Figure S2C,D).

### 3.8. Proteomic Analysis Reveals the Downstream Mediator Genes Regulated by IL1β

**Figure 7:**
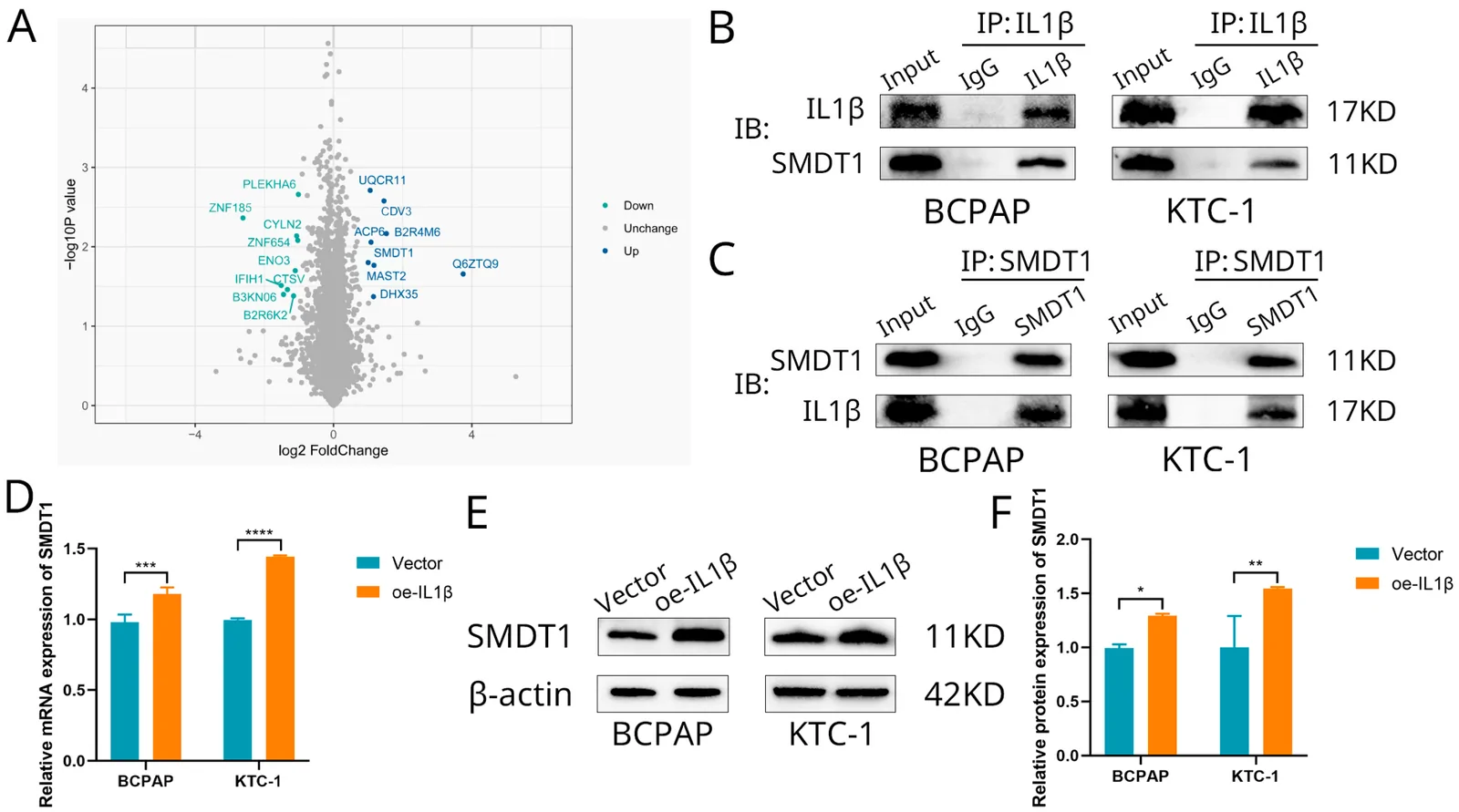
Proteomic analysis and screening of downstream mediator genes regulated by IL1β. (**A**) The volcano plot shows the DEGs obtained from proteomic analysis of cells in the Vector group and the oe-*IL1β* group (mean ± SD, n = 3). Blue represents significantly upregulated genes, green represents significantly downregulated genes, and grey represents genes with no significant difference. The screening criteria were *p* ≤ 0.05 and |log_2_FC| ≥ 1. (**B**,**C**) The association between IL1β and SMDT1 was verified by Co-IP experiments (mean ± SD, n = 3). (**D**–**F**) The effects of *IL1β* gene overexpression on the mRNA and protein expression levels of *SMDT1* were detected by qRT-PCR and WB (mean ± SD, n = 4). * indicates *p* < 0.05; ** indicates *p* < 0.01; *** indicates *p* < 0.001; and **** indicates *p*< 0.0001. The whole blots (uncropped blots) are shown in the Supplementary Files.

To assess how IL1β overexpression alters protein expression in PTC cells, we performed proteomic analysis comparing the Vector group and the oe-*IL1β* group cells. After normalizing the sequencing data, we identified 17 differentially expressed genes (DEGs) between the groups (Figure 7A). Among these, *SMDT1* showed a significant change (FC > 2, *p* < 0.05), which aligns with the tumor-suppressive effects of *IL1β* gene overexpression. In addition, our previous research showed that upregulating *SMDT1* gene expression can suppress the aggressive behavior of PTC cells [30]. Hence, we have chosen SMDT1 as a potential downstream mediator of the IL1β signaling for further study.

To assess the clinical significance of SMDT1 in THCA, we analyzed its expression and prognostic value using the TCGA dataset. *SMDT1* gene was markedly downregulated in THCA (P = 2.32 × 10^−8^) and correlated with patient DFS (*p* = 0.00403) (Supplementary Figure S3), supporting its role in THCA progression. To investigate whether SMDT1 is associated with IL1β-mediated regulation, we performed Co-IP assays in PTC cells. The results showed that IL1β and SMDT1 were detected in the same immunoprecipitated complex, suggesting a potential molecular association between IL1β and SMDT1 (Figure 7B,C). We then measured *SMDT1* gene expression in the Vector group and the oe-*IL1β* group cells by qRT-PCR and WB. Both *SMDT1* mRNA and protein levels were significantly higher in the oe-*IL1β* group (Figure 7D–F), consistent with the sequencing data. These results indicate that IL1β may positively regulate *SMDT1* expression in PTC cells.

### 3.9. Knockdown of SMDT1 Partially Reverses the Inhibitory Effect of IL1β on the Malignant Phenotype and MAPK Pathway Activity of PTC Cells

To examine the functional relationship between IL1β and SMDT1 in PTC cells, we knocked down *SMDT1* gene with siRNA in BCPAP and KTC-1 cells. The qRT-PCR and WB confirmed that, relative to the Control group, *SMDT1* mRNA and protein levels were substantially reduced after siRNA transfection (Supplementary Figure S4A–C). We then applied siRNA to knock down *SMDT1* gene in the oe-*IL1β* group cells, which likewise produced a marked reduction in *SMDT1* mRNA and protein expression (Supplementary Figure S4D–F).

**Figure 8:**
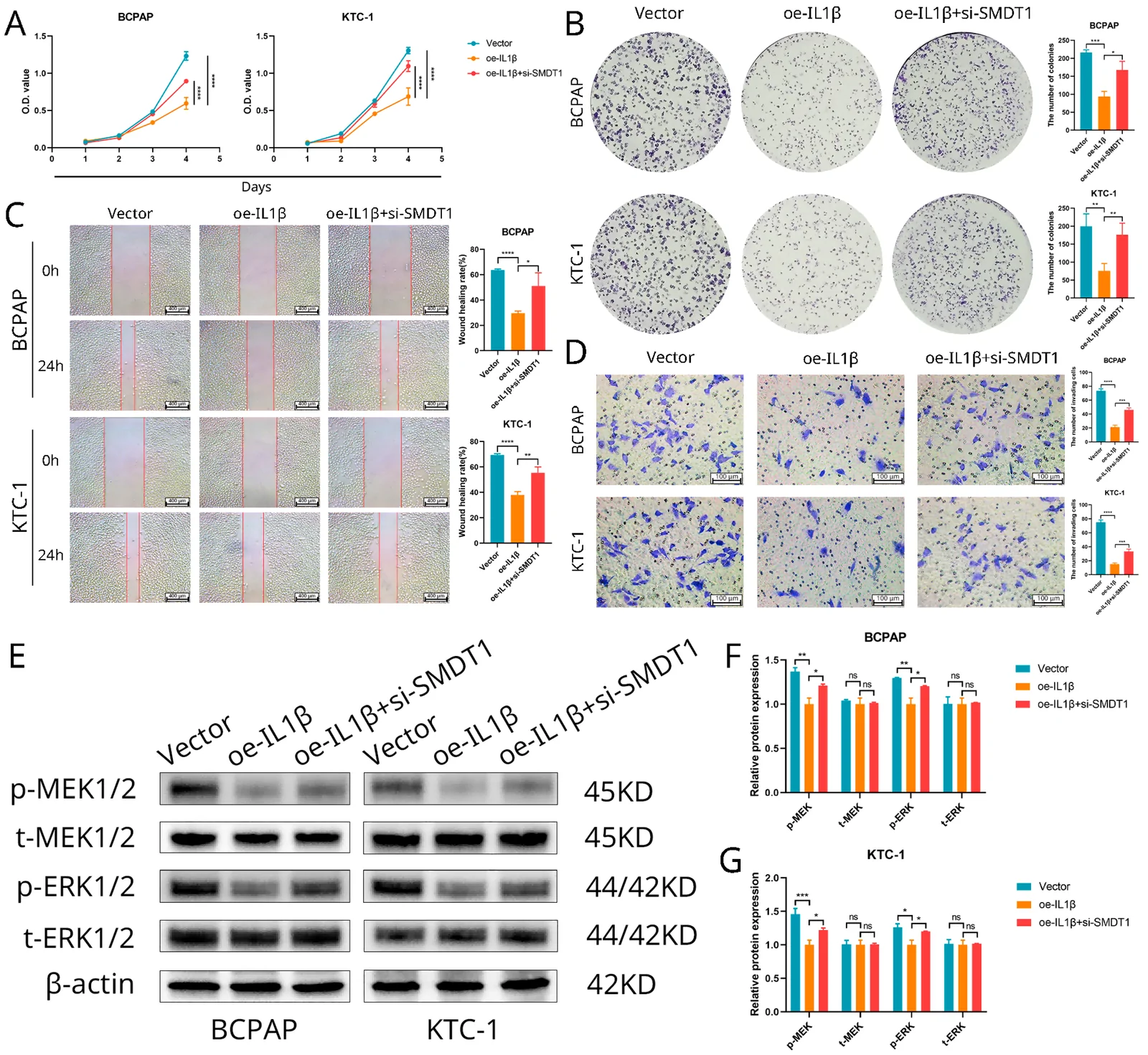
SMDT1 is involved in mediating the regulation of IL1β on the malignant phenotype and MAPK pathway activity of PTC cells. (**A**) The role of SMDT1 in the IL1β-mediated inhibition of cell proliferation ability was detected by CCK-8 assay (mean ± SD, n = 3). (**B**) The role of SMDT1 in the IL1β-mediated inhibition of cell colony formation ability was detected by colony formation assay (mean ± SD, n = 3). (**C**) The role of SMDT1 in the IL1β-mediated inhibition of cell migration ability was detected by wound-healing assay (mean ± SD, n = 3). (**D**) The role of SMDT1 in the IL1β-mediated inhibition of cell invasion ability was detected by transwell assay (mean ± SD, n = 3). (**E**–**G**) The role of SMDT1 in the IL1β-mediated inhibition of MAPK pathway activity was detected by WB (mean ± SD, n = 4). ns indicates *p* > 0.05; * indicates *p* < 0.05; ** indicates *p* < 0.01; *** indicates *p* < 0.001; and **** indicates *p* < 0.0001. The whole blots (uncropped blots) are shown in the Supplementary Files.

As shown by CCK-8 and colony formation assays, knockdown of *SMDT1* gene partially reversed IL1β’s inhibitory effects on PTC cell proliferation and colony formation (Figure 8A,B). Wound-healing and transwell invasion assays likewise showed that *SMDT1* gene knockdown partially reversed IL1β’s inhibitory effects on PTC cell migration and invasion (Figure 8C,D). These data indicate that SMDT1 mediates IL1β’s effects on PTC cell proliferation, migration, and invasion, revealing a functional interaction between the two genes. To assess SMDT1’s role in IL1β-regulated MAPK signaling, we measured the expression of key pathway molecules in PTC cells via WB. The results showed that the knockdown of *SMDT1* gene partially reversed IL1β’s inhibitory effect on MAPK pathway activity (Figure 8E–G). These results indicate that SMDT1 is critical for regulation of the IL1β-mediated MAPK signaling cascade in PTC cells.

### 3.10. Overexpression of IL1β Suppresses Tumor Growth In Vivo

**Figure 9:**
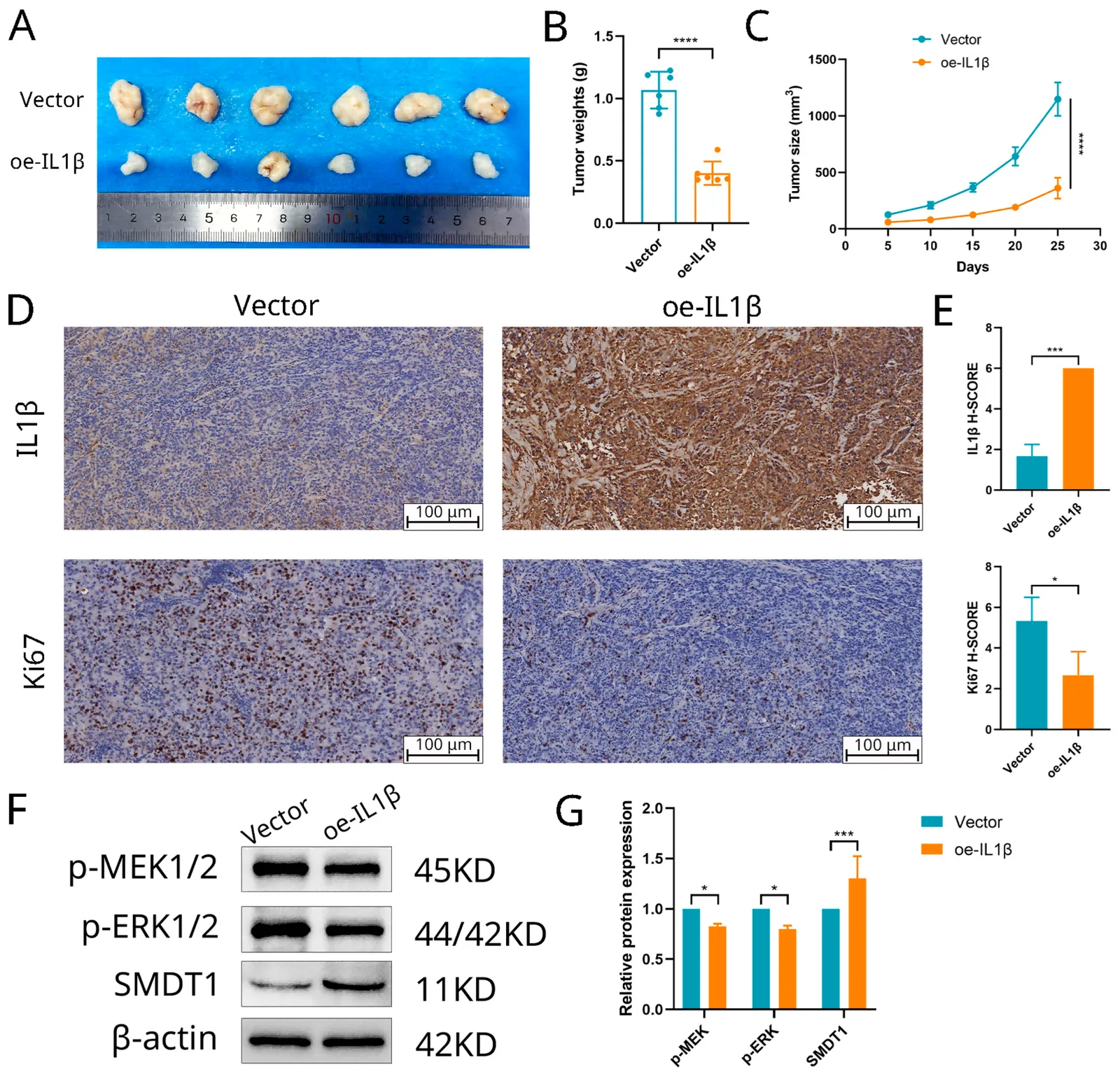
*IL1β* gene overexpression inhibits tumor growth in vivo (mean ± SD, n = 6). (**A**) Representative images of xenograft tumors. (**B**) Weight measurement of xenograft tumors. (**C**) Volume curve of xenograft tumors. (**D**) IHC staining of IL1β and Ki67 in xenograft tumor tissues. (**E**) Comparison of H-SCORE for IL1β and Ki67 protein in xenograft tumor tissues. (**F**,**G**) WB analysis of SMDT1, p-MEK, and p-ERK protein expression in xenograft tumor tissues. * indicates *p* < 0.05; *** indicates *p* < 0.001; **** indicates *p* < 0.0001. The whole blots (uncropped blots) are shown in the Supplementary Files.

To further validate IL1β’s tumor-suppressive effect in vivo, we established a nude mouse xenograft model via the Vector group and the oe-*IL1β* group cells. Tumors from the oe-*IL1β* group showed significantly reduced weight and volume compared with the Vector group, indicating that the overexpression of *IL1β* gene suppresses tumor growth in vivo (Figure 9A–C). Complete data are provided in Supplementary Tables S6 and S7. IHC staining of xenograft tissues revealed markedly increased IL1β expression and markedly reduced Ki67 expression in tumors from the oe-*IL1β* group relative to the Vector group (Figure 9D,E). WB analysis of xenograft tumor tissues also revealed that IL1β overexpression increased SMDT1 levels and decreased p-MEK and p-ERK levels (Figure 9F,G), consistent with the in vitro findings. These results suggest that IL1β suppresses PTC tumors partly via the SMDT1-MAPK signaling pathway.

## 4. Discussion

This study systematically evaluated the expression patterns, biological roles, and molecular mechanisms of the inflammatory cytokine IL1β in PTC. We identify the IL1β-SMDT1-MAPK signaling axis as a key pathway linking inflammation, mitochondrial regulation, and malignant progression in PTC. IL1β was markedly downregulated in PTC, and its expression correlated inversely with the number of lymph node metastases. Functionally, IL1β strongly inhibited PTC cell proliferation, colony formation, migration, invasion, and EMT, promoted apoptosis, and suppressed xenograft tumor growth in vivo. Mechanistically, as shown in Supplementary Figure S5, IL1β exerts a tumor-suppressive effect in PTC by upregulating the downstream mediator SMDT1 and by attenuating MAPK pathway activity.

IL1β is a canonical proinflammatory cytokine of the IL-1 family that participates in diverse physiological processes. Since IL1β is upregulated in various diseases, it is often implicated in the progression of inflammation-associated tumors. SPP1+ macrophages have been reported to promote head and neck squamous cell carcinoma progression by secreting IL1β [31]. Similarly, higher plasma IL1β levels in non-smokers have been associated with more rapid declines in lung function [32]. Mounting evidence indicates that IL1β’s biological effects are highly context-dependent, shaped by tumor genetic background, cell origin, receptor expression, and the immune microenvironment [13]. Our analyses showed markedly reduced IL1β expression in PTC, and this low expression correlated significantly with increased numbers of lymph node metastases. These findings suggest that IL1β deficiency may contribute to local invasion and regional metastasis in PTC. Thus, IL1β should not be viewed as a cytokine that universally promotes tumorigenesis. Rather, its role depends on the dominant signaling networks within each tumor type.

Prior studies have shown that the IL-1 family critically shapes the tumor immune microenvironment [33]. Our immune infiltration analysis indicates that IL1β expression correlates positively and significantly with infiltration by multiple immune cell types, implying that immune cells substantially contribute to the intratumoral IL1β pool. Therefore, the current study primarily demonstrates that tumor cell-derived IL1β is sufficient to suppress malignant phenotypes of PTC cells, but does not exclude important contributions from immune-derived IL1β within the tumor microenvironment. However, our findings were generated based on computational estimation from public databases and should therefore be interpreted cautiously. Since IL1β can be produced by both tumor cells and infiltrating immune cells, the specific cellular sources of IL1β and its spatial relationship with immune populations remain unclear. Future studies using multiplex immunofluorescence, spatial transcriptomics, or single-cell RNA sequencing will be necessary to elucidate the cellular origin of IL1β and how it contributes to immune regulation within the PTC microenvironment.

At the functional level, two experimental strategies were used to evaluate IL1β function: extracellular IL1β stimulation using recombinant cytokine treatment, and intracellular IL1β manipulation through genetic overexpression. Although these approaches cannot completely exclude differences between intracellular and extracellular IL1β functions, the consistent inhibitory effects observed in both models suggest that IL1β signaling negatively regulates PTC progression. In a nude mouse xenograft model, IL1β likewise exhibited an anticancer effect in vivo. Notably, prior studies reported that IL1β suppresses proliferation in some PTC cell lines (TPC-1 and NPA) and an FTC cell line (CGTH W-1) [34,35,36,37], a finding that aligns with our results. Similarly, other work showed that the proinflammatory cytokine IL1β reduces viability and metastasis of ovarian cancer cells via the MAPK/MMP12 pathway [14]. By contrast, in cancers such as breast cancer, colorectal cancer, and glioma, IL1β has been linked to increased invasiveness, enhanced metastasis, and resistance to immunotherapy [9,38,39,40]. Our study further shows that *IL1β* gene overexpression induces apoptosis in PTC cells, inhibits the EMT program, and suppresses MMPs expression, indicating the activation of a mitochondria-centered apoptotic pathway and providing a cellular basis for reduced metastatic behavior. However, these results contrast with reports in other cancers. Some studies in breast cancer found that IL1β promotes EMT and bone metastasis via the NF-κB pathway [40,41,42]. Such discrepancies underscore that IL1β’s effects vary by tumor type and depend on factors including genetic background, receptor expression, and the immune microenvironment [9,13].

In this study, IL1β exerted a pronounced inhibitory effect on two BRAF-mutant PTC cell lines (BCPAP and KTC-1), indicating particular relevance in PTC subtypes that depend heavily on the MAPK pathway. Contrary to the canonical pattern in which IL1β acts upstream to activate the MAPK pathway in many diseases [9,43,44], our RNA-seq and protein-level analyses consistently demonstrated that IL1β markedly reduced phosphorylation of MEK1/2 and ERK1/2 in PTC cells while leaving their total protein levels essentially unchanged. This finding implies that IL1β remodels MAPK signaling chiefly by inhibiting kinase activity rather than by down-regulating expression. Importantly, pharmacological activation of MAPK signaling using C16-PAF partially restored the malignant phenotypes suppressed by IL1β, providing functional evidence that MAPK inhibition is a critical mediator of IL1β-induced tumor suppression. Thus, we propose that IL1β functions as a negative regulator within the BRAF-MAPK regulatory network, accounting for its tumor-suppressive effects in PTC at the level of signal transduction.

Mitochondrial dysfunction is vital in tumor progression [45,46]. SMDT1, a component of the mitochondrial calcium uniporter (MCU) complex, regulates mitochondrial Ca^2+^ homeostasis, cell metabolism, and apoptosis [47]. Although SMDT1’s impact on tumor cell behavior is known [23,30], its upstream regulatory mechanism is unclear. In this study, we confirmed an association between IL1β and SMDT1 in PTC cells by proteomic analysis and Co-IP. Additional in vitro experiments showed that *IL1β* gene overexpression markedly increased *SMDT1* gene expression. *SMDT1* gene knockdown partially reversed the inhibitory effects of *IL1β* gene overexpression on the malignant phenotype of PTC cells. Importantly, unlike our previous study that characterized the biological function of SMDT1 in THCA cells, the current study identifies SMDT1 as a mediator connecting inflammatory regulation and oncogenic signaling. These findings provide a new perspective on SMDT1 biology by demonstrating that its expression and function can be modulated by upstream inflammatory cues. Thus, the IL1β-SMDT1-MAPK signaling axis offers a mechanistic hypothesis for how inflammatory factors modulate tumor metabolism and proliferation through mitochondrial signaling.

This study extends prior work by broadening the biological understanding of inflammation-related molecules in THCA. Given that IL1β is a multifunctional cytokine, it may regulate PTC progression through mechanisms beyond those described here. For example, IL1β overexpression might alter the secretion profile of inflammatory cytokines and thereby change the tumor microenvironment. IL1β might also exert tumor-suppressive effects in PTC via apoptosis-related pathways, metabolic regulation, or other microenvironmental processes. Thus, the IL1β-SMDT1-MAPK signaling axis identified in this study should be considered an important regulatory pathway, not the sole mechanism underlying IL1β-mediated tumor suppression. Future work will employ multiplex cytokine detection and single-cell transcriptomics for more in-depth investigation.

However, this study has several limitations. First, clinical samples were collected from a single center, so potential selection bias and regional differences cannot be completely excluded. Although KM survival analysis indicated that IL1β and SMDT1 may have prognostic value, those results derive from public databases that lack detailed clinical covariates and recurrence data. Therefore, future independent cohort studies with larger-scale, multicenter, and longer follow-up are required. Second, in vitro work relied mainly on BRAF-mutated PTC cell lines, so generalizability must be confirmed in additional PTC models with diverse genetic backgrounds, in patient-derived organoids, or in primary tumor cells. Third, although Co-IP experiments indicated an association between IL1β and SMDT1-related protein complexes, these results do not establish whether IL1β directly binds to SMDT1. Therefore, future studies using GST pull-down, protein domain mapping, and structural analysis will be necessary to determine whether a direct interaction exists. In addition, although our study demonstrated that IL1β positively regulates *SMDT1* gene expression at both mRNA and protein levels, the precise molecular mechanism remains unclear. Future studies involving SMDT1 promoter analysis, transcription factor screening, luciferase reporter assays, and ChIP-qPCR will be necessary to elucidate the detailed regulatory mechanism. Finally, although the subcutaneous tumorigenicity model in nude mice assesses the intrinsic growth capacity of PTC cells, it does not evaluate metastatic potential. Future studies could examine IL1β effects on PTC metastasis and the immune microenvironment by employing an orthotopic THCA model and a tail-vein injection metastasis model in mice.

## 5. Conclusions

Collectively, our findings identify the IL1β-SMDT1-MAPK signaling axis as a critical regulatory pathway involved in PTC progression and provide new insights into the context-dependent role of inflammatory factors in THCA.

## Data Availability

The original RNA-seq data presented in the study are openly available in the GEO database (https://www.ncbi.nlm.nih.gov/geo/; accessed on 1 June 2026), with the accession number GSE326249. The original mass spectrometry proteomics data presented in the study are openly available in the ProteomeXchange Consortium (https://proteomecentral.proteomexchange.org; accessed on 1 June 2026) via the iProX partner repository, with the accession number PXD076546.

## Supplementary Materials

The following supporting information can be downloaded at: https://www.mdpi.com/article/10.3390/cancers18182918/s1. Supplementary Figure S1: Correlations of IL1β expression with immune infiltration level in THCA. Supplementary Figure S2: Pharmacological activation of MAPK signaling partially rescues IL1β-mediated suppression of malignant phenotypes in BCPAP cells. (A,B) The effects of the MAPK activator C16-PAF on the expression levels of MAPK pathway-related proteins in BCPAP cells were detected by WB (mean ± SD, n = 3). (C,D) The effects of C16-PAF on IL1β-mediated inhibition of the proliferation, migration, and invasion abilities of BCPAP cells (mean ± SD, n = 3). ns indicates *p* > 0.05; * indicates *p* < 0.05; ** indicates *p* < 0.01; *** indicates *p* < 0.001; **** indicates *p* < 0.0001. Supplementary Figure S3: Expression analysis and survival analysis of the *SMDT1* gene in THCA. (A) SMDT1 expression in THCA. (B) KM survival curves for DFS associated with SMDT1 expression levels. **** indicates *p* < 0.0001. Supplementary Figure S4: Construction and validation of the SMDT1 gene-modified cell model. (A–C) The transfection efficiency of siRNA was detected by qRT-PCR and WB (mean ± SD, n = 4). (D–F) *SMDT1* gene was knocked down in the oe-*IL1β* group cells, and the expression of *SMDT1* gene was detected by qRT-PCR and WB (mean ± SD, n = 3). ** indicates *p* < 0.01; *** indicates *p* < 0.001; **** indicates *p* < 0.0001. Supplementary Figure S5: Schematic graphical model of the proposed IL1β-SMDT1-MAPK signaling axis in PTC. Supplementary Table S1: The primer sequences. Supplementary Table S2: Information about antibodies. Supplementary Table S3: The sequence information of siRNAs. Supplementary Table S4: The pan-cancer expression analysis of *IL1β gene*. Supplementary Table S5: Association between *IL1β* gene expression levels and clinical characteristics in PTC tissues. Supplementary Table S6: Volume measurement of xenograft tumors. Supplementary Table S7: Weight measurement of xenograft tumors. Supplementary original images: Original images from the Western blotting, colony formation assay, wound-healing assay and transwell assays.

## Abbreviations

The following abbreviations are used in this manuscript:

AJCC	American Joint Committee on Cancer
ANOVA	Analysis of Variance
ATP	Adenosine Triphosphate
BCA	Bicinchoninic Acid
CCK-8	Cell Counting Kit-8
cDNA	Complementary DNA
Co-IP	Co-immunoprecipitation
CO_2_	Carbon Dioxide
CYT	Cytolytic Score
DAB	3,3′-Diaminobenzidine
DEGs	Differentially Expressed Genes
DIA	Data-Independent Acquisition
DNA	Deoxyribonucleic Acid
EDTA	Ethylenediaminetetraacetic Acid
EMT	Epithelial–Mesenchymal Transition
ERK1/2	Extracellular Signal-Regulated Kinase 1/2
FBS	Fetal Bovine Serum
FC	Fold Change
FITC	Fluorescein Isothiocyanate
FTC	Follicular Thyroid Carcinoma
GEO	Gene Expression Omnibus
GEPIA3	Gene Expression Profiling Interactive Analysis 3
GO	Gene Ontology
GSEA	Gene Set Enrichment Analysis
H-SCORE	Histochemical Score
IACUC	Institutional Animal Care and Use Committee
IC_50_	Half-maximal Inhibitory Concentration
IgG	Immunoglobulin G
IHC	Immunohistochemistry
IL-1RA	Interleukin-1 Receptor Antagonist
*IL1β*	Interleukin-1 beta
KEGG	Kyoto Encyclopedia of Genes and Genomes
MAPK	Mitogen-activated protein kinase
MCU	Mitochondrial Calcium Uniporter
MEK1/2	Mitogen-Activated Protein Kinase Kinase 1/2
MMPs	Matrix Metalloproteinases
MS	Mass Spectrometry
NF-κB	Nuclear Factor Kappa-B
PBS	Phosphate-Buffered Saline
PDAC	Pancreatic Ductal Adenocarcinoma
PI	Propidium Iodide
PVDF	Polyvinylidene Fluoride
PTC	Papillary Thyroid Carcinoma
qRT-PCR	Quantitative Real-Time Polymerase Chain Reaction
RIPA	Radioimmunoprecipitation Assay buffer
RNA	Ribonucleic Acid
RNA-seq	RNA sequencing
RPMI	Roswell Park Memorial Institute medium
SD	Standard Deviation
SDS-PAGE	Sodium Dodecyl Sulfate-Polyacrylamide Gel Electrophoresis
*SMDT1*	Single-pass membrane protein with aspartate-rich tail 1
siRNA	Small Interfering RNA
SPF	Specific pathogen-free
TCGA	The Cancer Genome Atlas
THCA	Thyroid Carcinoma
TIMER	Tumor Immune Estimation Resource
TNM	Tumor-Node-Metastasis
UICC	Union for International Cancer Control
WB	Western Blotting
