# Supplementary material for: IL1β Functions as a Tumor Suppressor in Papillary Thyroid Carcinoma via the SMDT1-MAPK Signaling Axis
Source: Cancers (Basel). 2026 Sep 9;18(18):2918. doi: 10.3390/cancers18182918 (PMC13604535; doi:10.3390/cancers18182918)

1 Figure 1H:

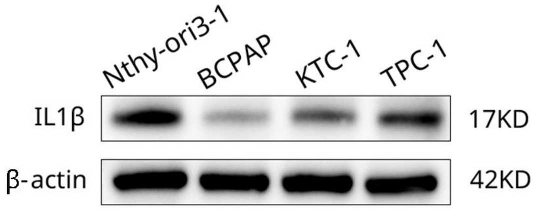

1.1 IL1β (17KD):

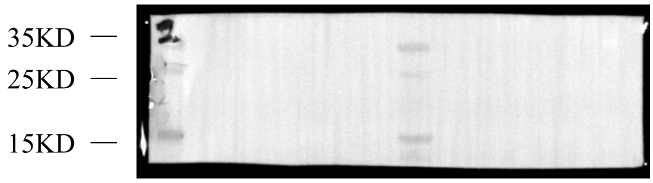

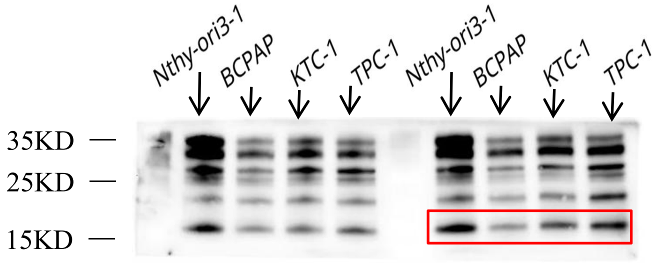

100: 48: 58: 66 **100: 39: 63: 81**

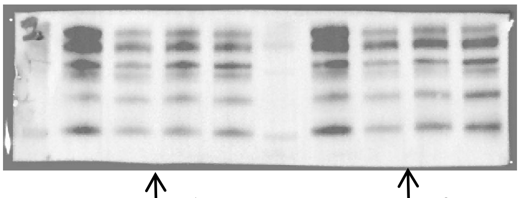

Repeat1 **Repeat2**

Repeat3

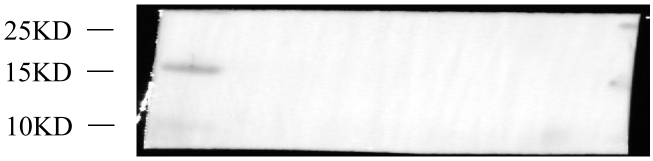

Repeat3

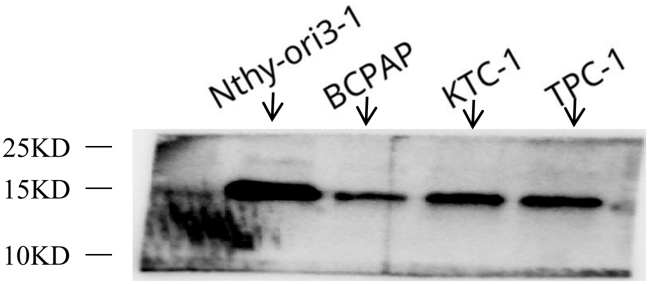

100 : 57 : 72 : 73

Repeat3

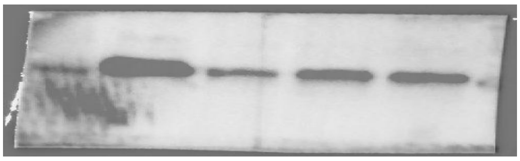

1.2 β-actin (42KD):

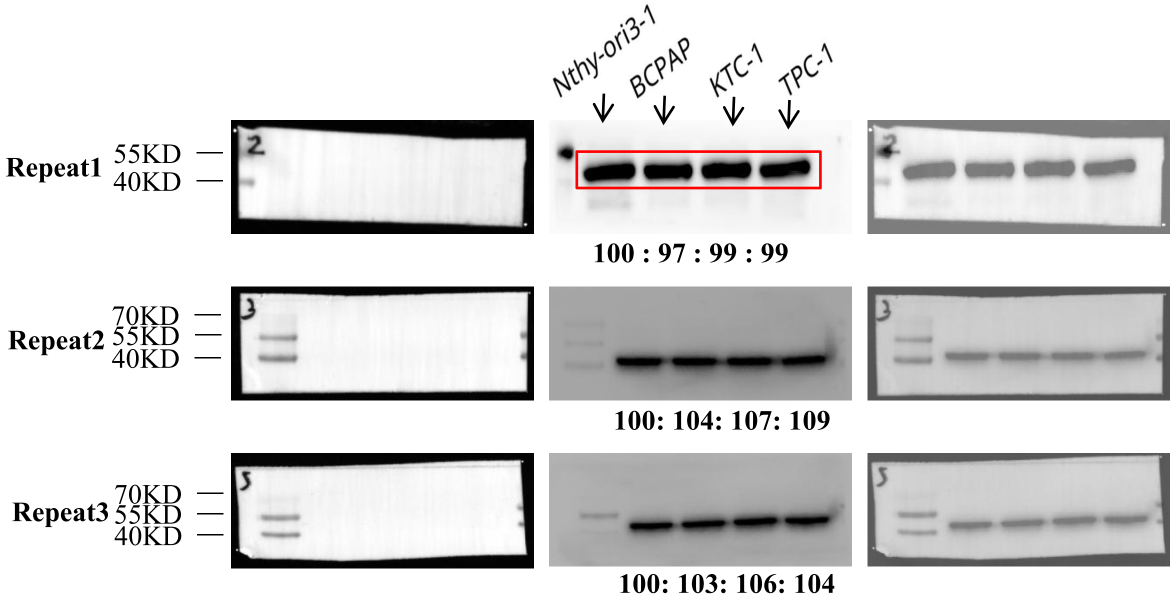

2 Figure 3B:

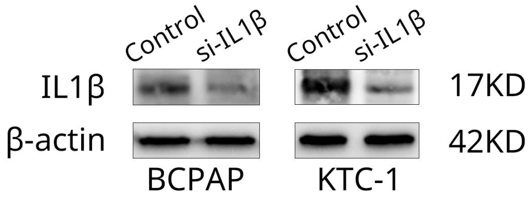

2.1 BCPAP

2.1.1 IL1β (17KD):

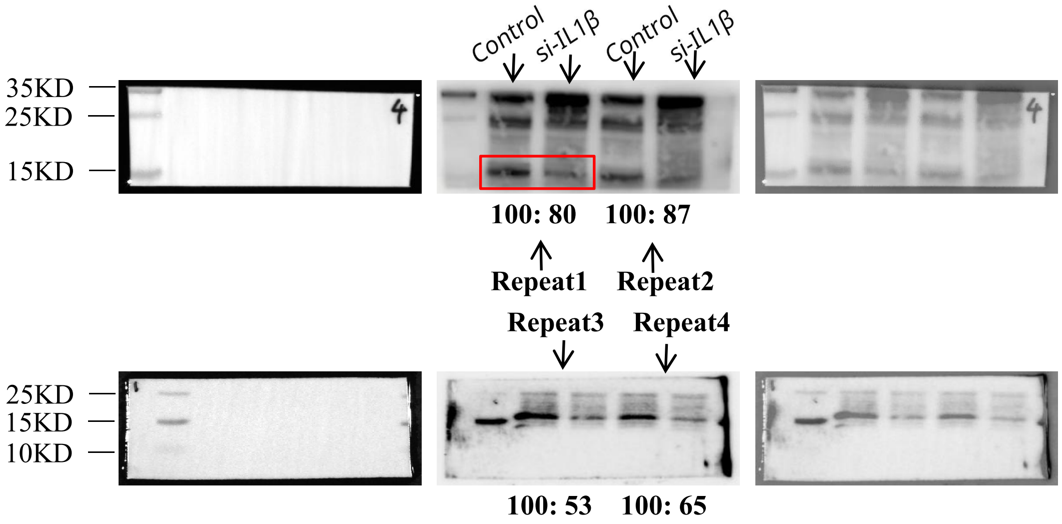

2.1.2 β-actin (42KD):

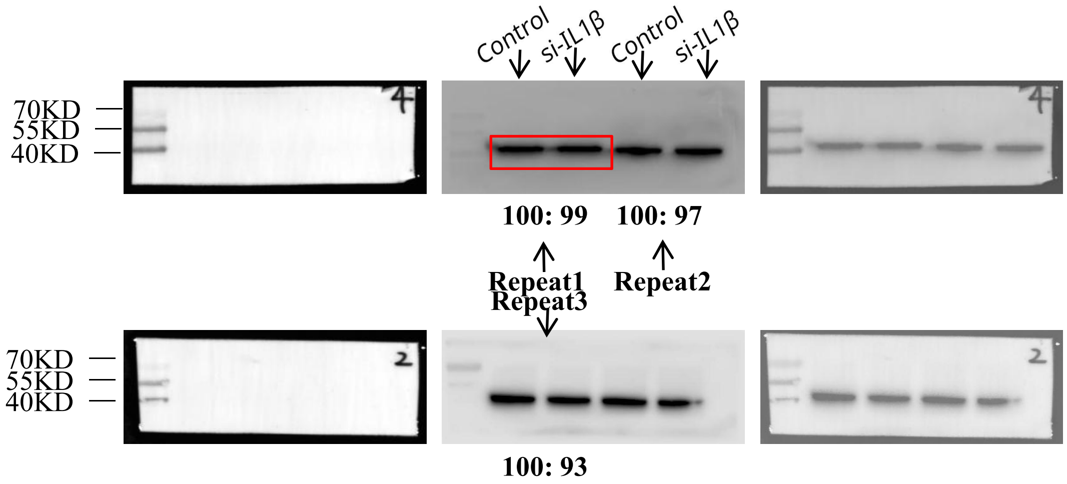

2.2 KTC-1

2.2.1 IL1β (17KD):

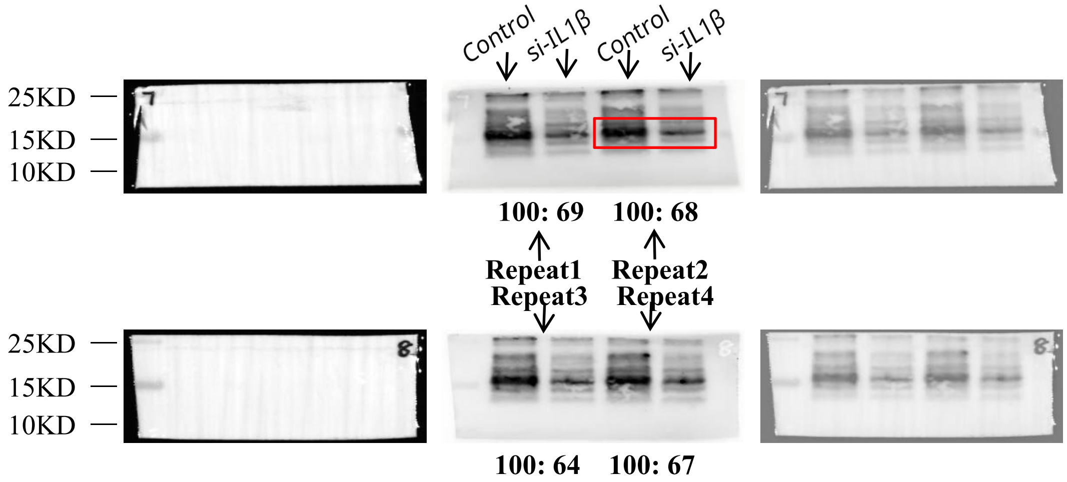

2.2.2 β-actin (42KD):

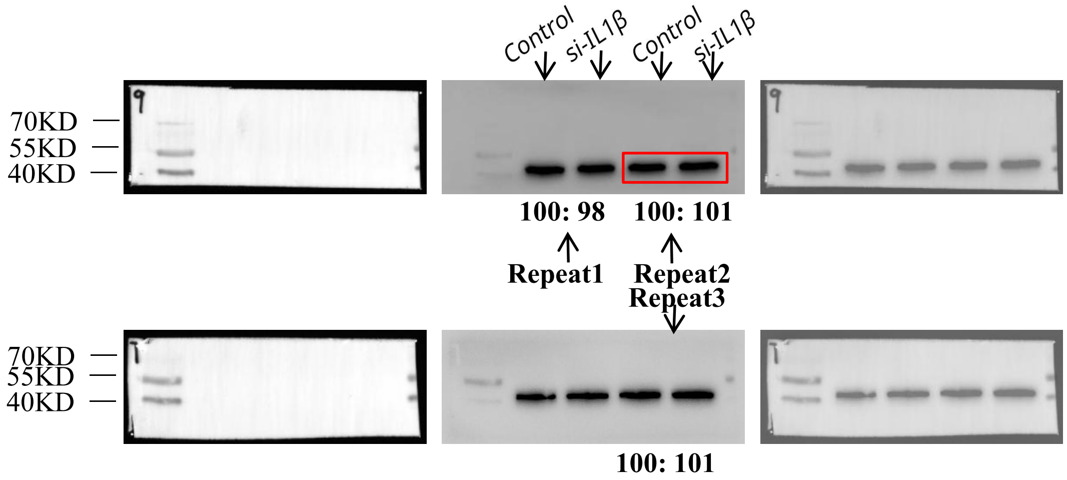

3 Figure 3E:

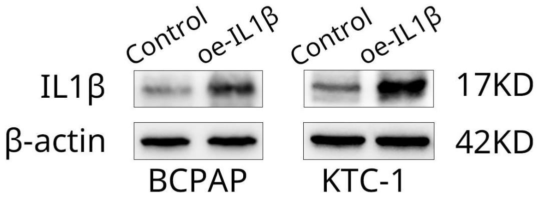

3.1 BCPAP

3.1.1 IL1β (17KD):

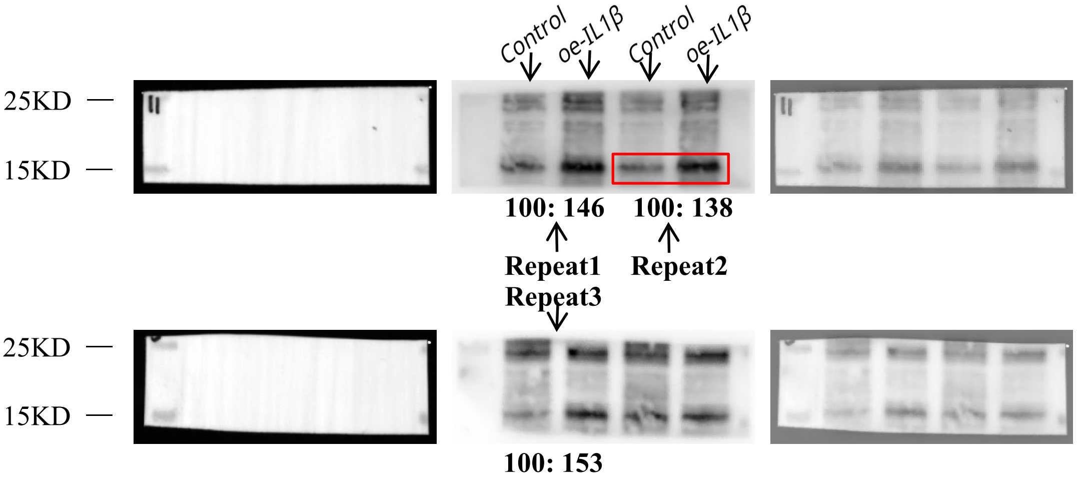

3.1.2 β-actin (42KD):

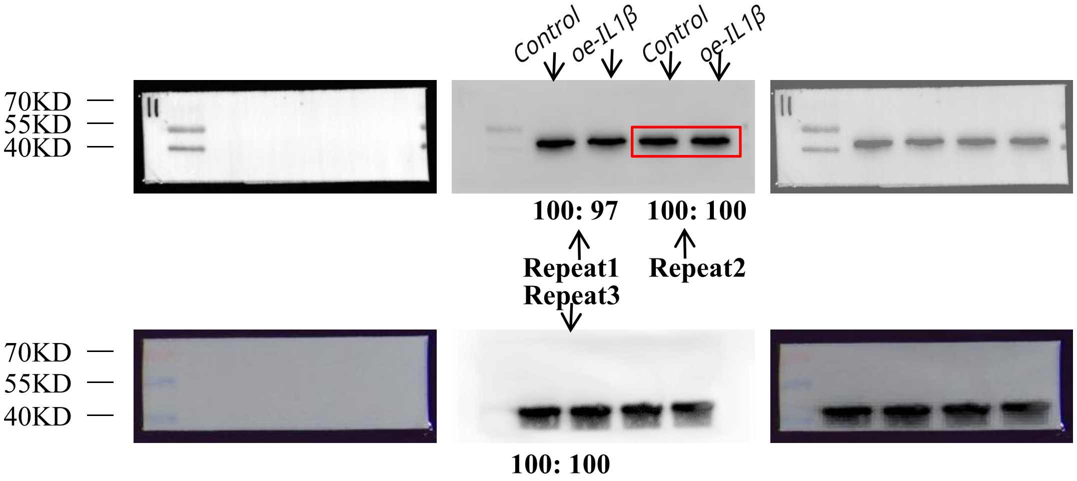

3.2 KTC-1

3.2.1 IL1β (17KD):

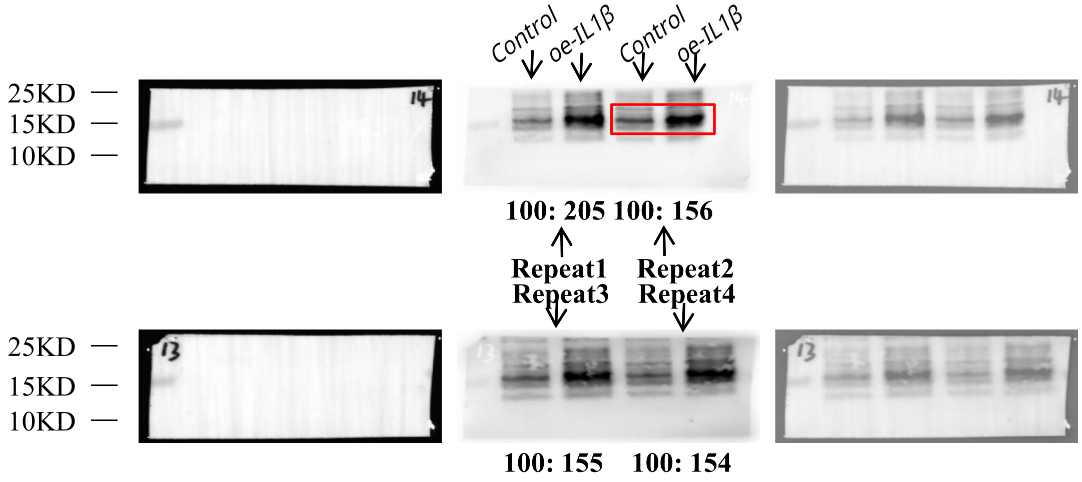

3.2.2 β-actin (42KD):

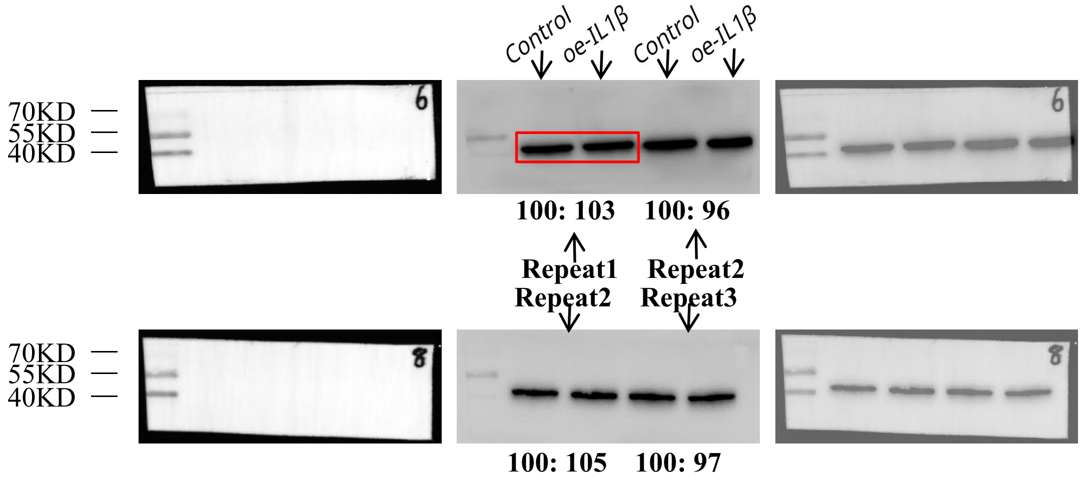

4 Figure 4C and 4D:

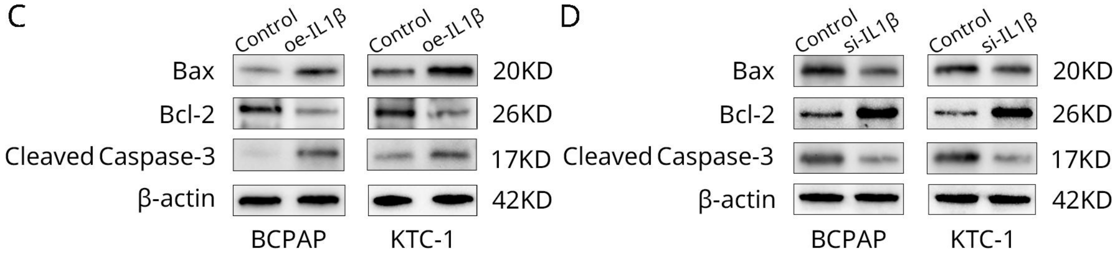

4.1 BCPAP

4.1.1 Bax (20KD):

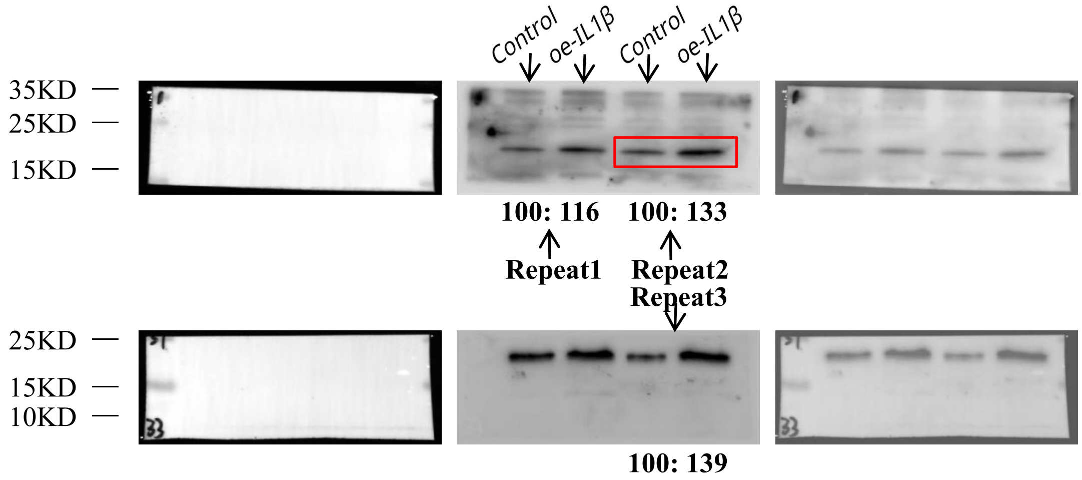

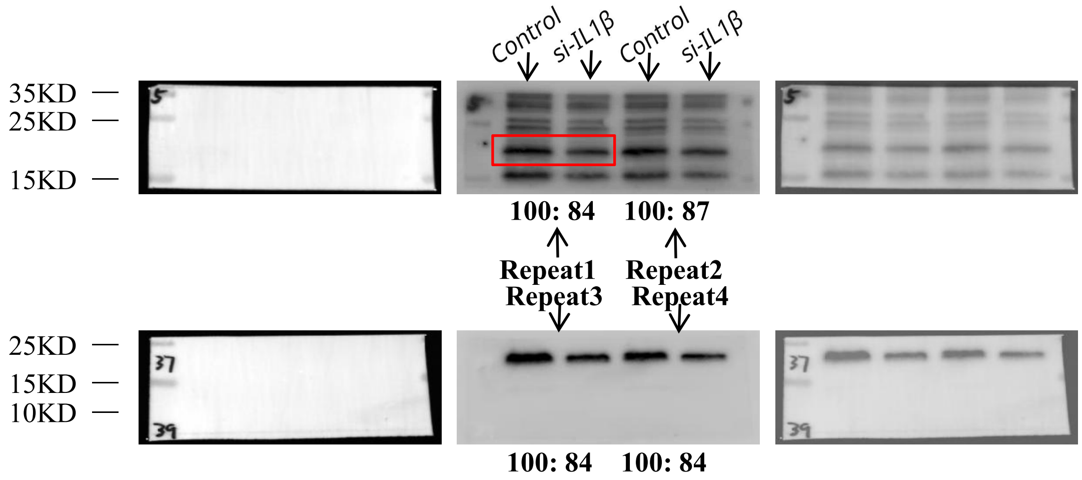

4.1.2 Bcl-2 (26KD):

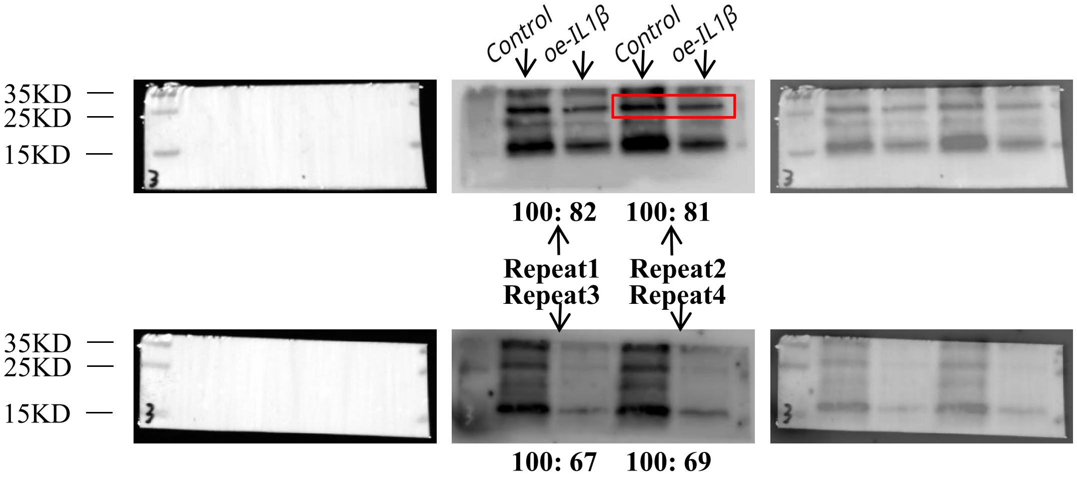

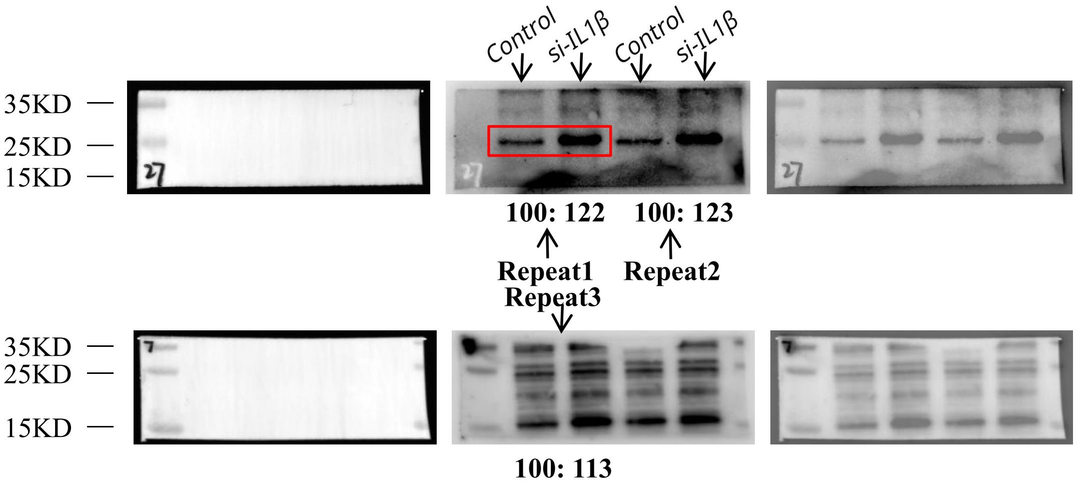

4.1.3 Cleaved Caspase-3 (17KD):

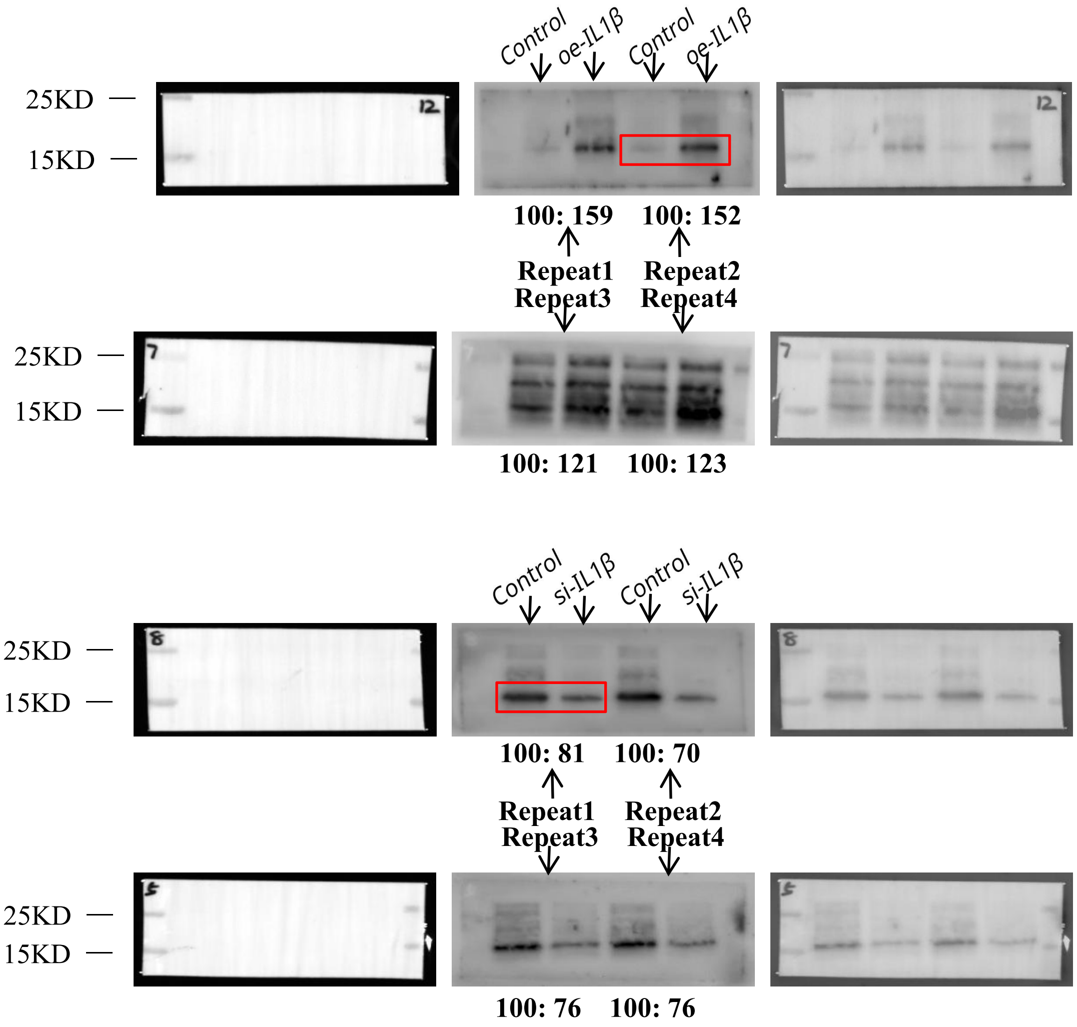

4.1.4 β-actin (42KD):

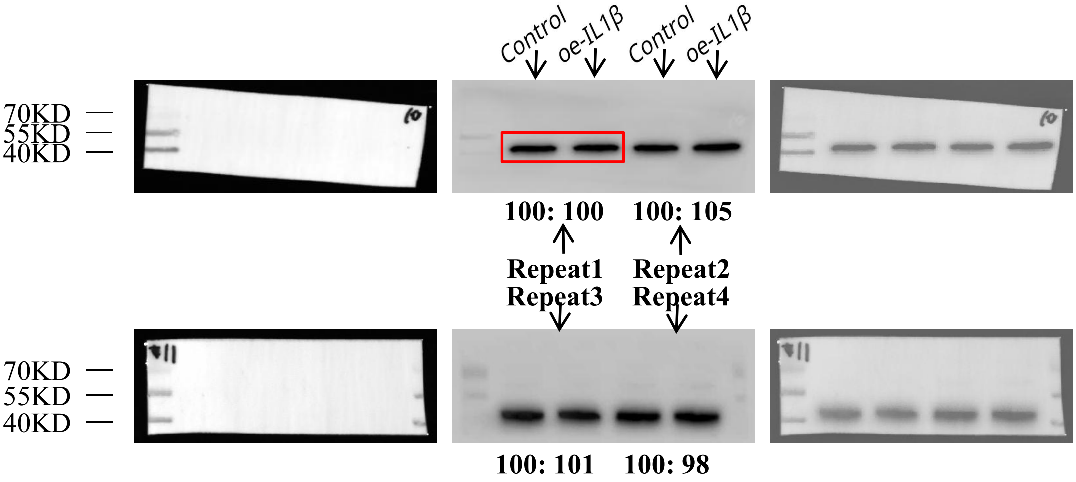

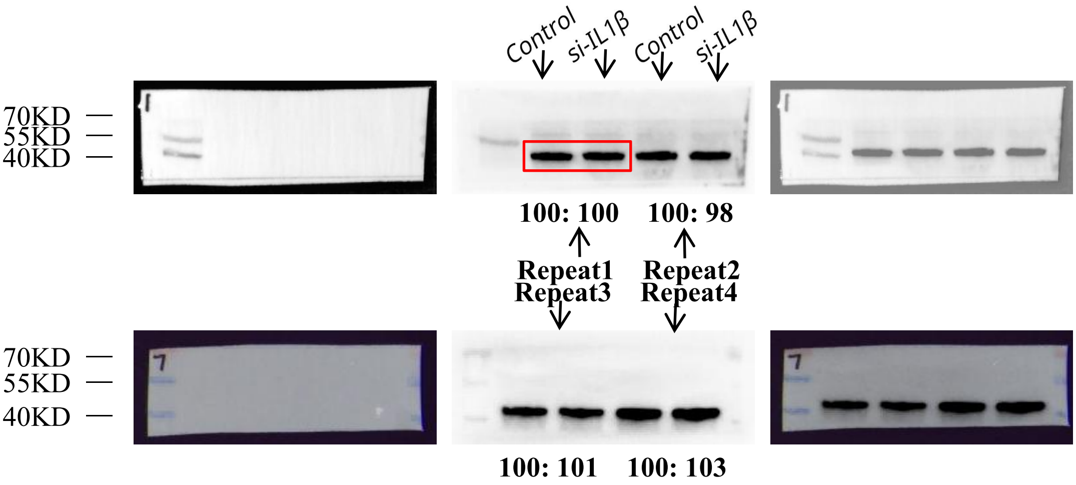

4.2 KTC-1

4.2.1 Bax (20KD):

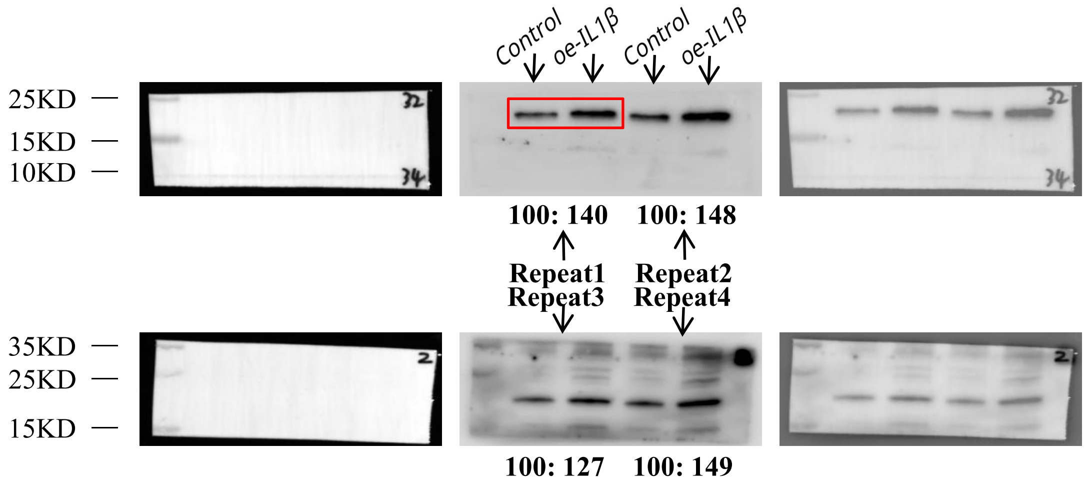

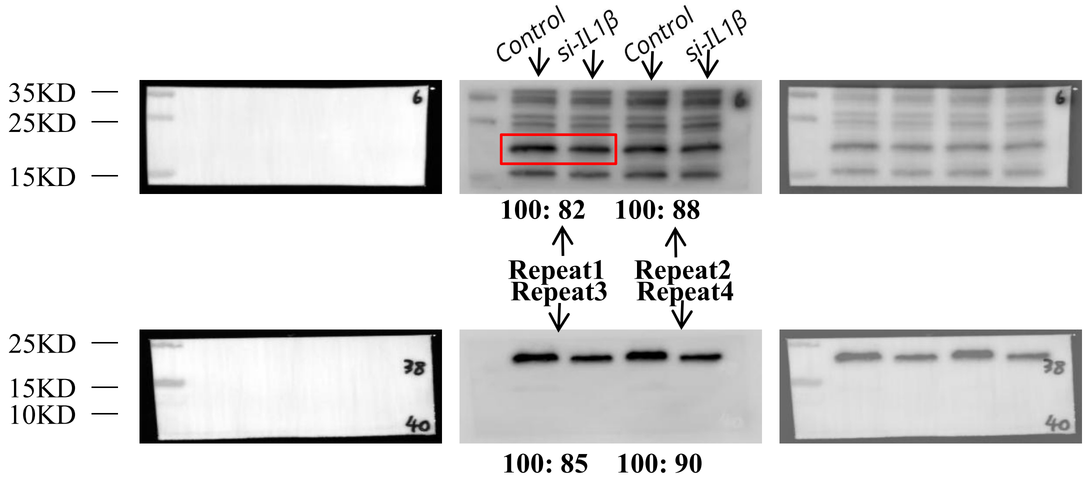

4.2.2 Bcl-2 (26KD):

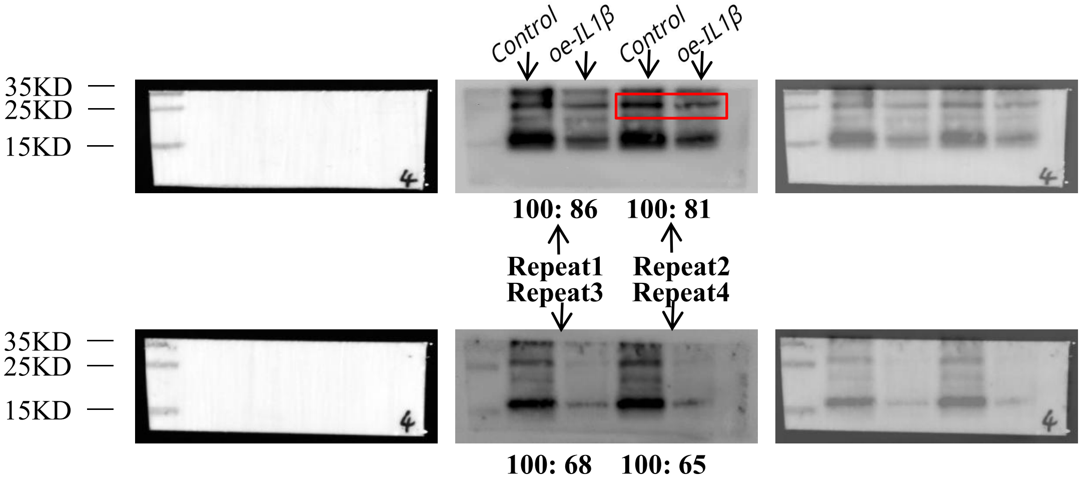

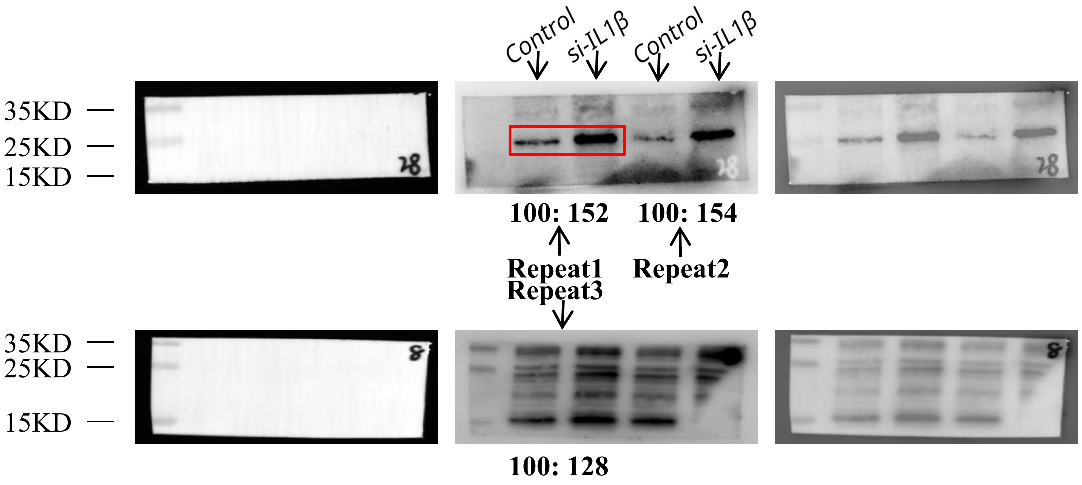

4.2.3 Cleaved Caspase-3 (17KD):

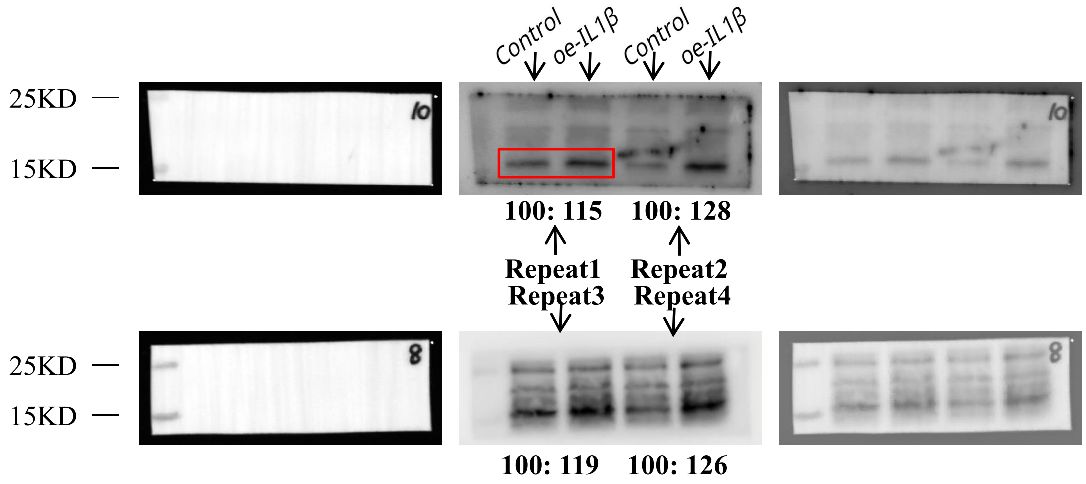

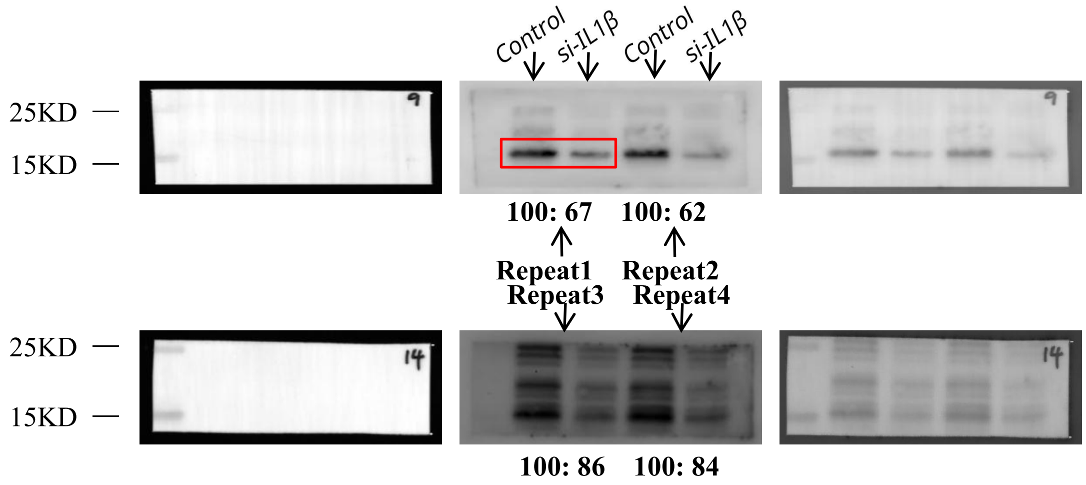

4.2.4 β-actin (42KD):

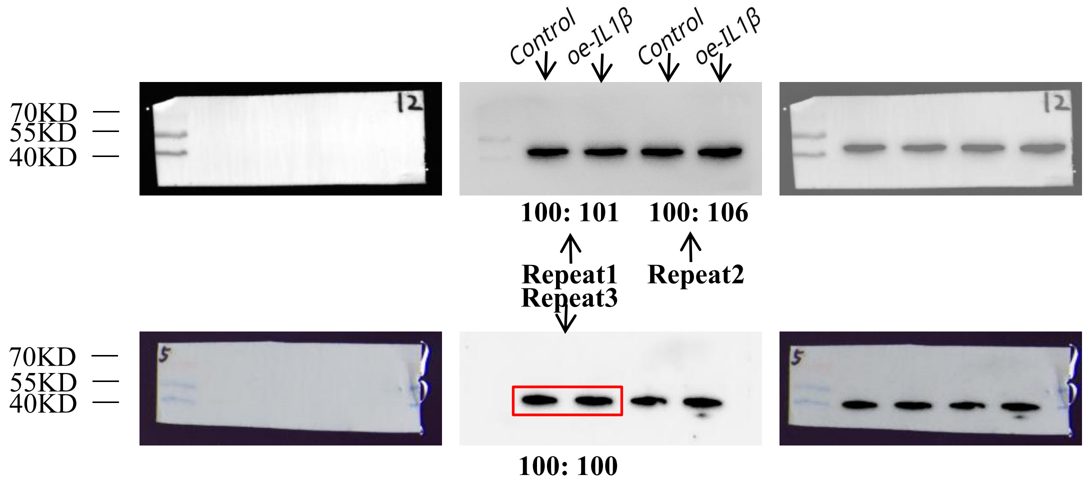

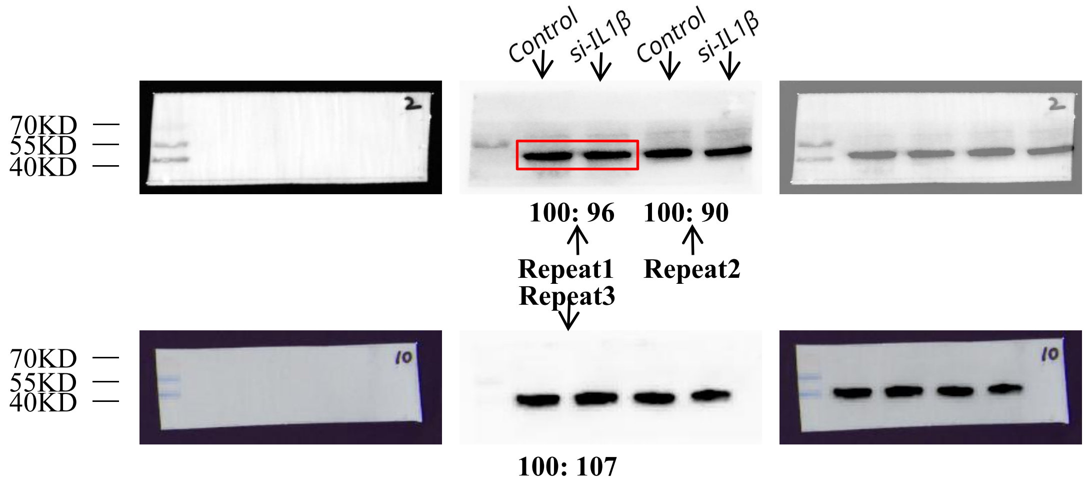

5 Figure 5E and 5F:

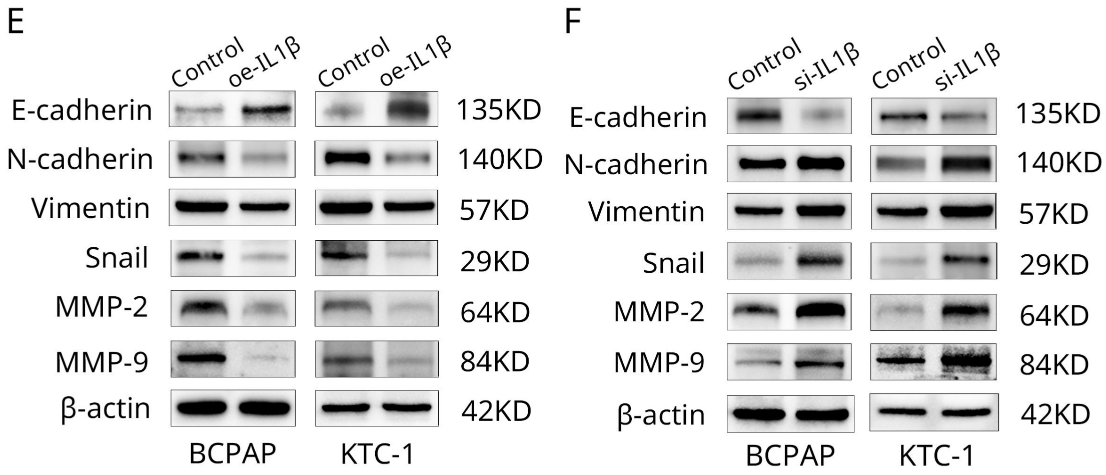

5.1 BCPAP

5.1.1 E-cadherin (135KD):

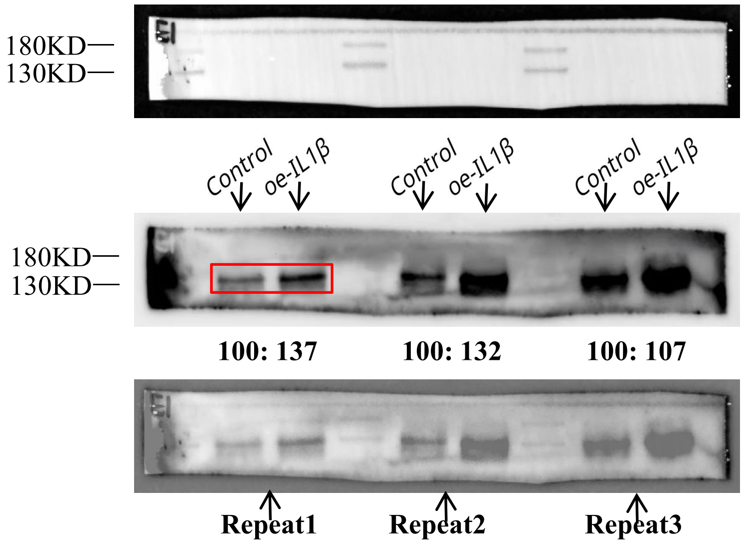

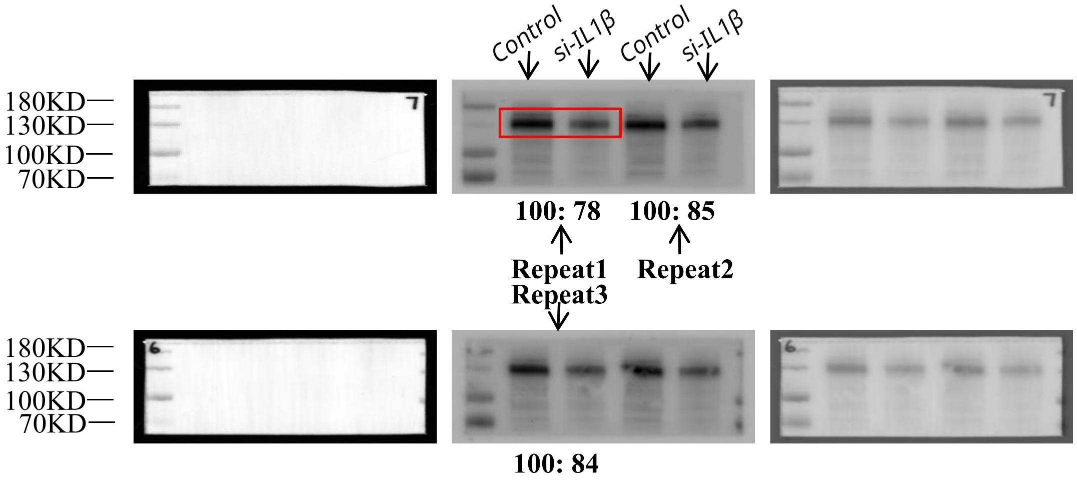

5.1.2 N-cadherin (140KD):

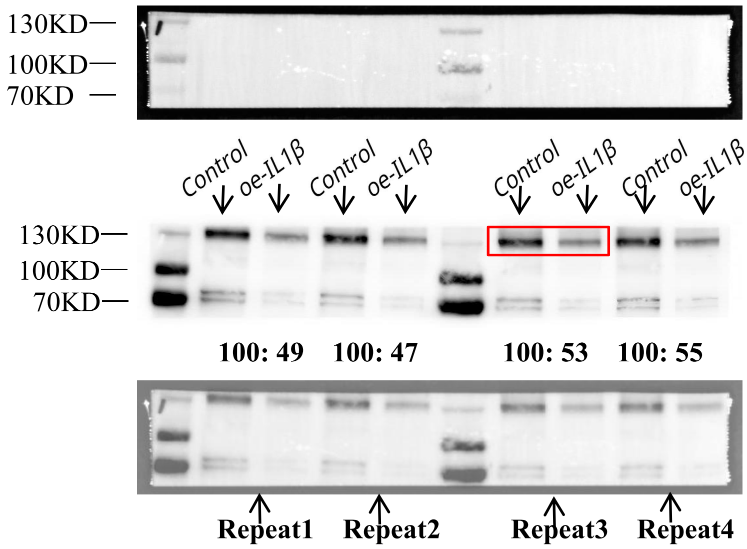

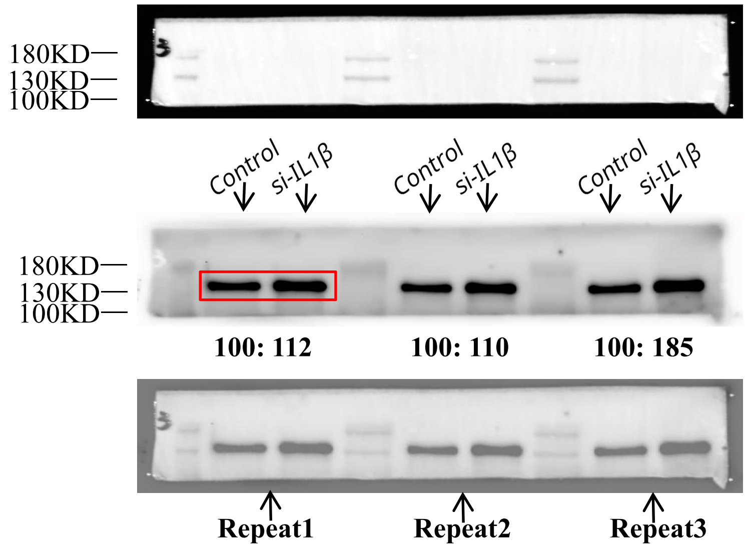

5.1.3 Vimentin (57KD):

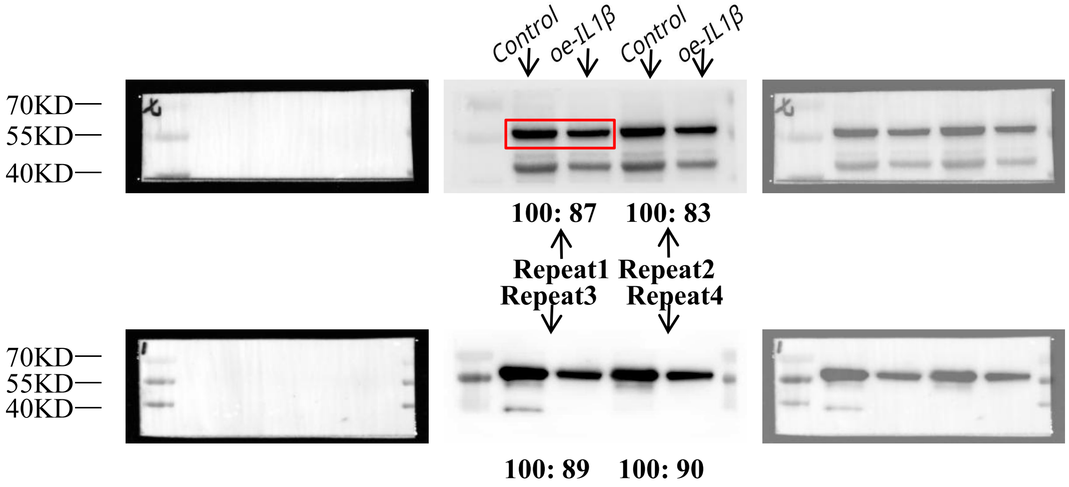

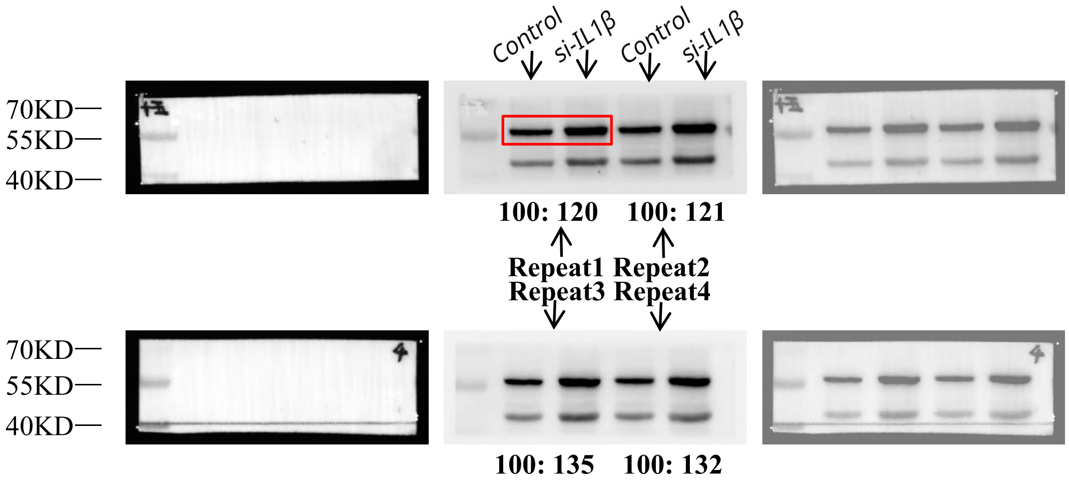

5.1.4 Snail (29KD):

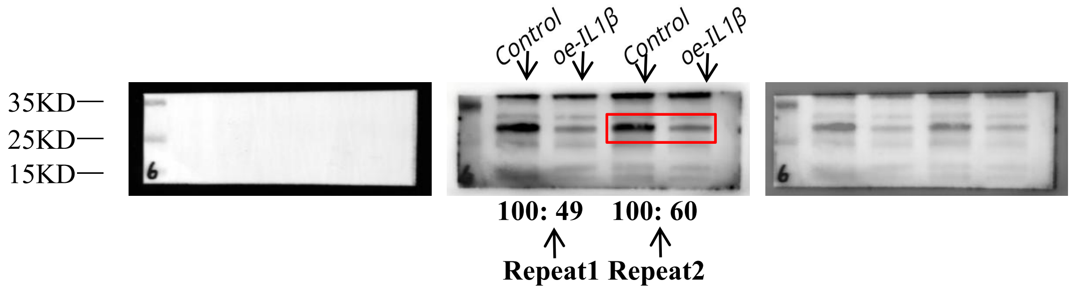

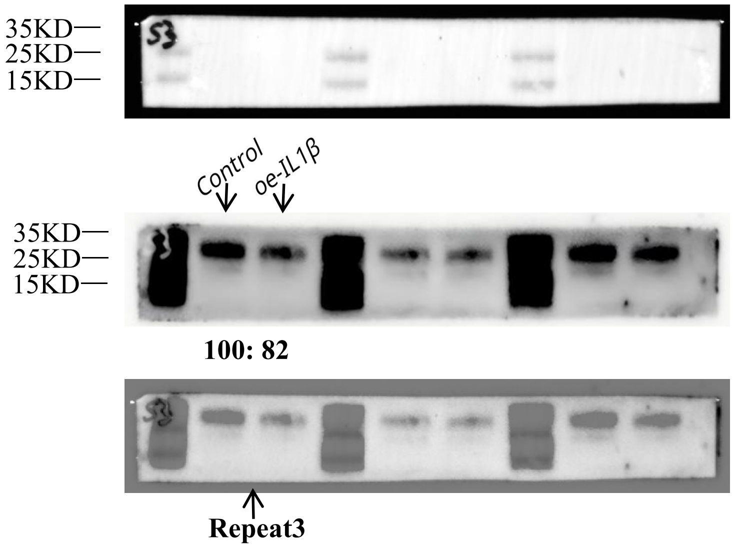

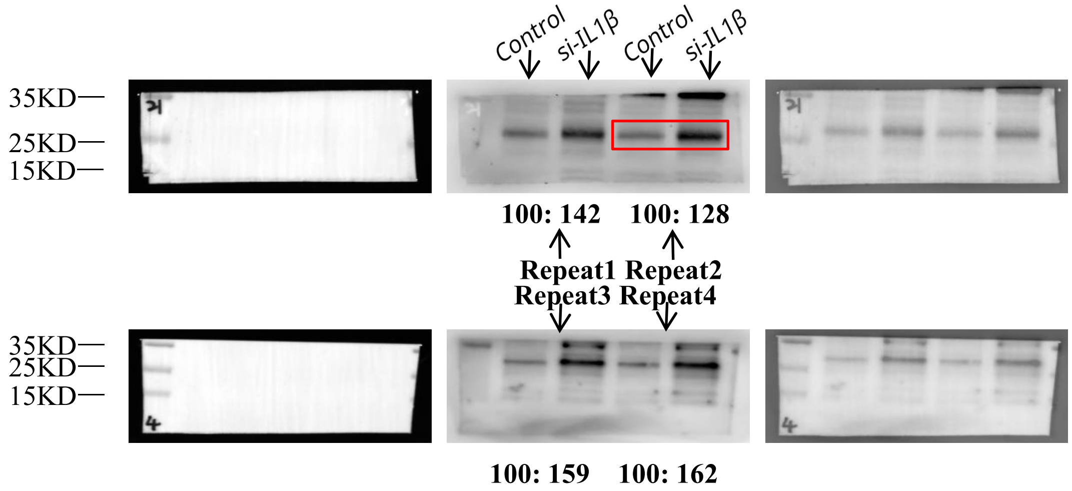

5.1.5 MMP-2 (64KD):

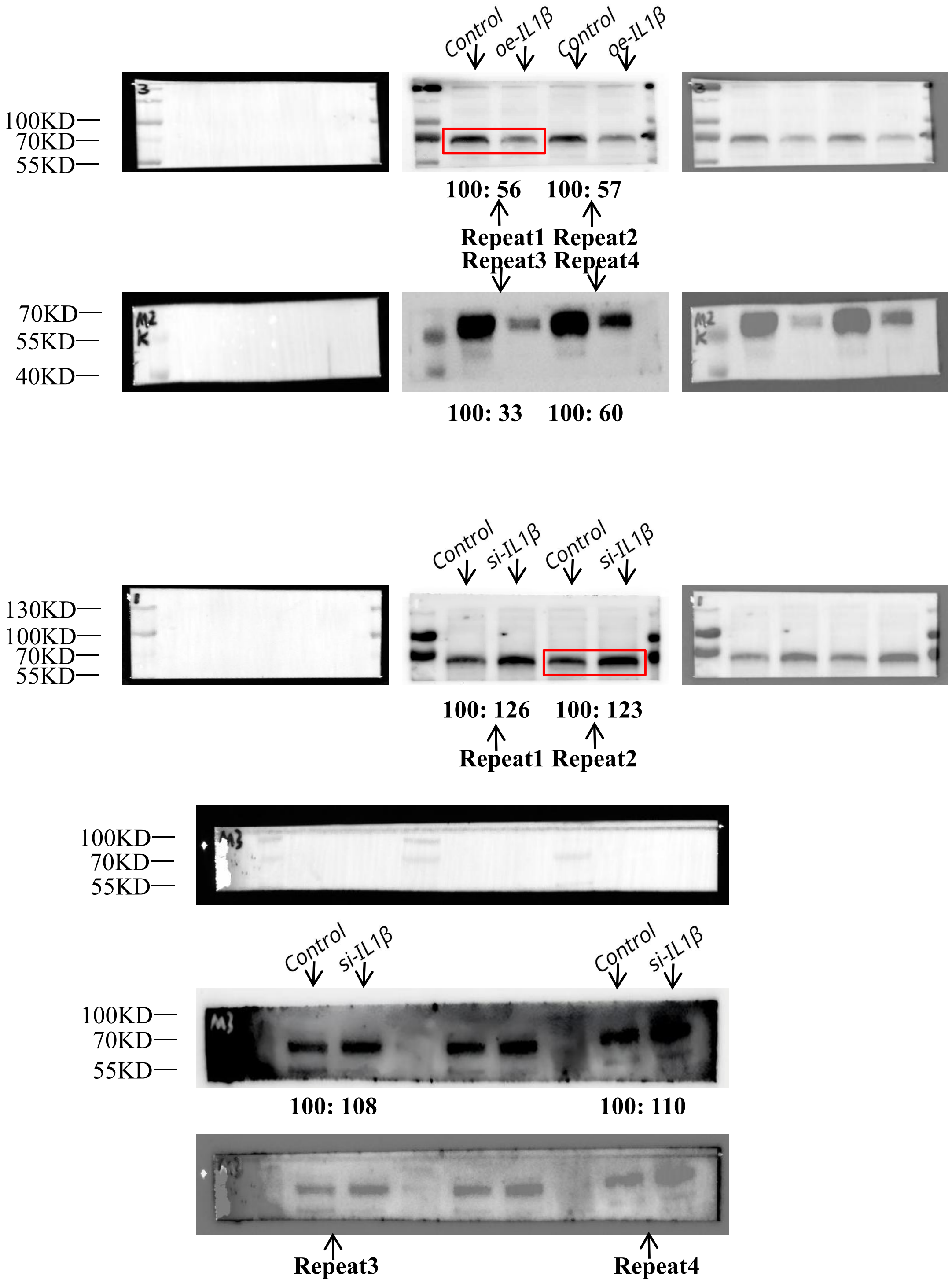

5.1.6 MMP-9 (84KD):

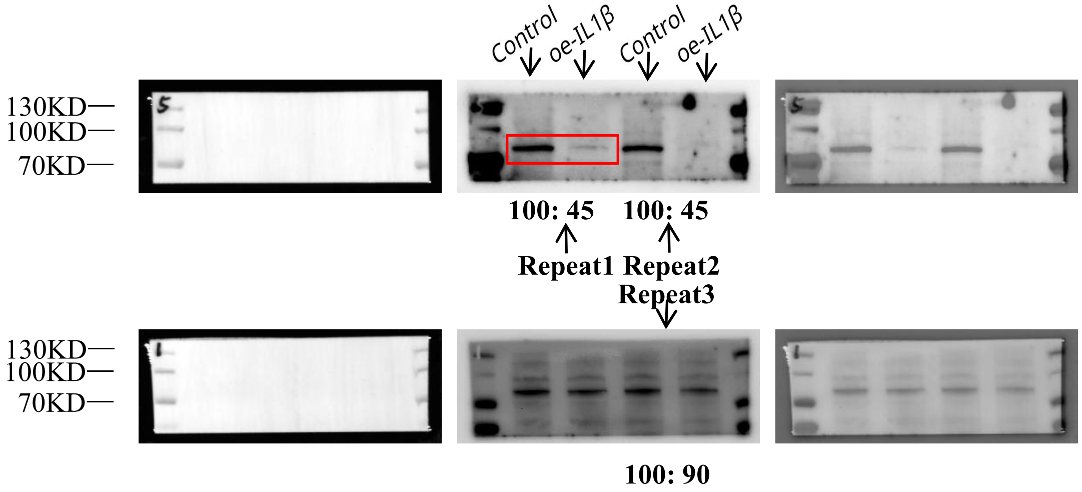

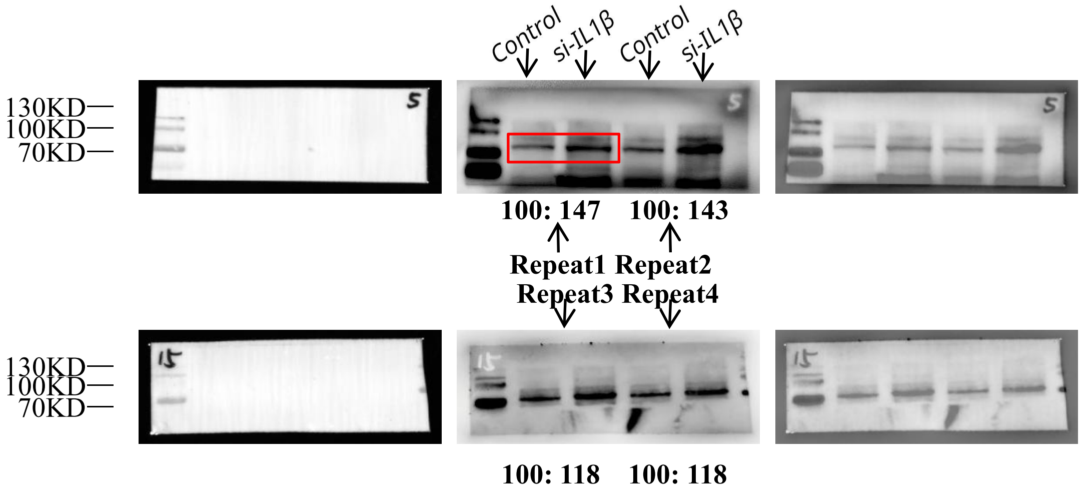

5.1.7 β-actin (42KD):

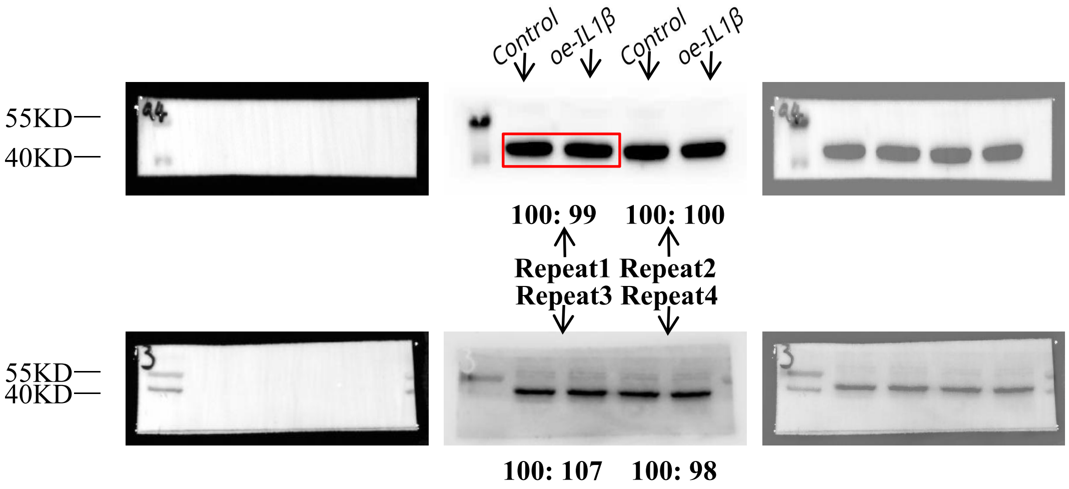

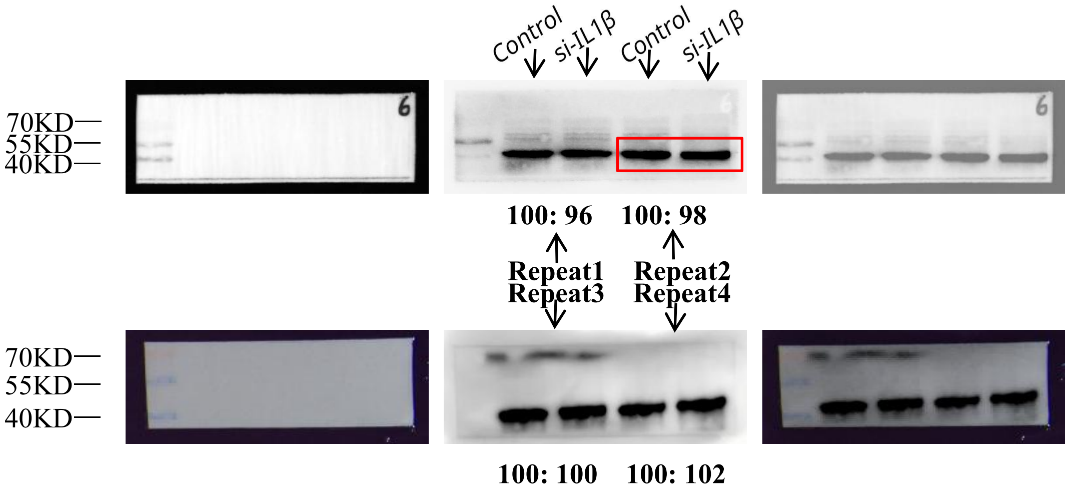

5.2 KTC-1

5.2.1 E-cadherin (135KD):

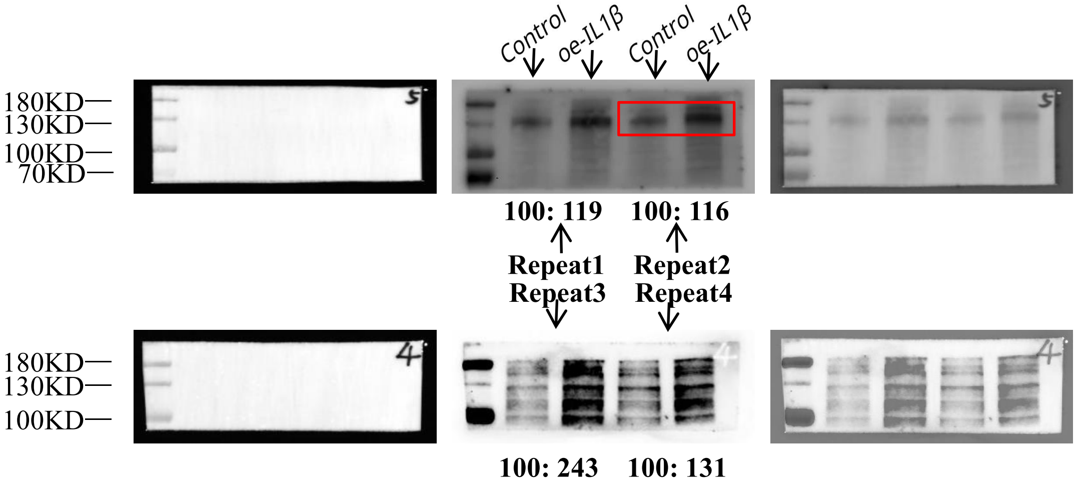

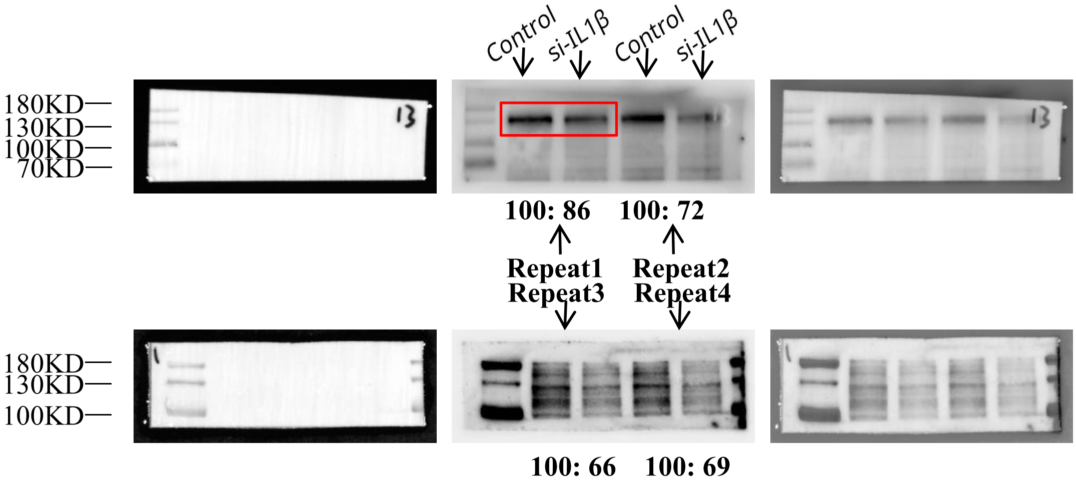

5.2.2 N-cadherin (140KD):

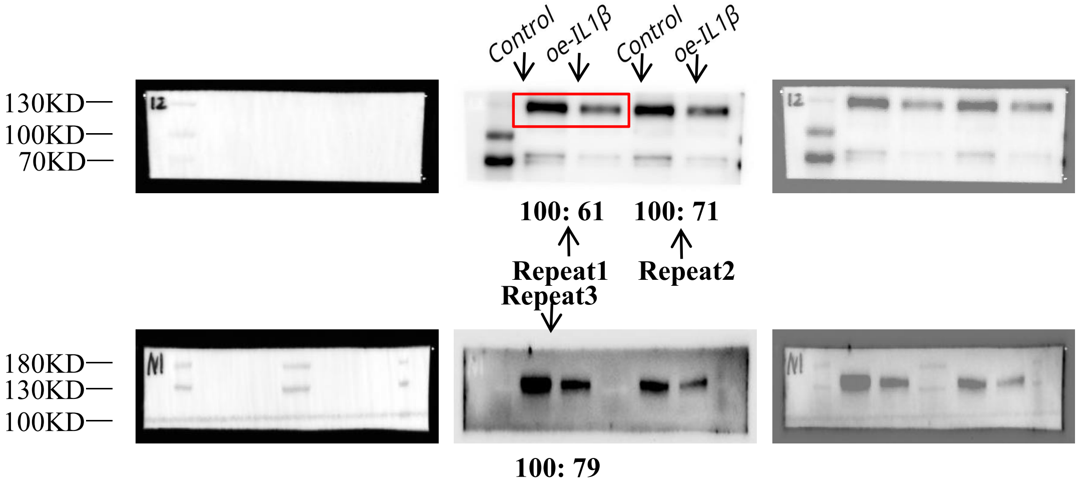

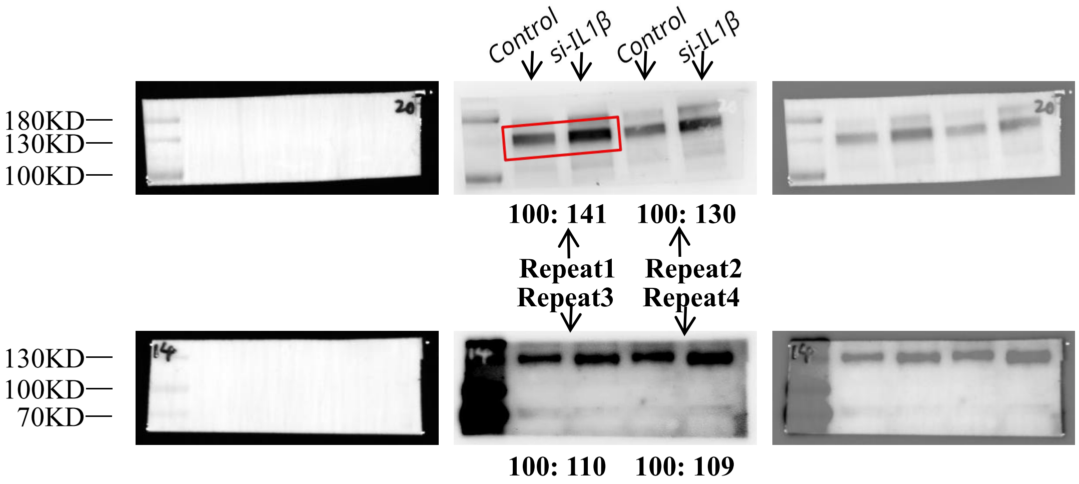

5.2.3 Vimentin (57KD):

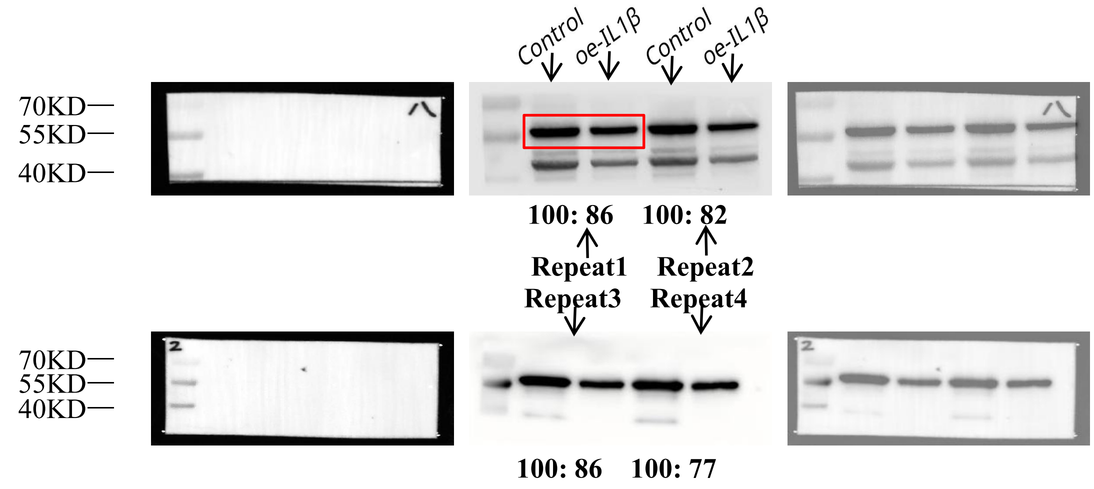

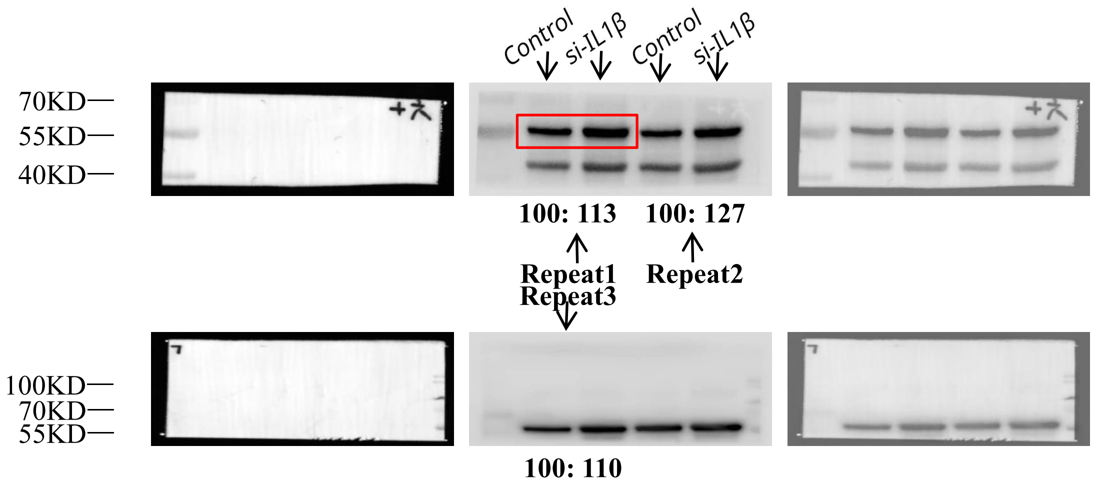

5.2.4 Snail (29KD):

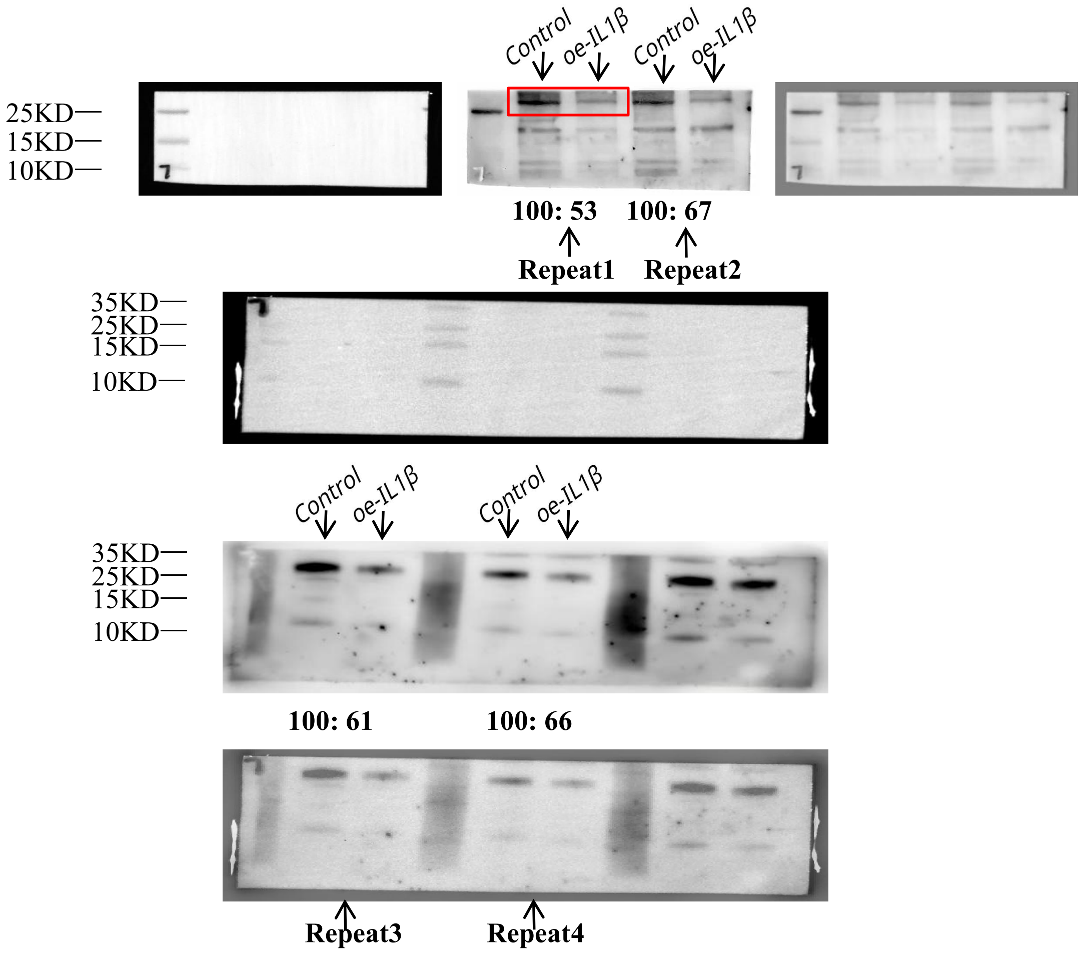

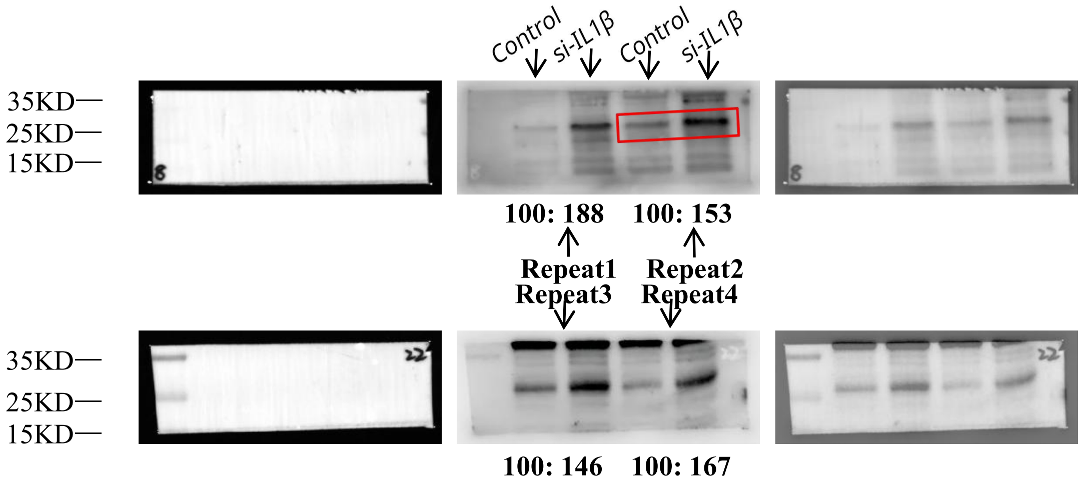

5.2.5 MMP-2 (64KD):

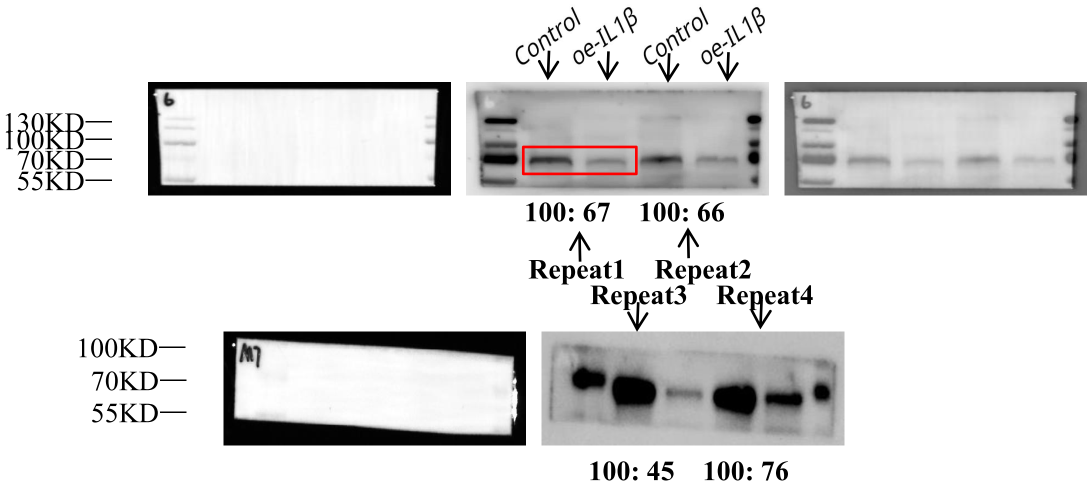

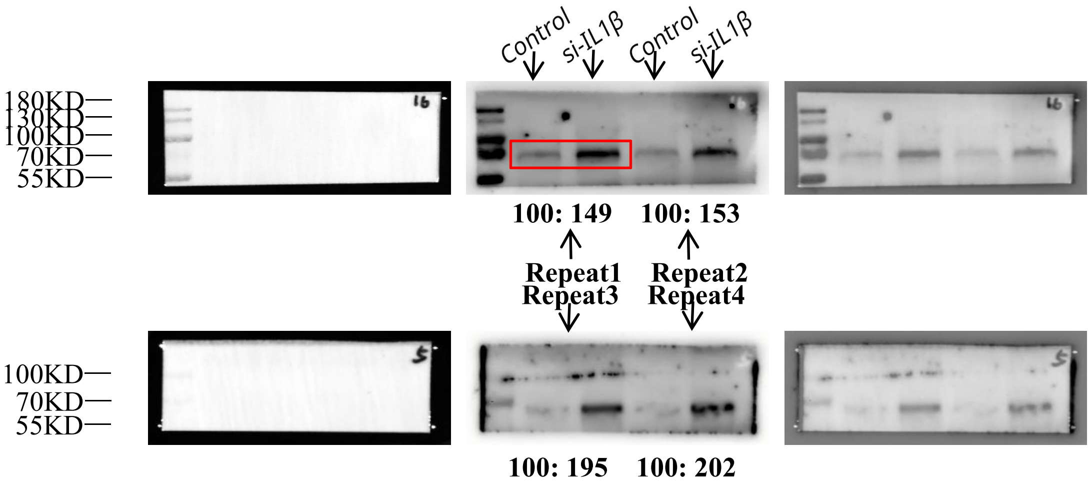

5.2.6 MMP-9 (84KD):

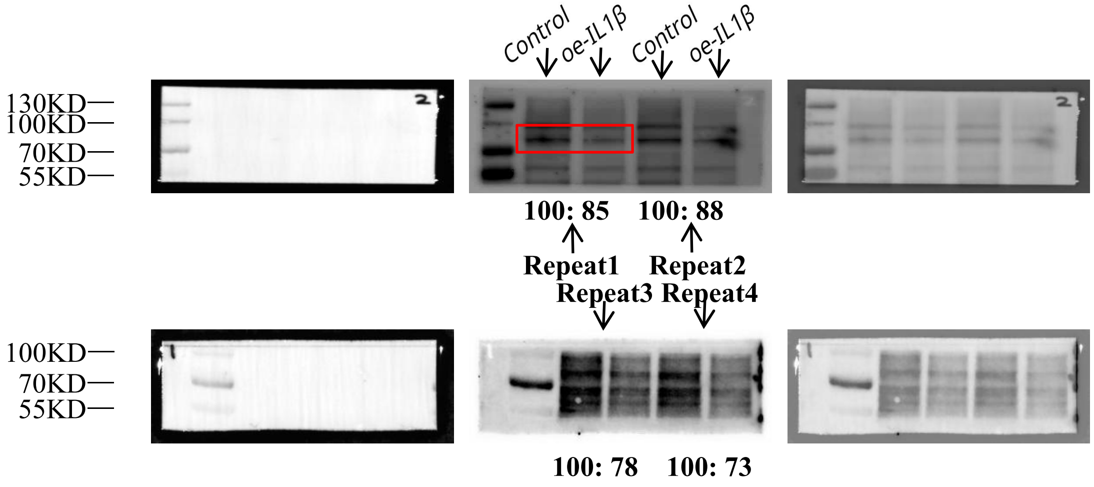

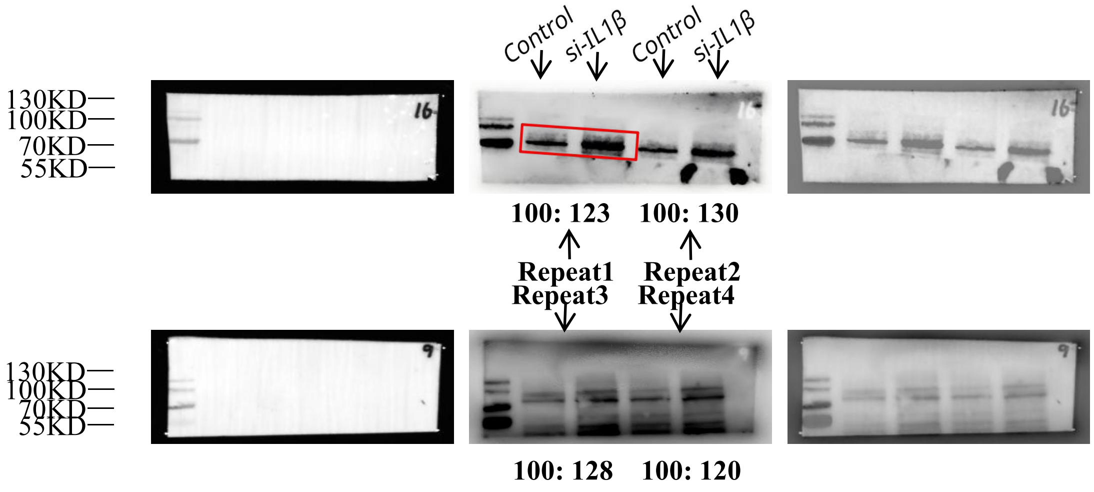

5.2.7 β-actin (42KD):

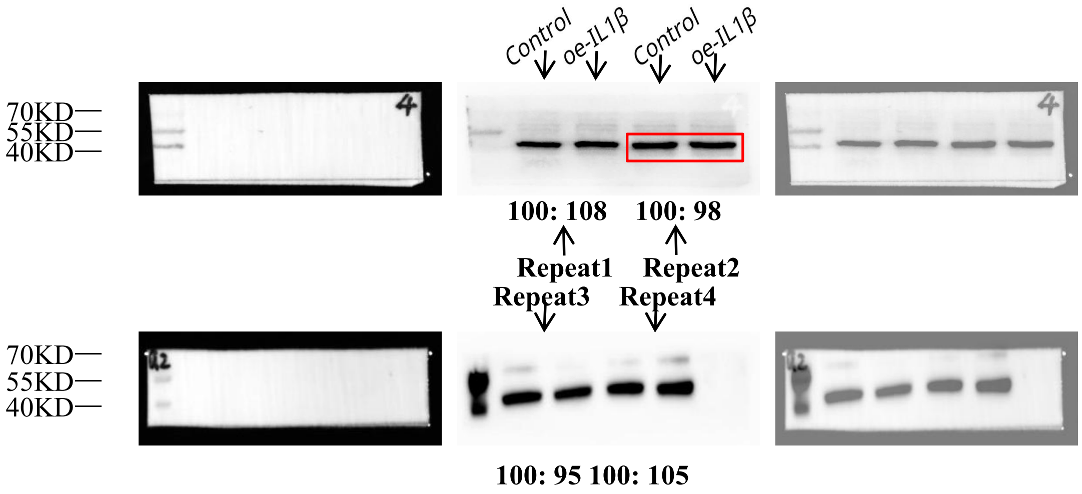

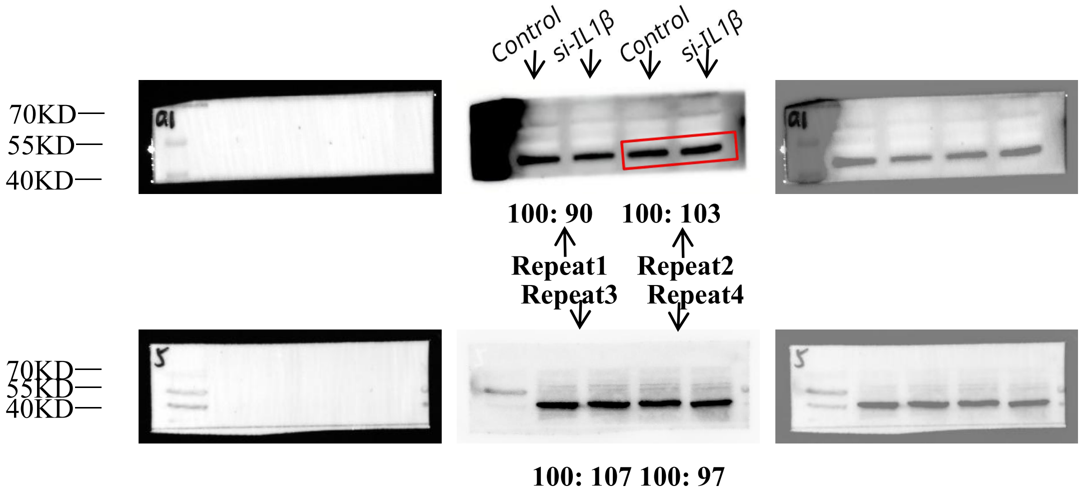

6 Figure 6E and 6H:

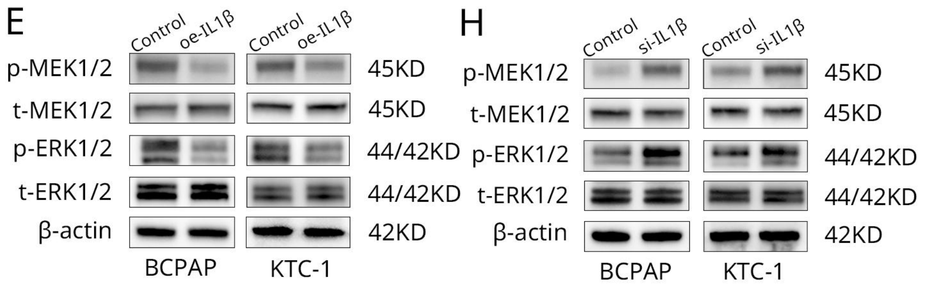

6.1 BCPAP

6.1.1 p-MEK1/2 (45KD):

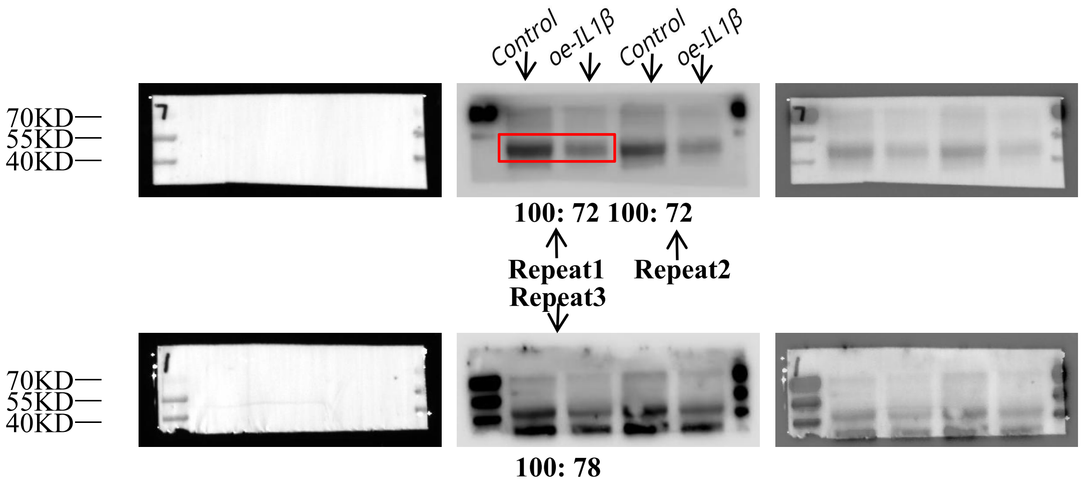

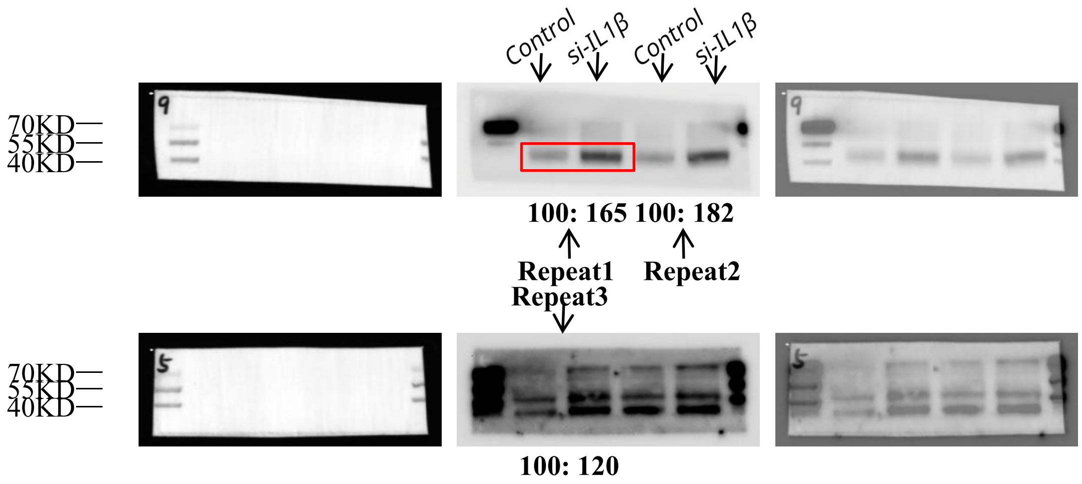

6.1.2 t-MEK1/2 (45KD):

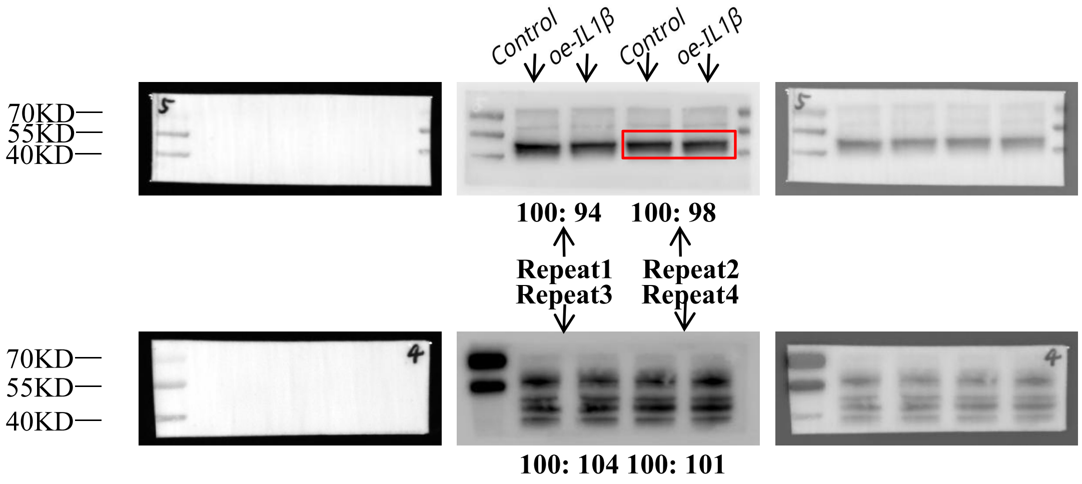

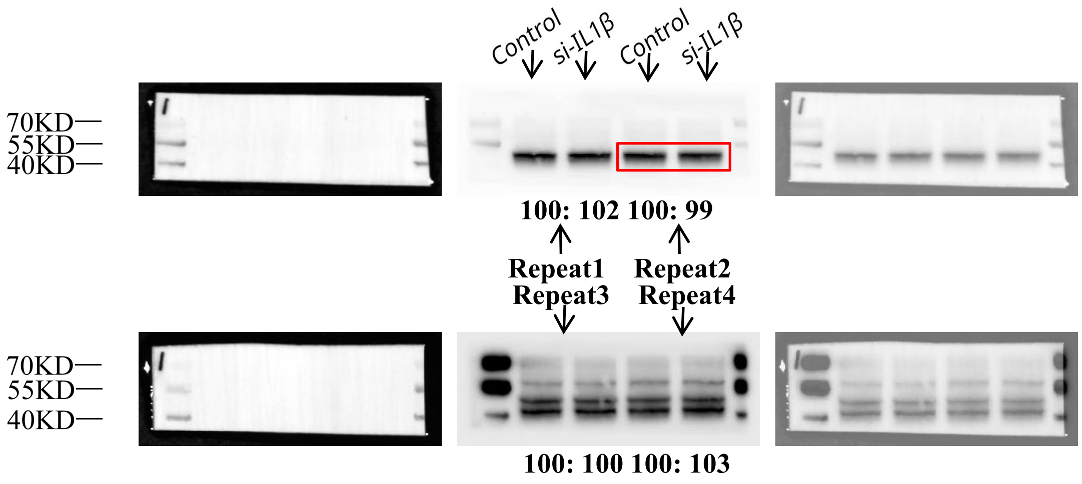

6.1.3 p-ERK1/2 (44/42KD):

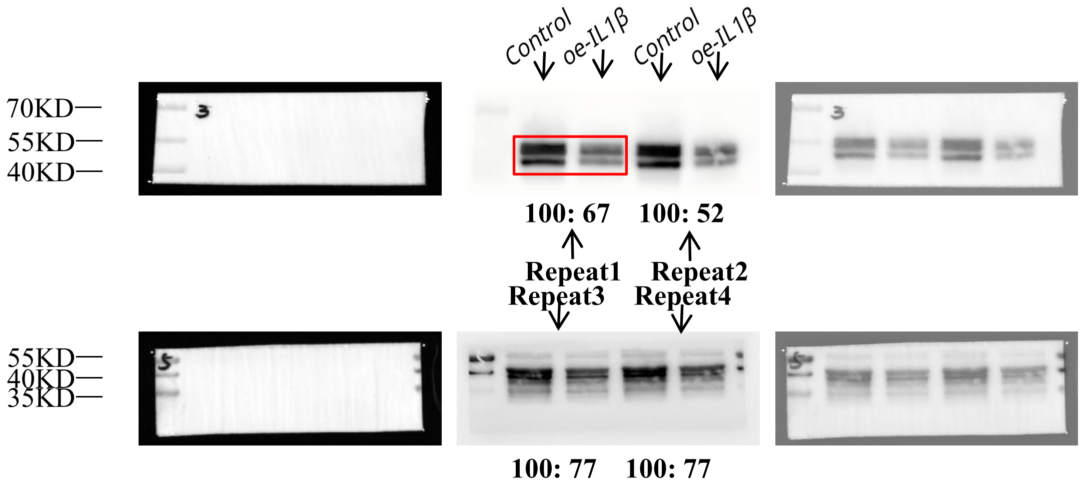

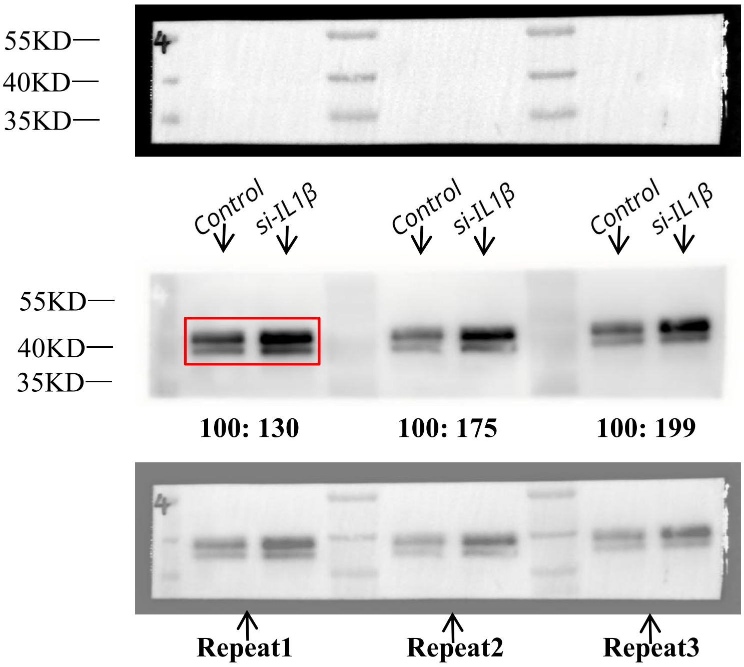

6.1.4 t-ERK1/2 (44/42KD):

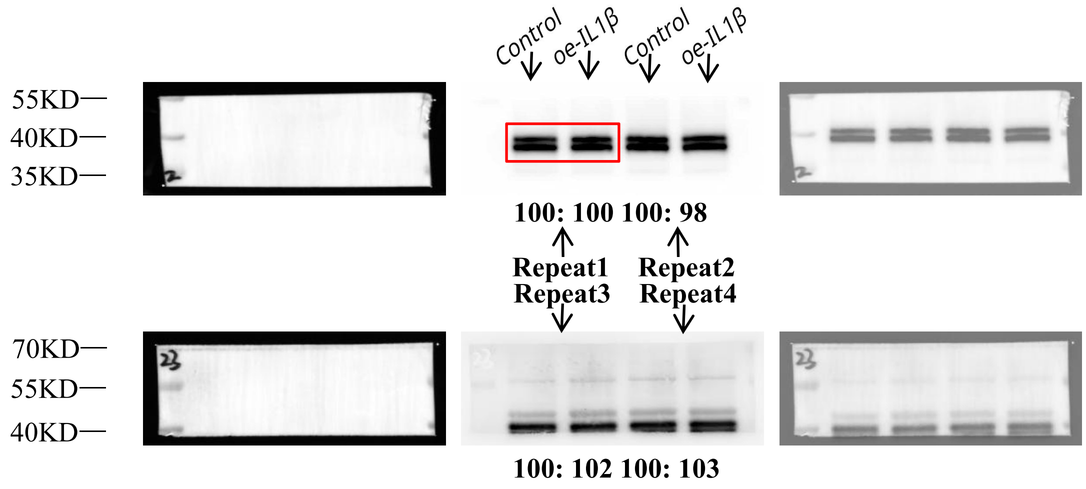

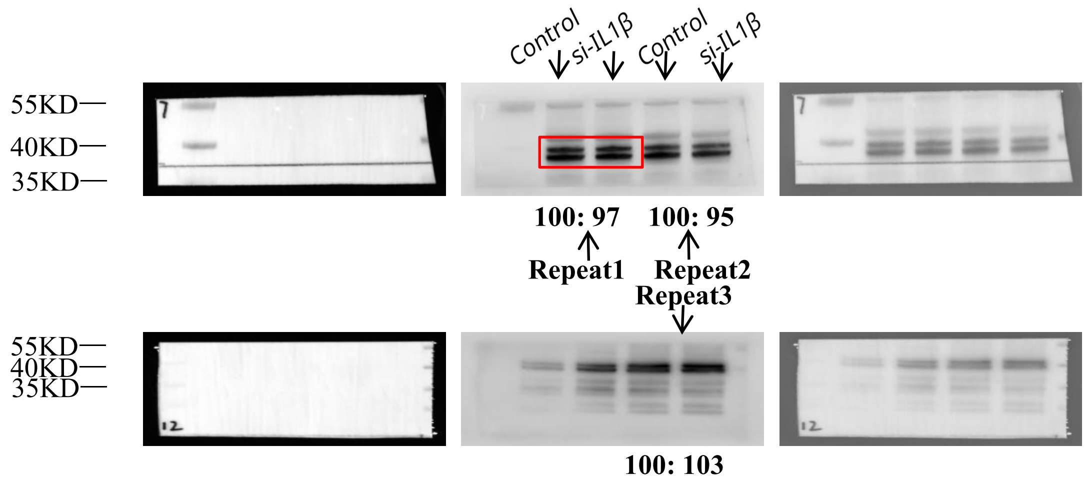

6.1.5 β-actin (42KD):

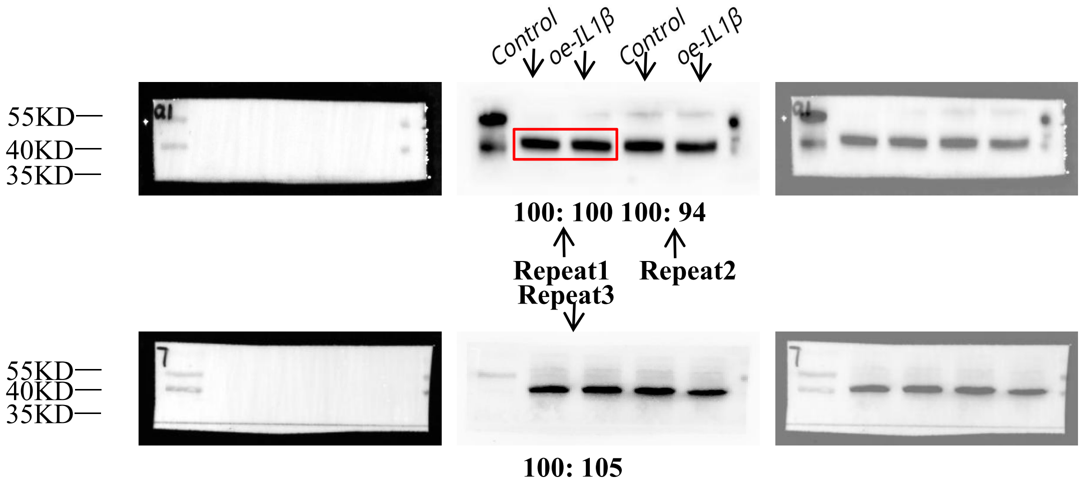

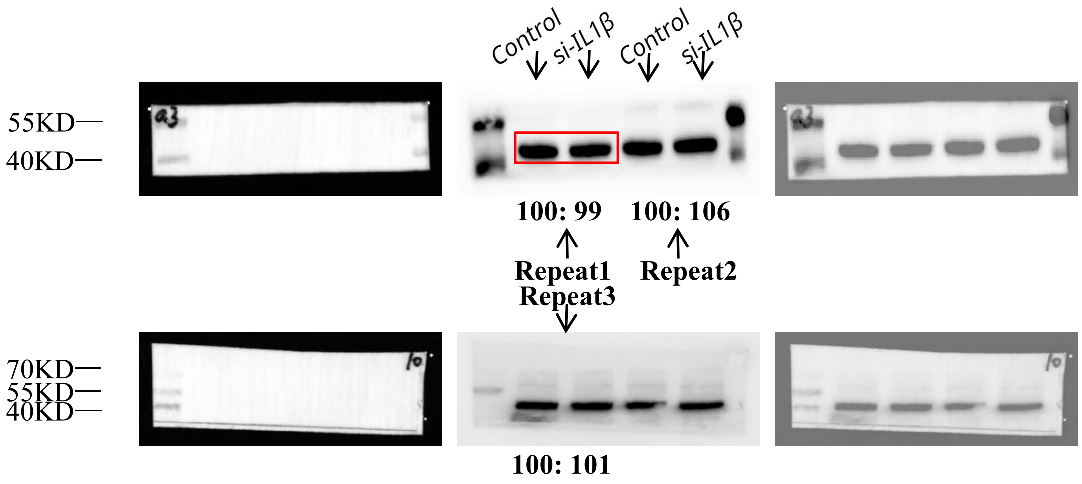

6.2 KTC-1

6.2.1 p-MEK1/2 (45KD):

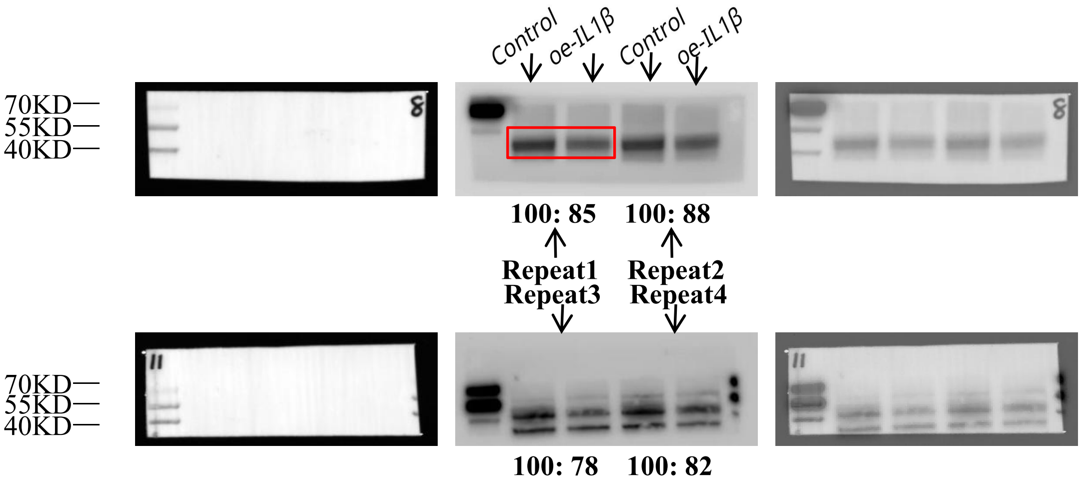

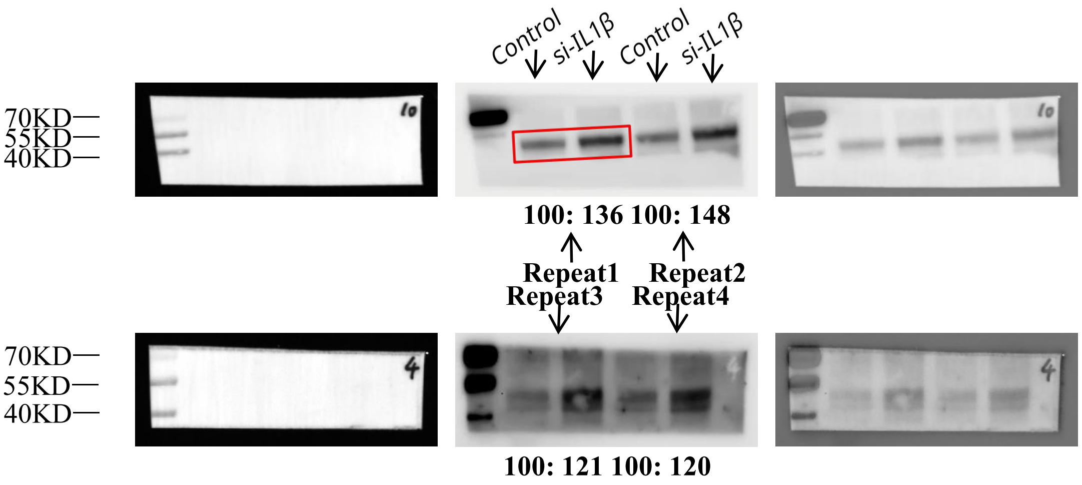

6.2.2 t-MEK1/2 (45KD):

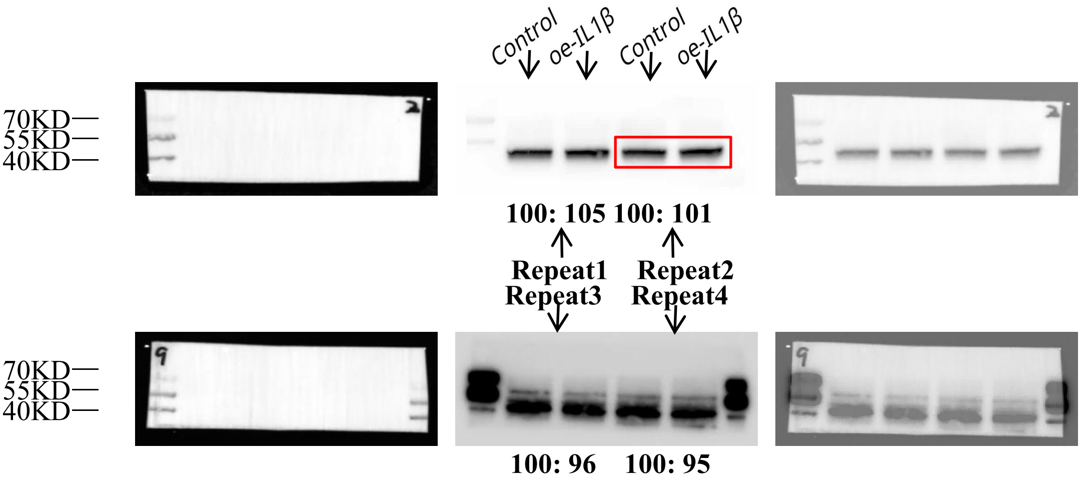

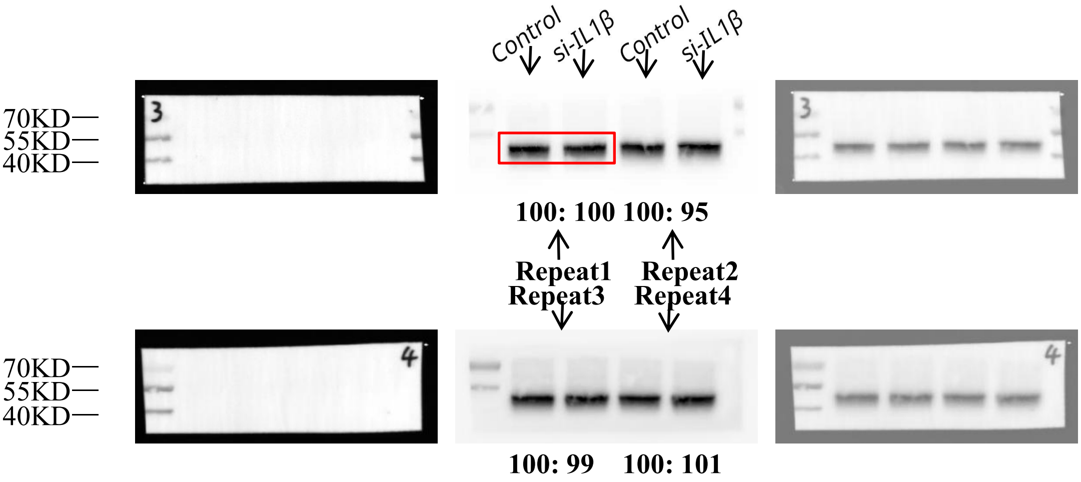

6.2.3 p-ERK1/2 (44/42KD):

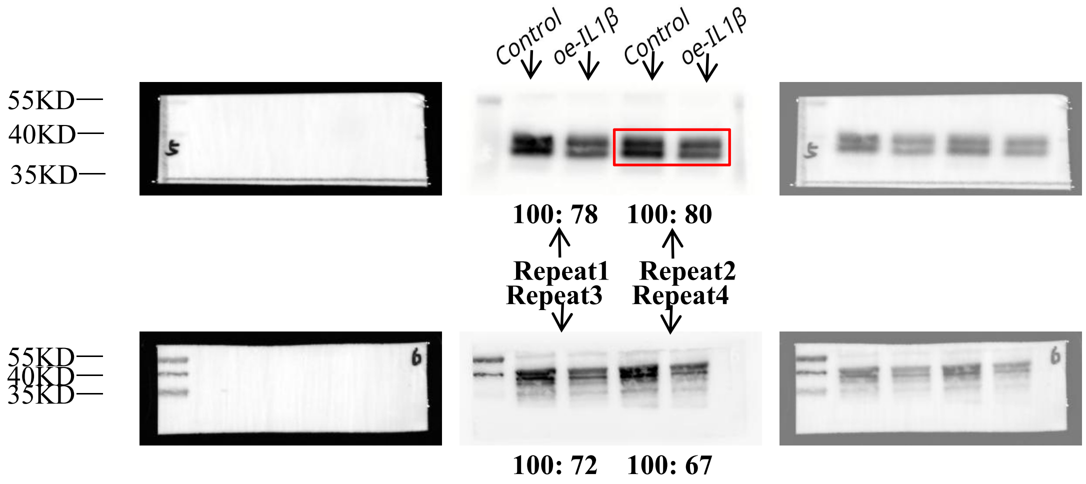

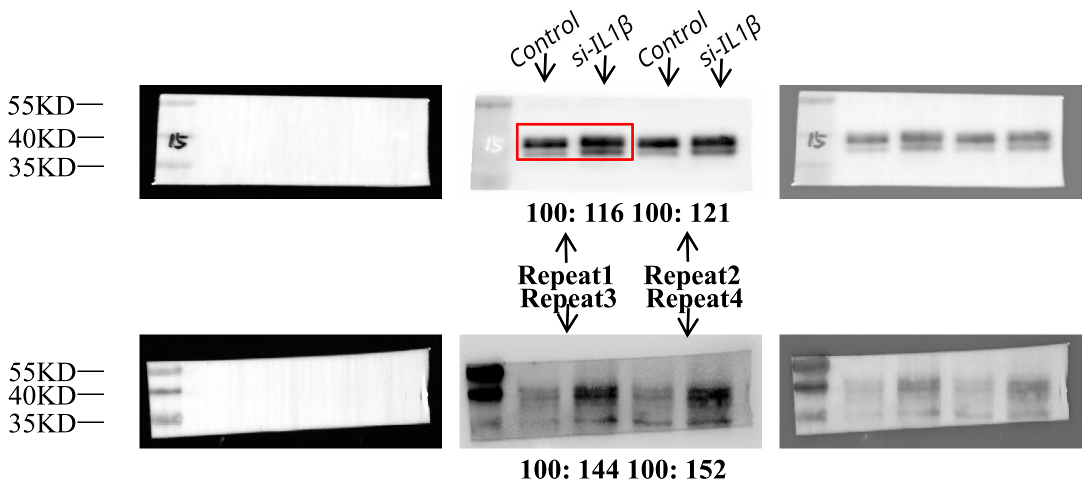

6.2.4 t-ERK1/2 (44/42KD):

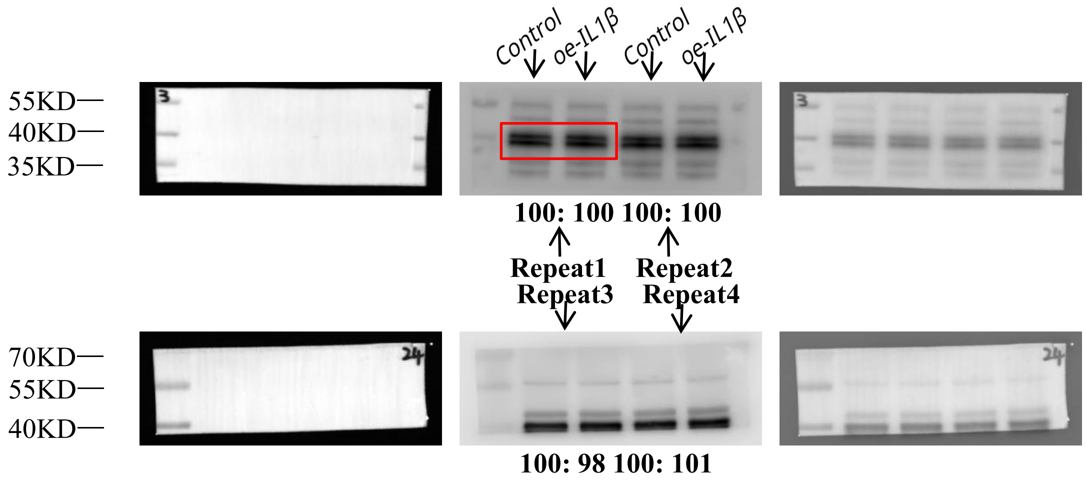

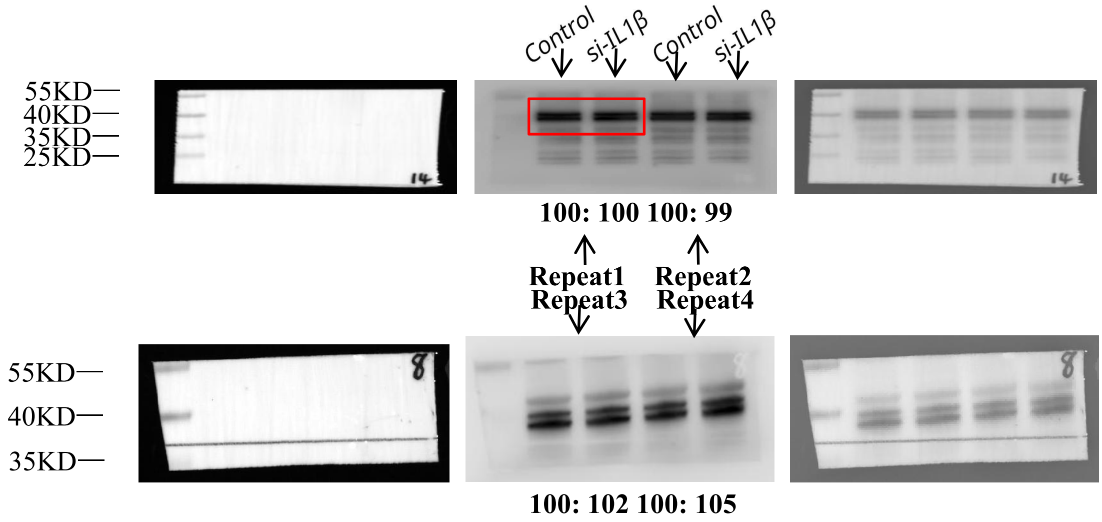

6.2.5 β-actin (42KD):

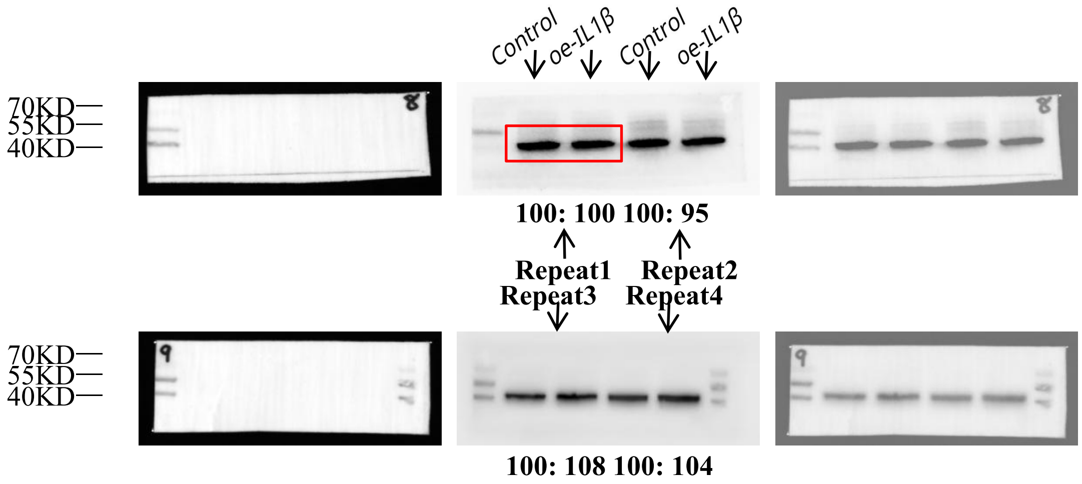

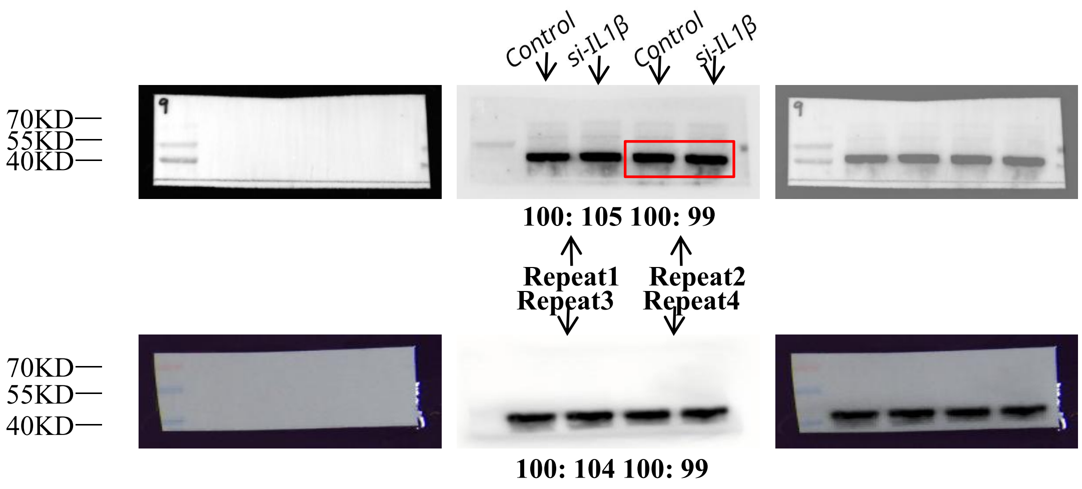

7 Figure 7B and 7C:

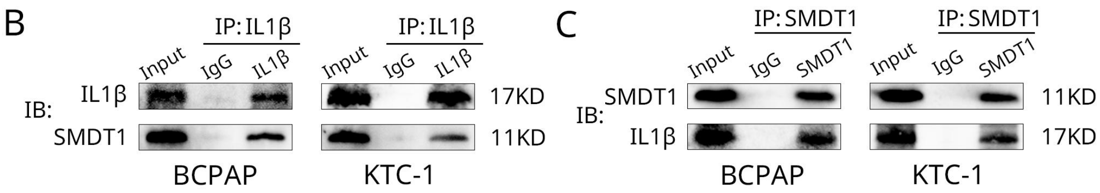

7.1 BCPAP

7.1.1 IL1β (17KD):

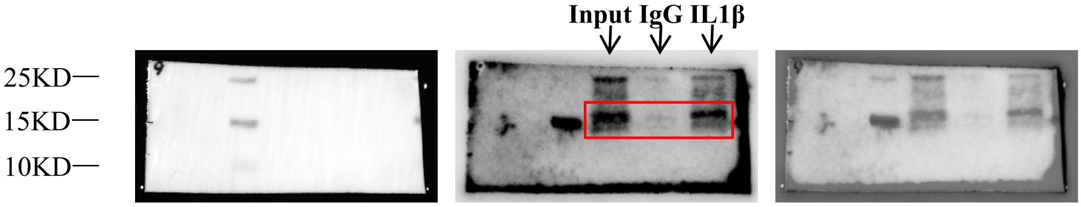

7.1.2 SMDT1 (11KD):

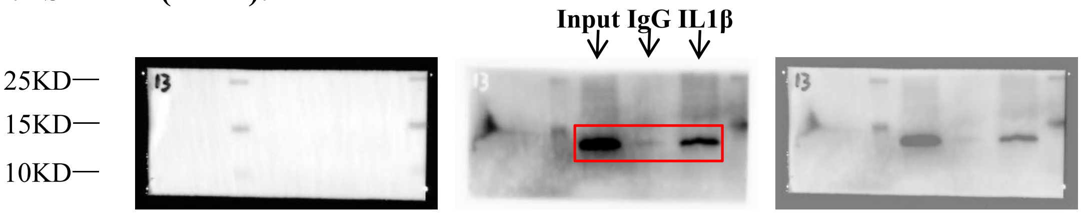

7.1.3 IL1β (17KD):

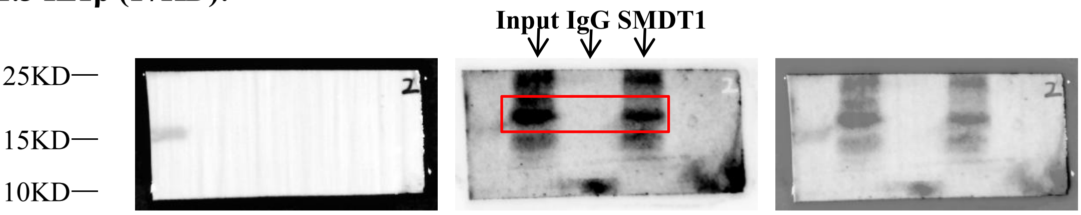

7.1.4 SMDT1 (11KD):

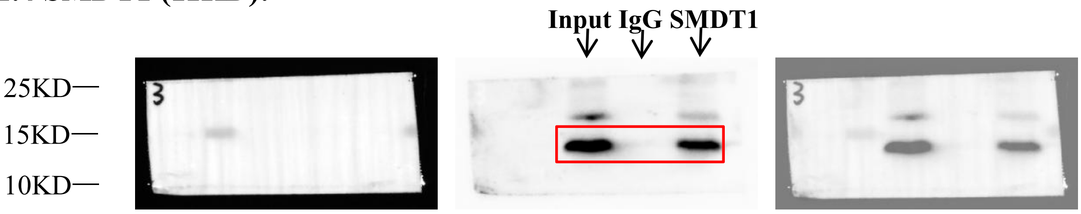

7.2 KTC-1

7.2.1 IL1β (17KD):

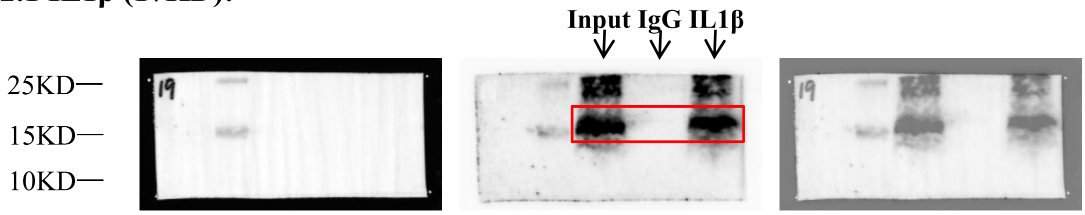

7.2.2 SMDT1 (11KD):

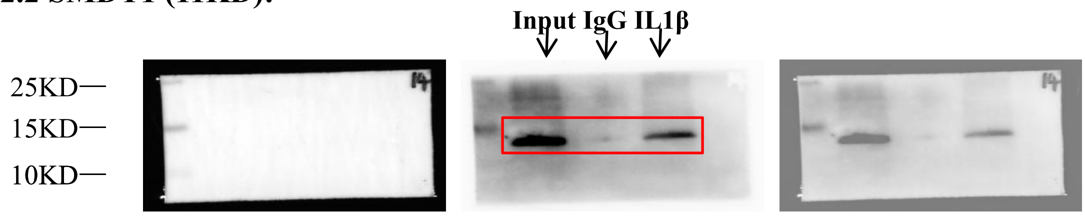

7.2.3 IL1β (17KD):

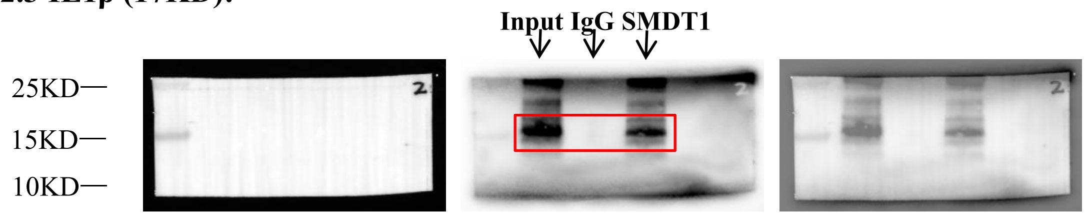

7.1.4 SMDT1 (11KD):

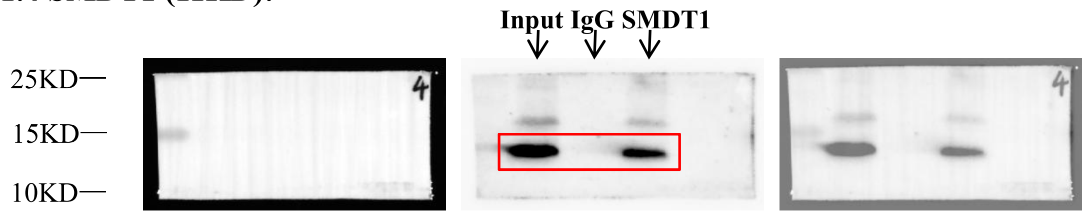

8 Figure 7E:

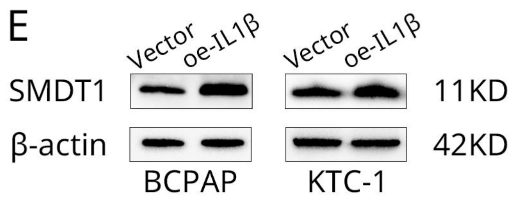

8.1 BCPAP

8.1.1 SMDT1 (11KD):

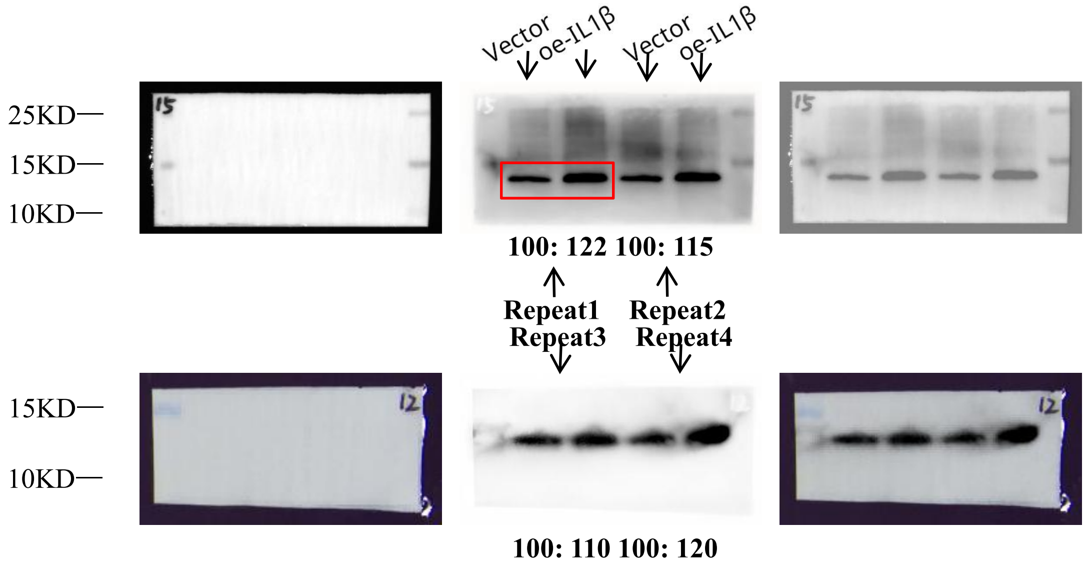

8.1.2 β-actin (42KD):

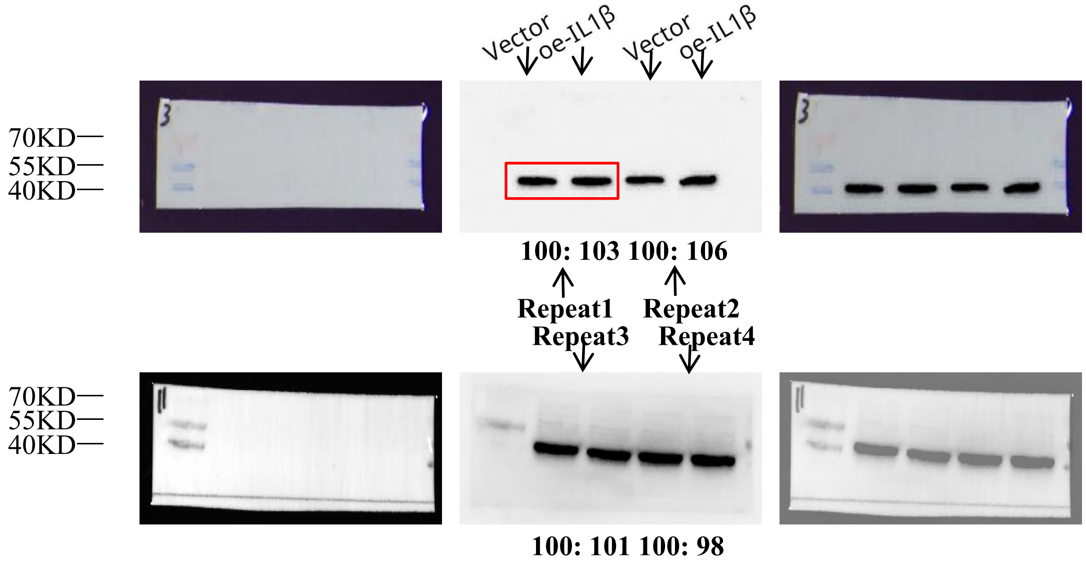

8.2 KTC-1

8.2.1 SMDT1 (11KD):

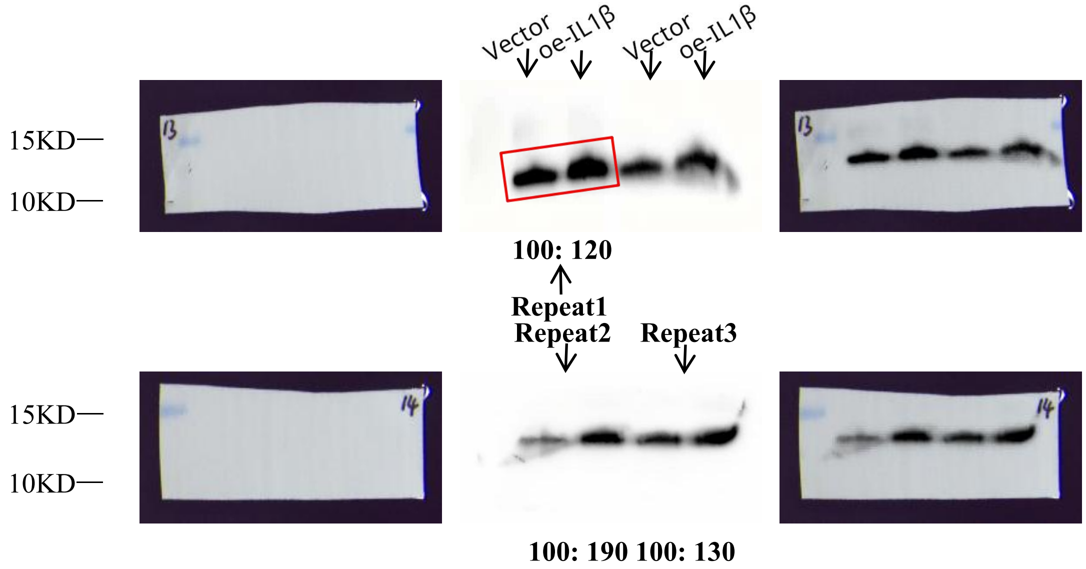

8.2.2 β-actin (42KD):

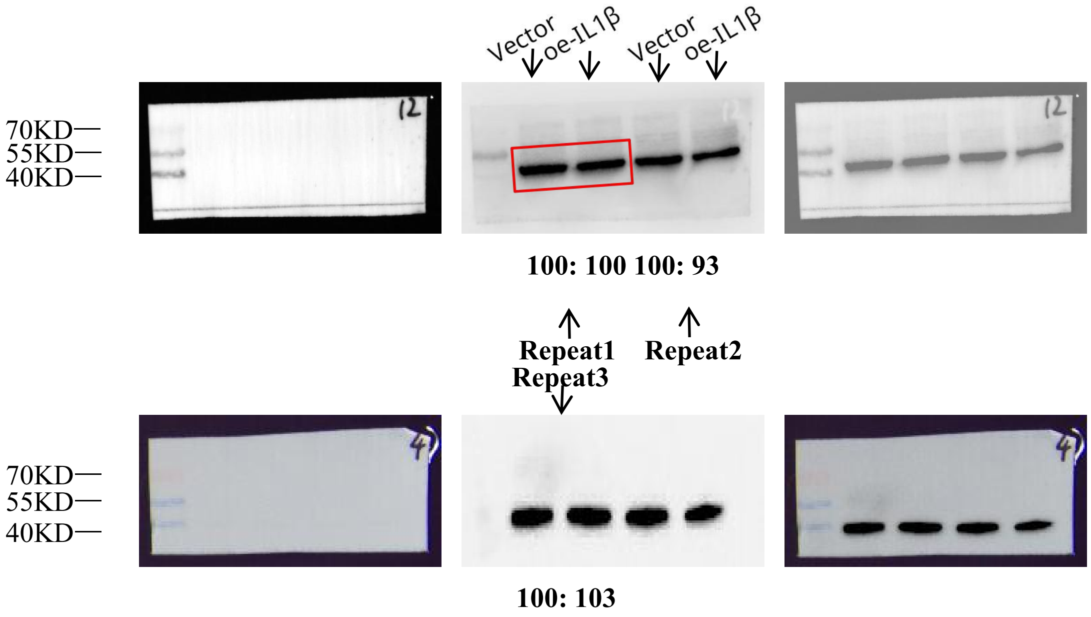

9 Figure 8E:

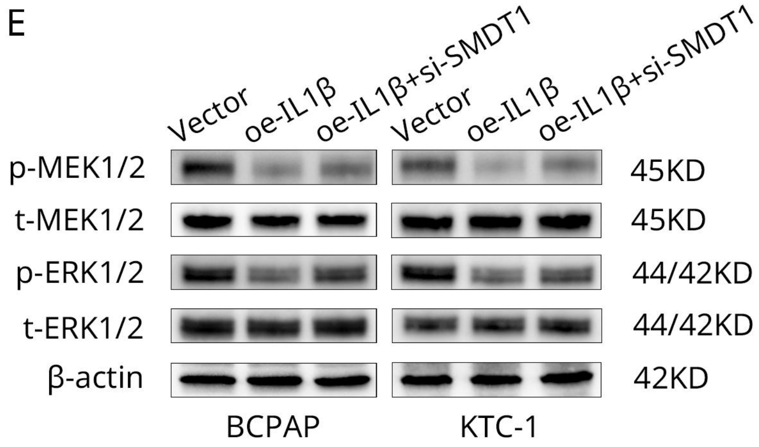

9.1 BCPAP

9.1.1 p-MEK1/2 (45KD):

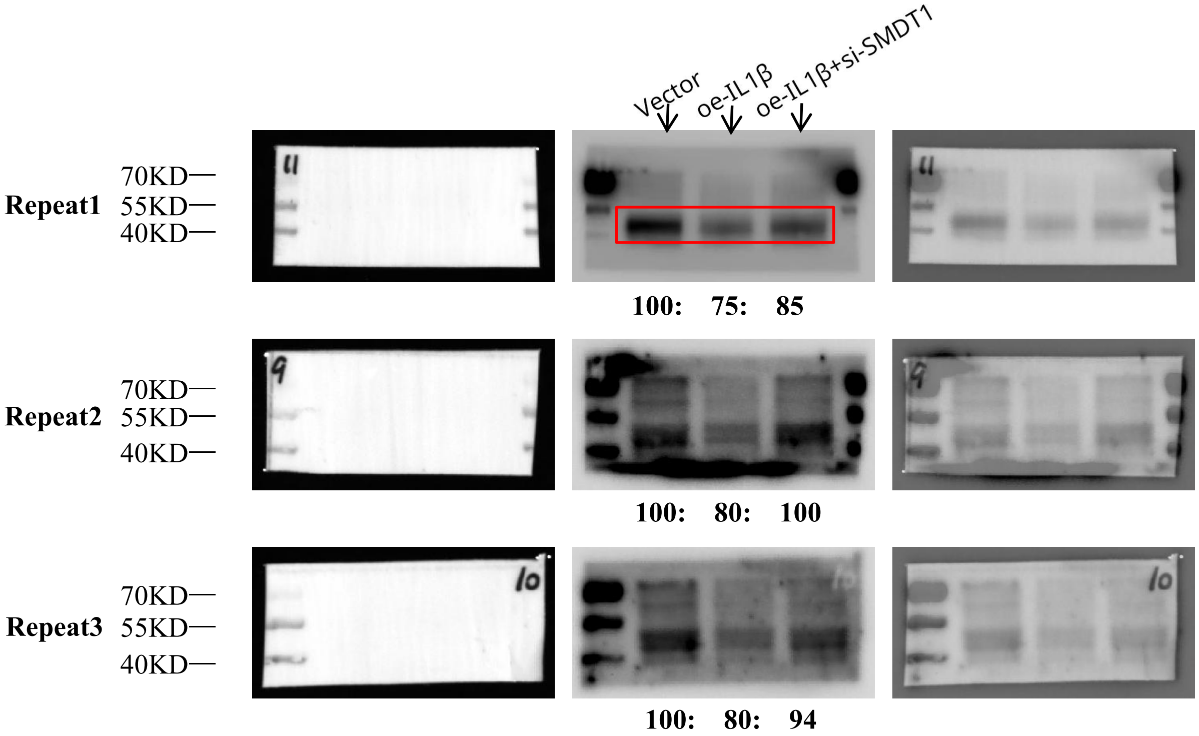

9.1.2 t-MEK1/2 (45KD):

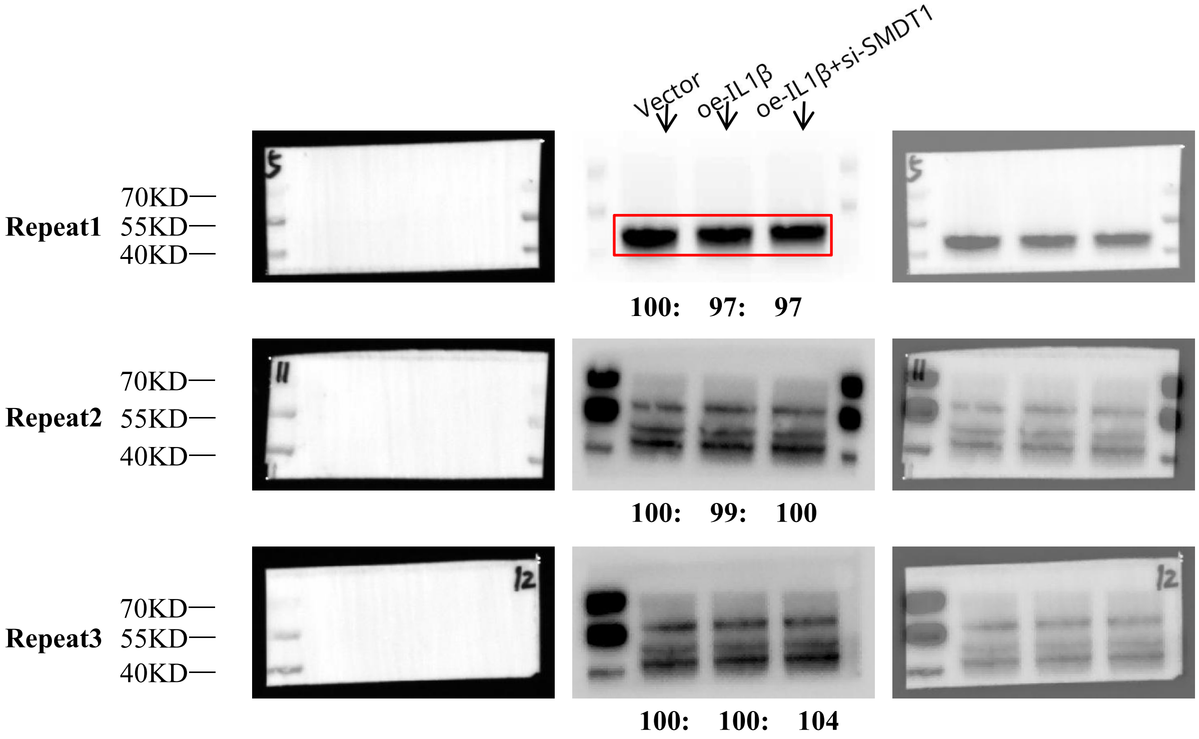

9.1.3 p-ERK1/2 (44/42KD):

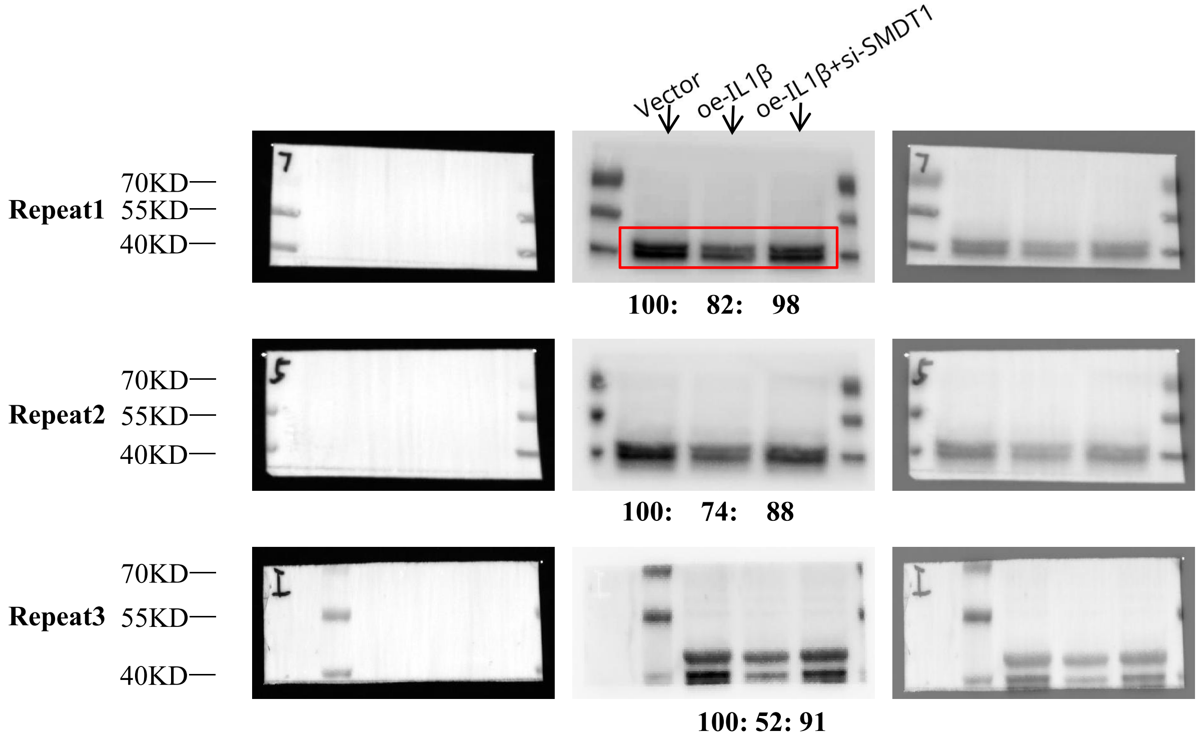

9.1.4 t-ERK1/2 (44/42KD):

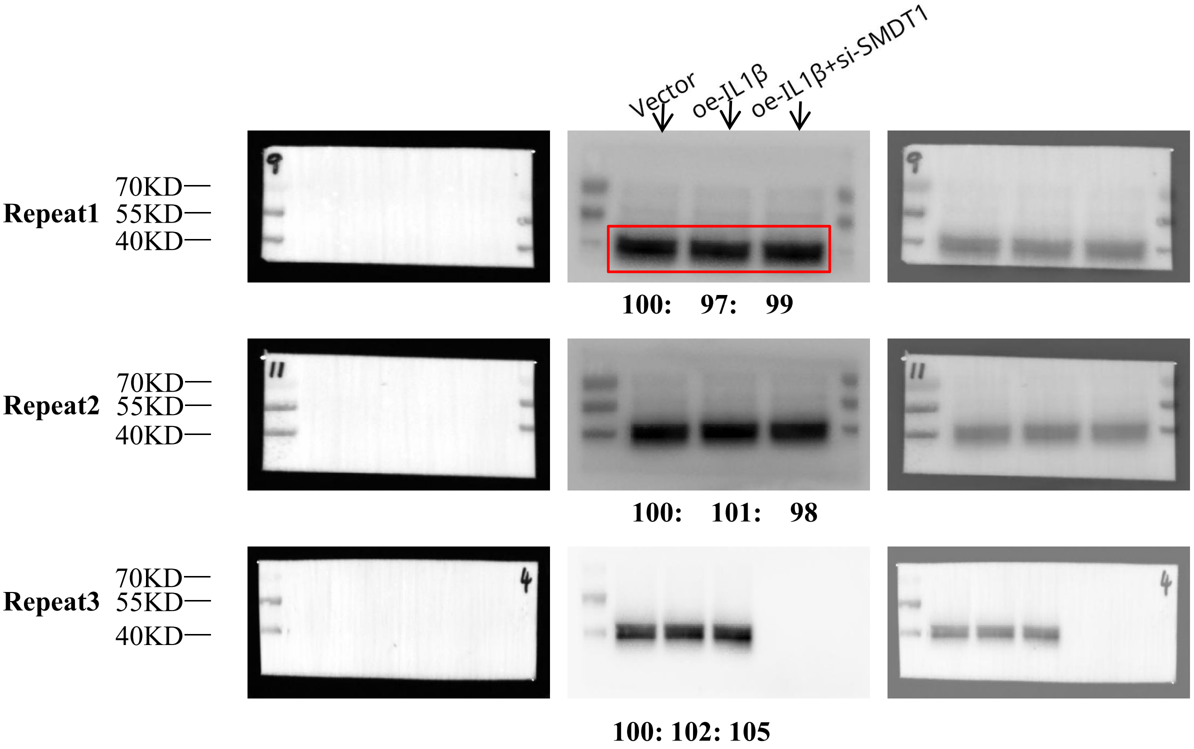

9.1.5 β-actin (42KD):

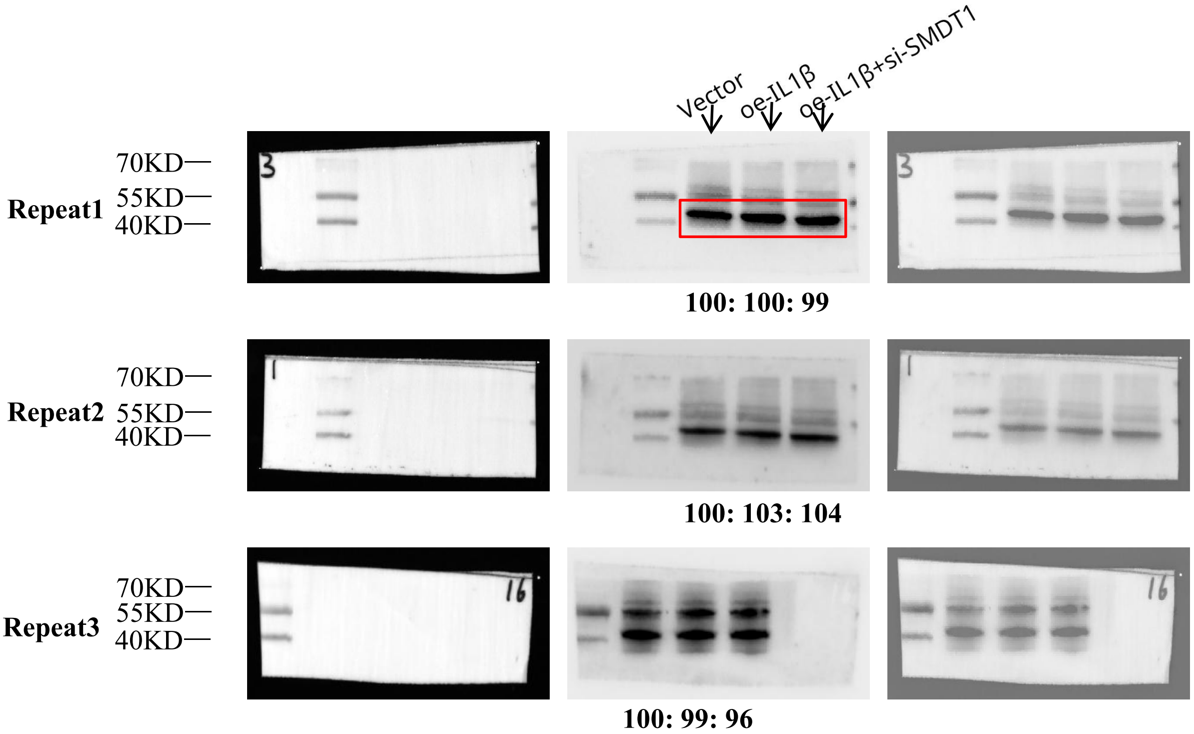

9.2 KTC-1

9.2.1 p-MEK1/2 (45KD):

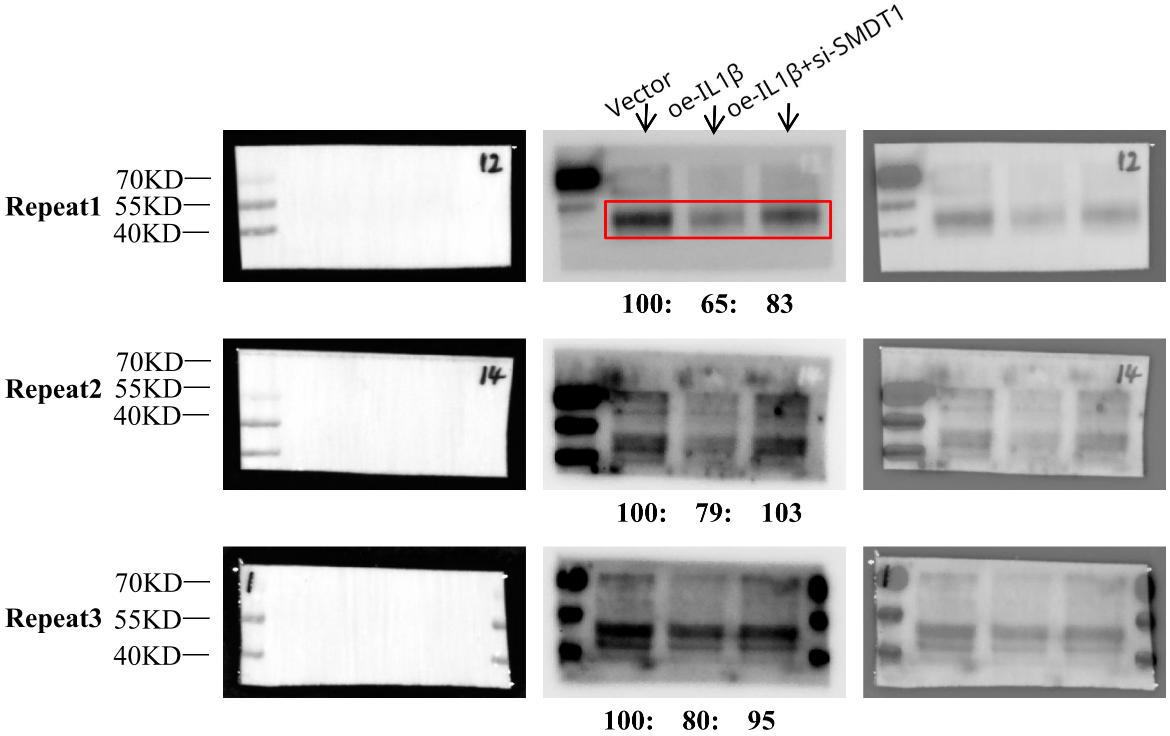

9.2.2 t-MEK1/2 (45KD):

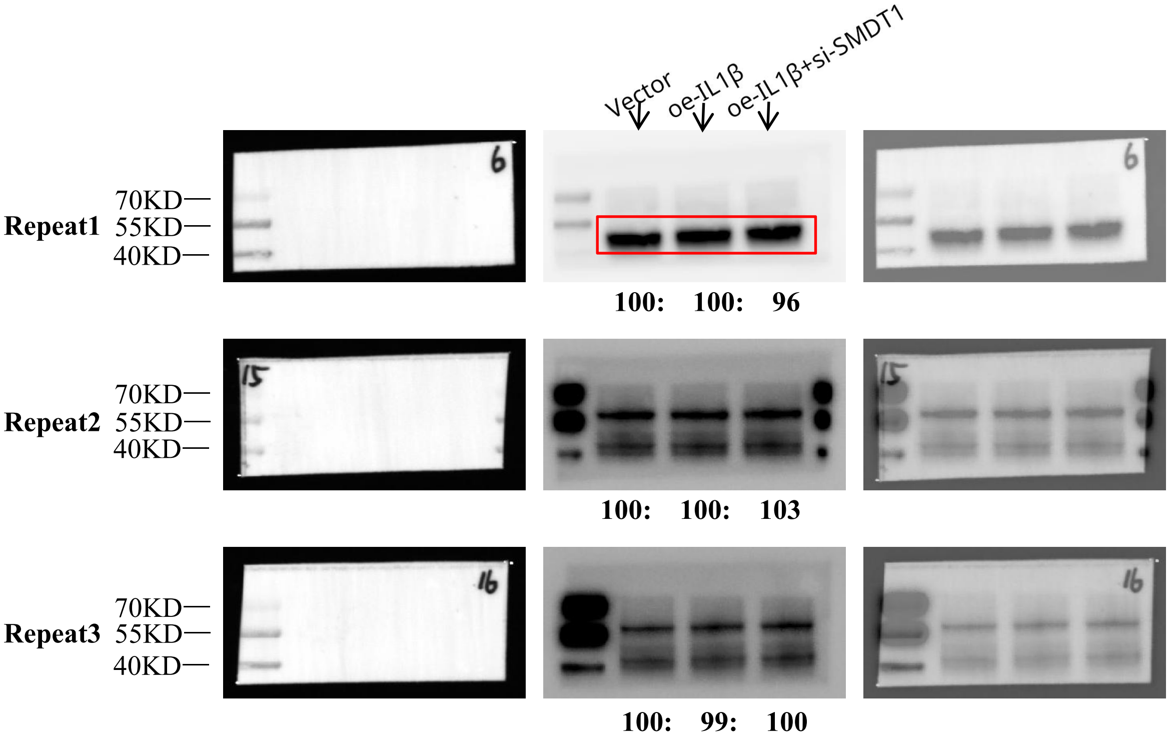

9.2.3 p-ERK1/2 (44/42KD):

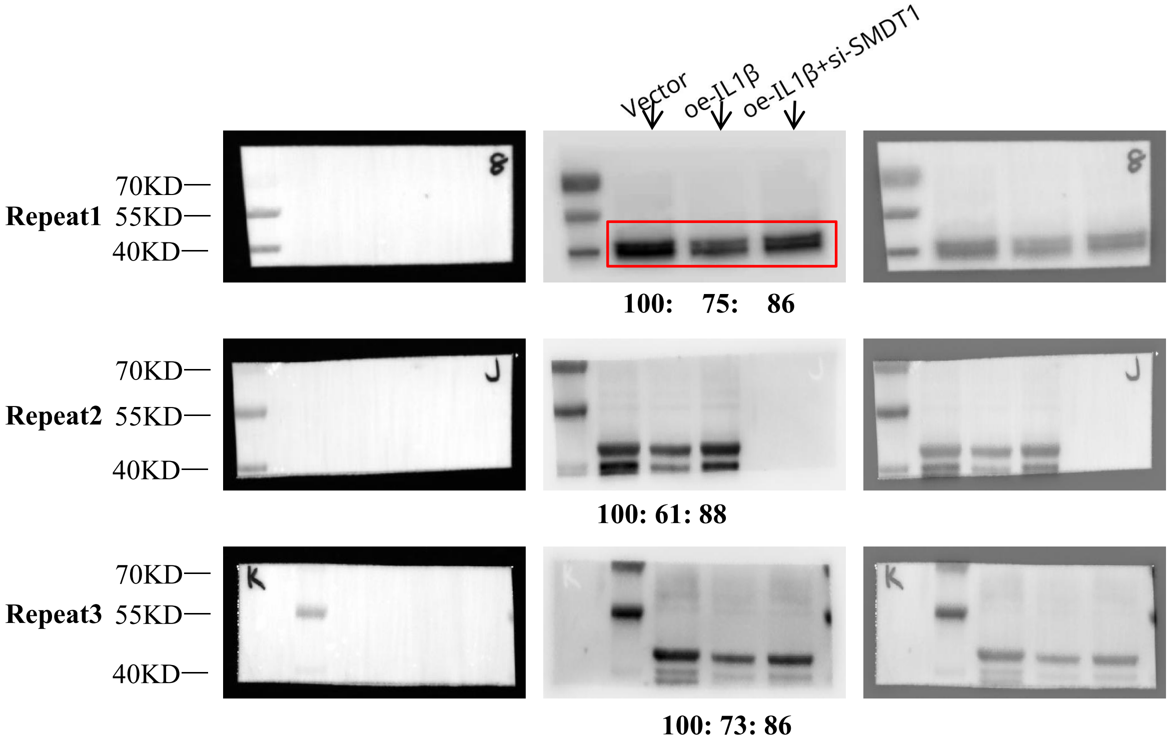

9.2.4 t-ERK1/2 (44/42KD):

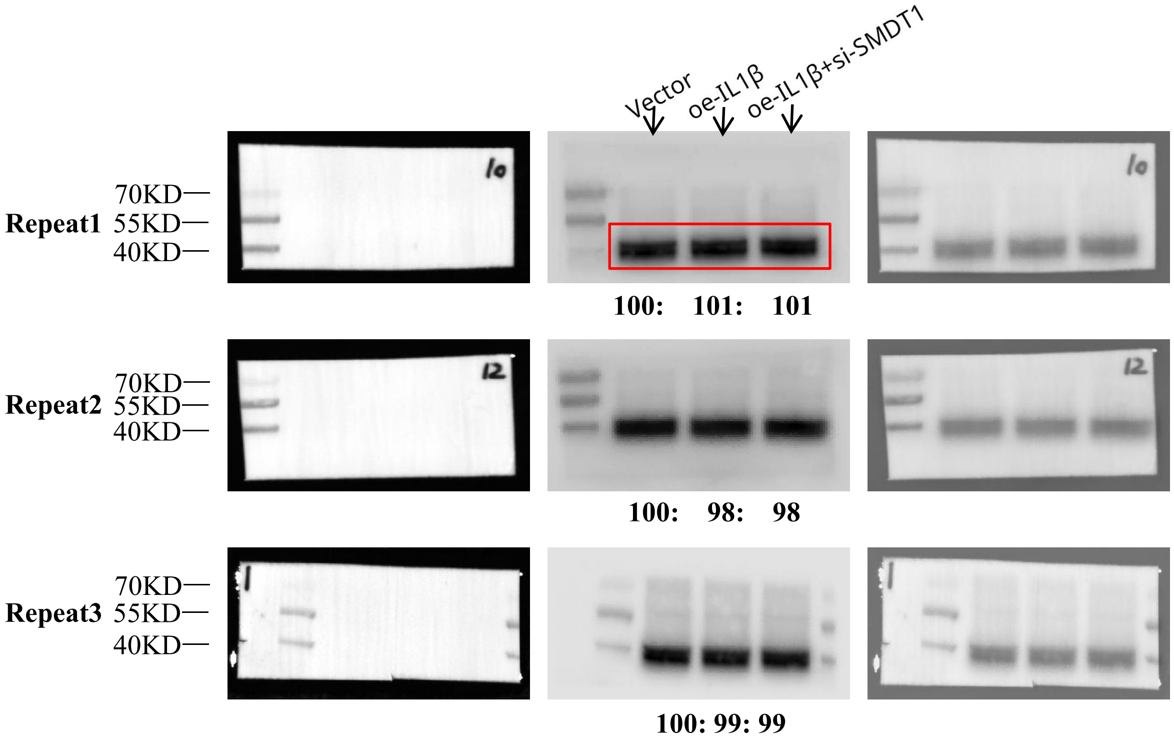

9.2.5 β-actin (42KD):

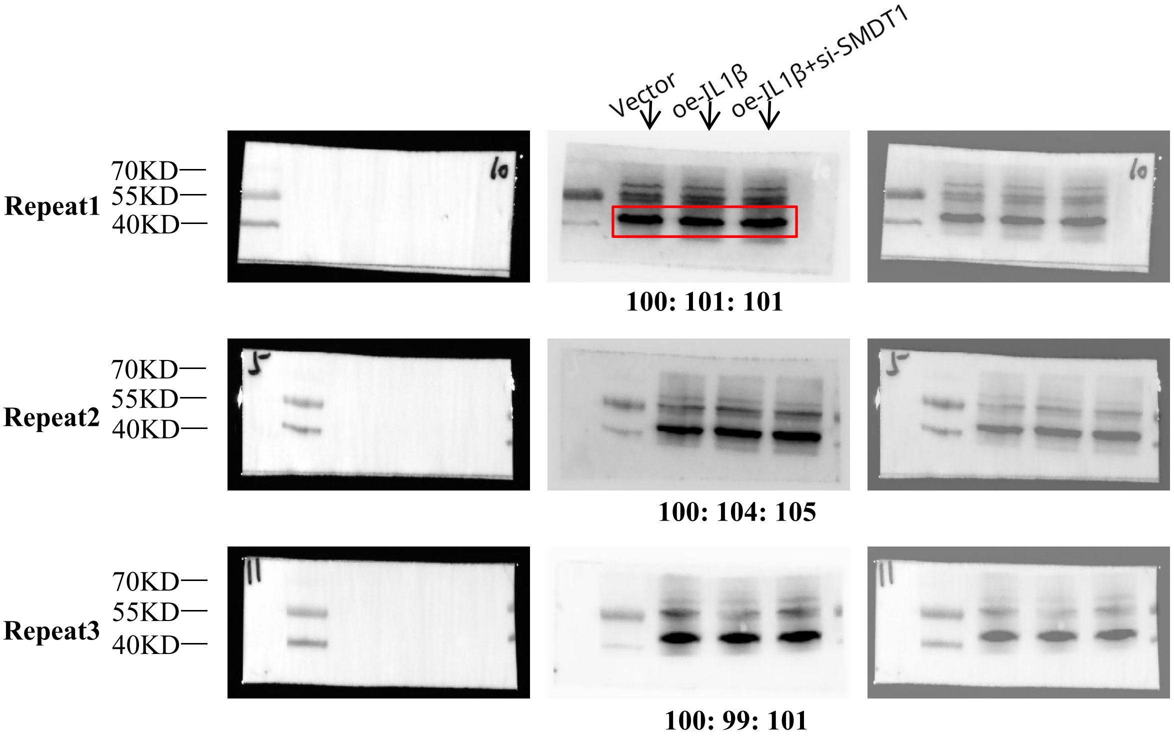

10 Figure 9F:

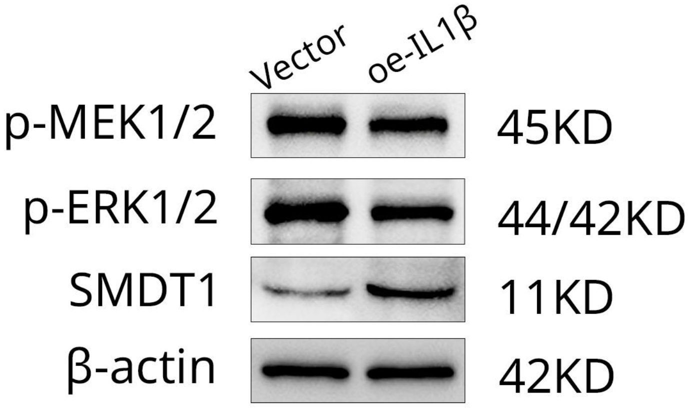

10.1 p-MEK1/2 (45KD):

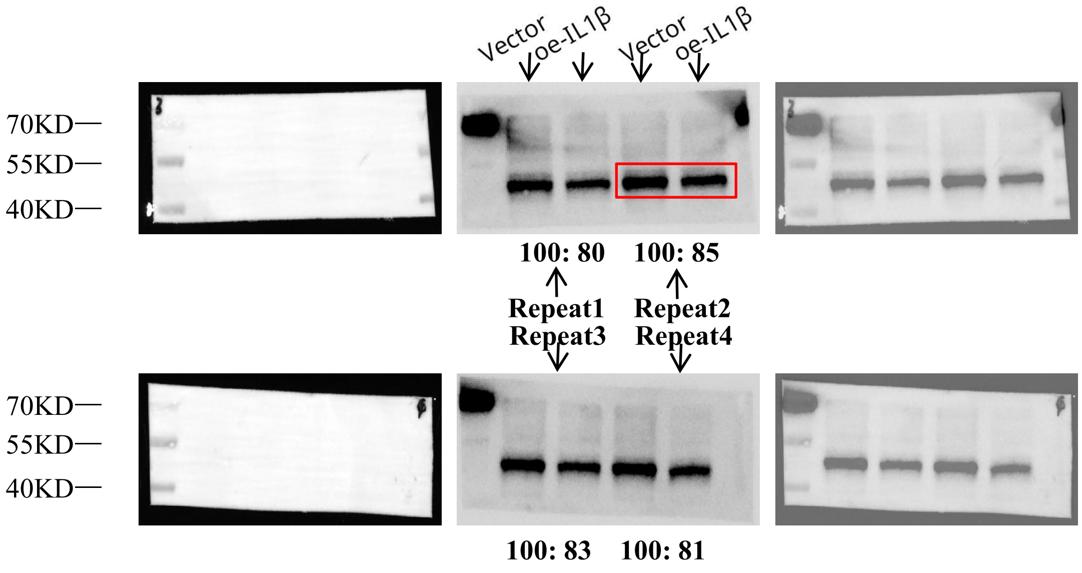

10.2 p-ERK1/2 (44/42KD):

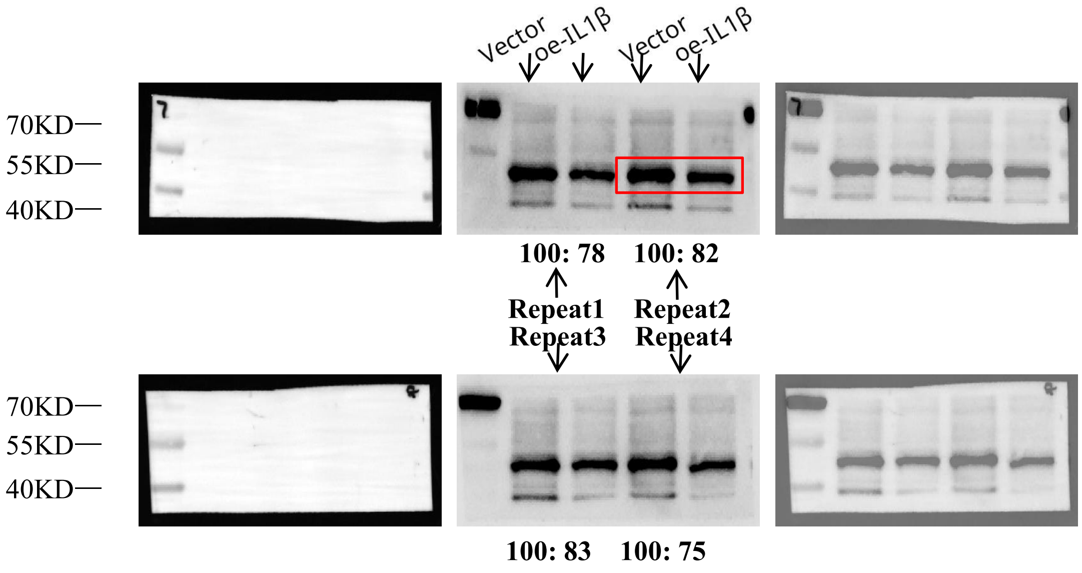

10.3 SMDT1 (11KD):

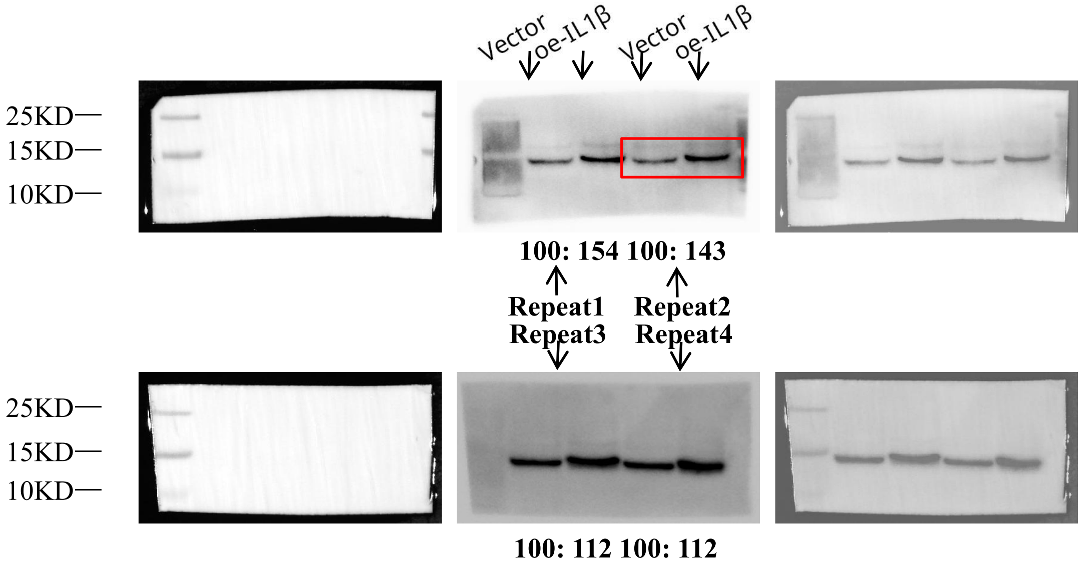

10.4 β-actin (42KD):

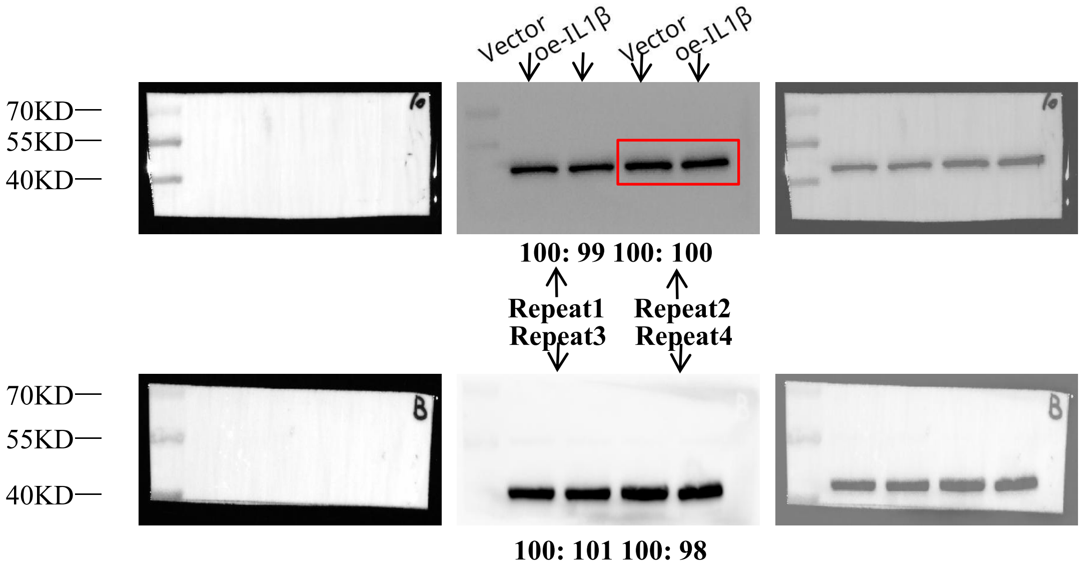

11 Supplementary Figure 2A:

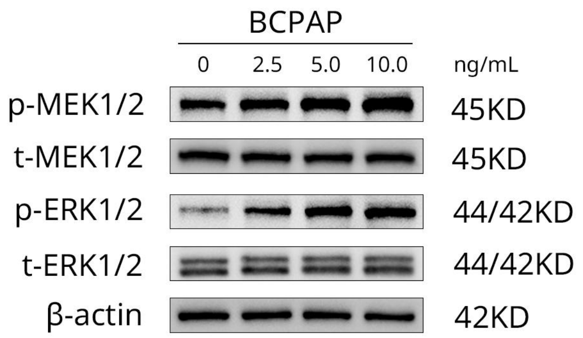

11.1 BCPAP

11.1.1 p-MEK1/2 (45KD):

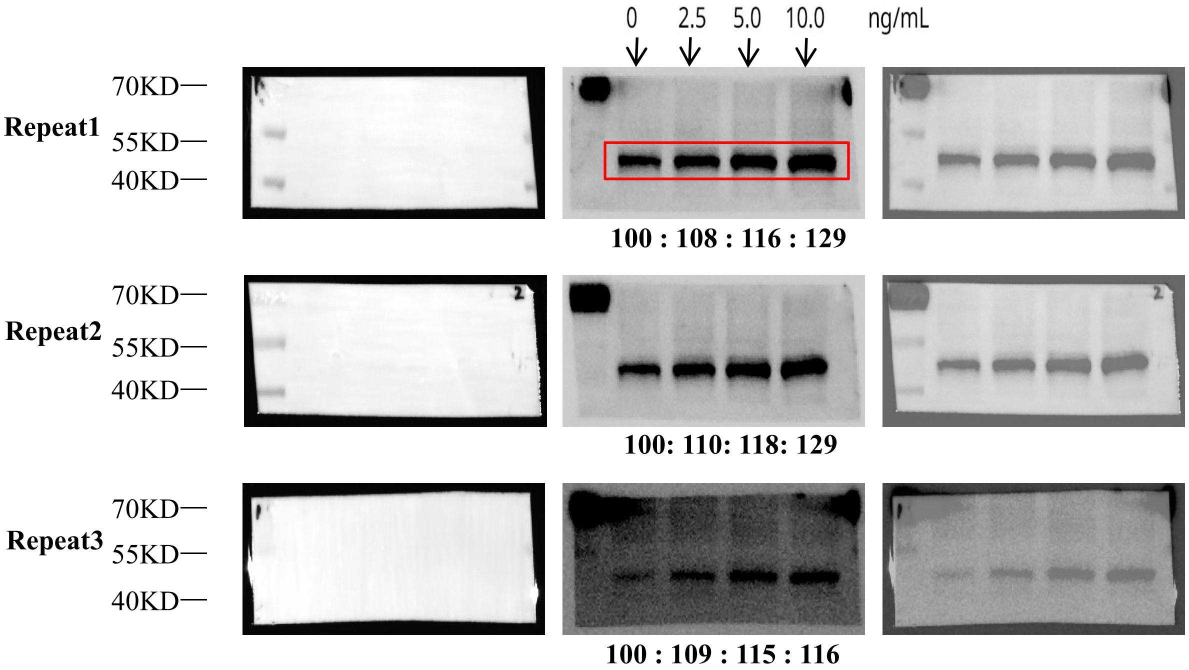

11.1.2 t-MEK1/2 (45KD):

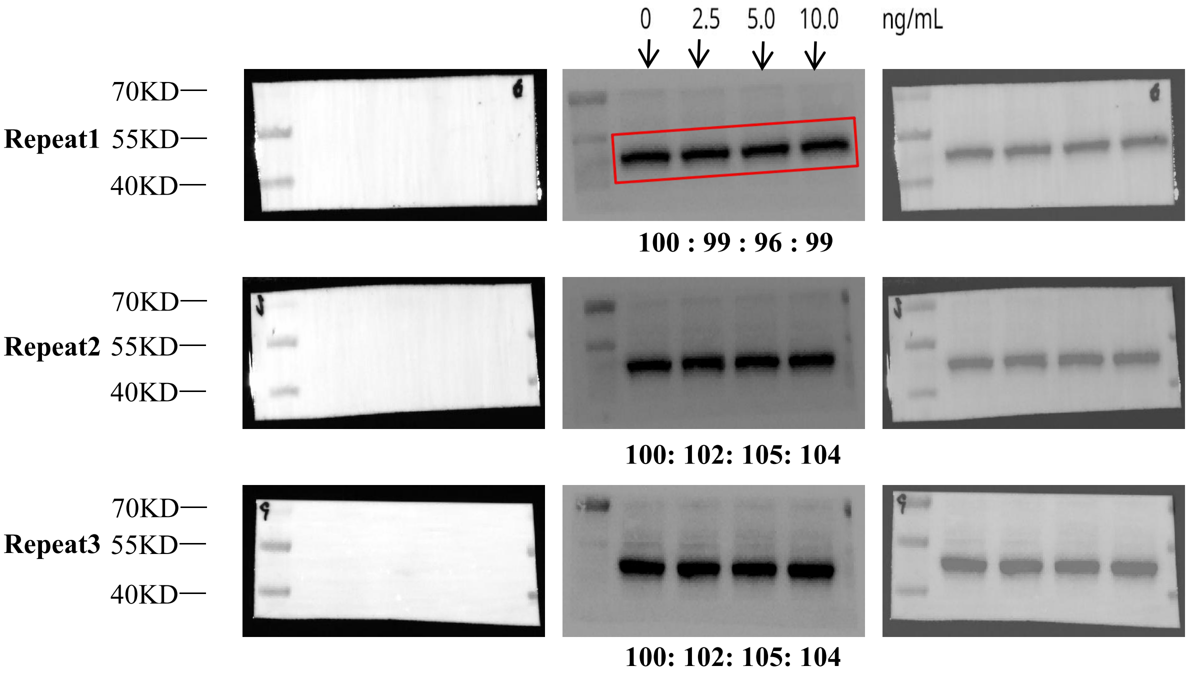

11.1.3 p-ERK1/2 (44/42KD):

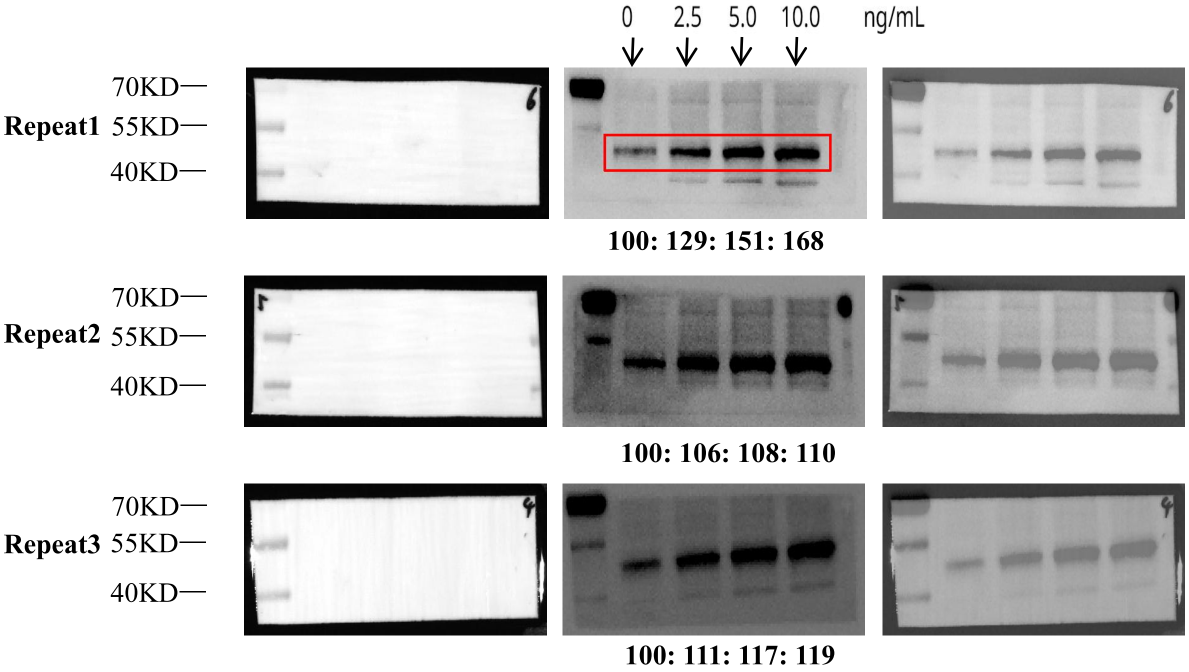

11.1.4 t-ERK1/2 (44/42KD):

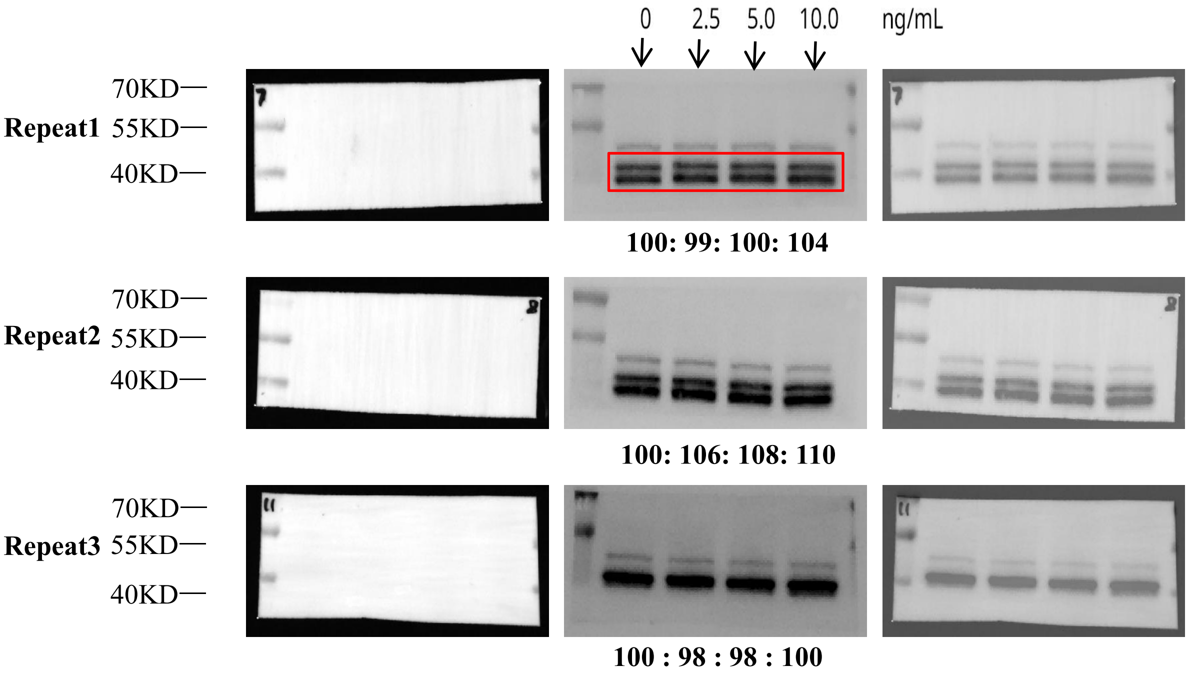

11.1.5 β-actin (42KD):

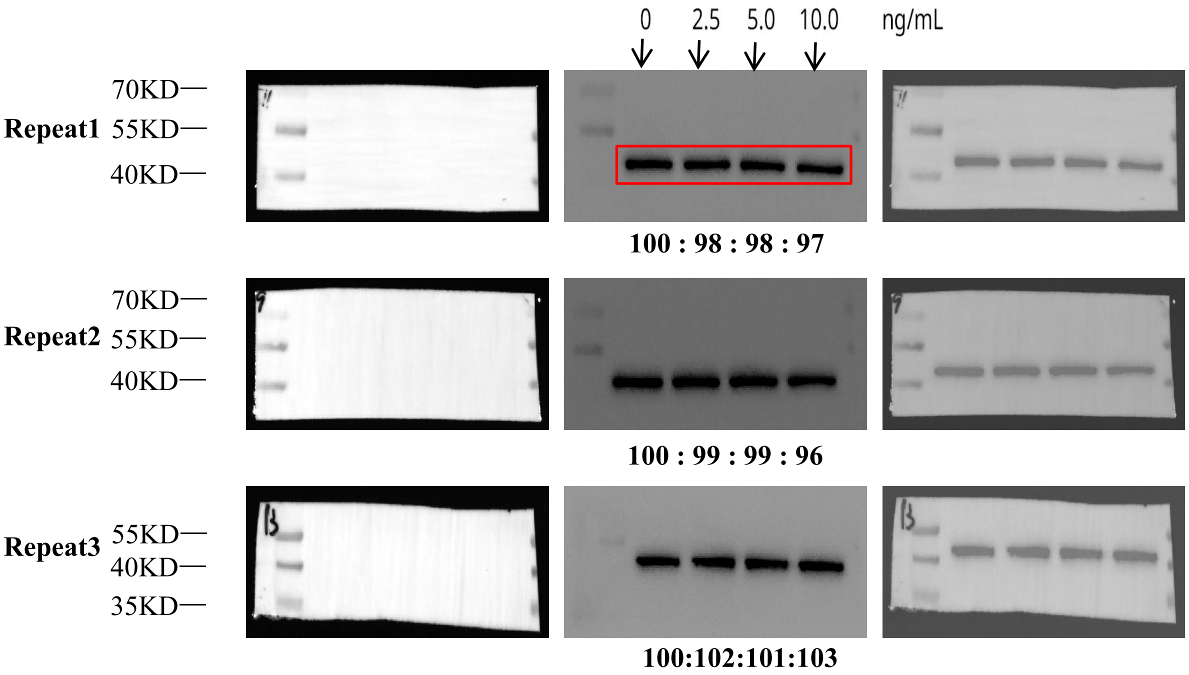

12 Supplementary Figure 4B and 4E:

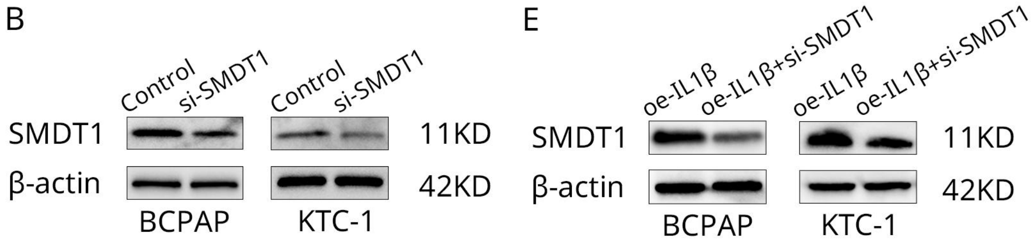

12.1 BCPAP

12.1.1 SMDT1 (11KD):

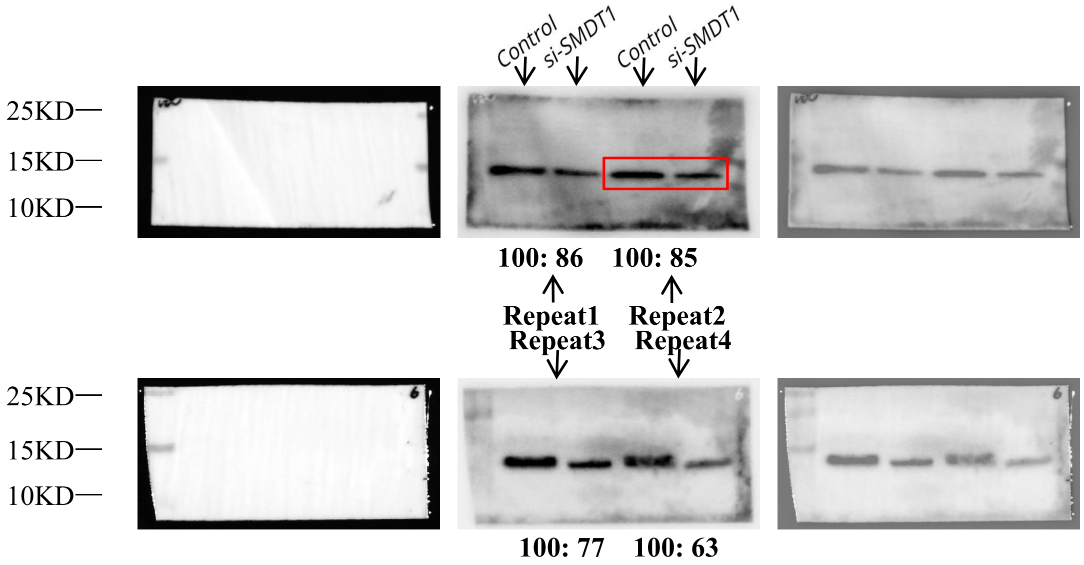

12.1.2 β-actin (42KD):

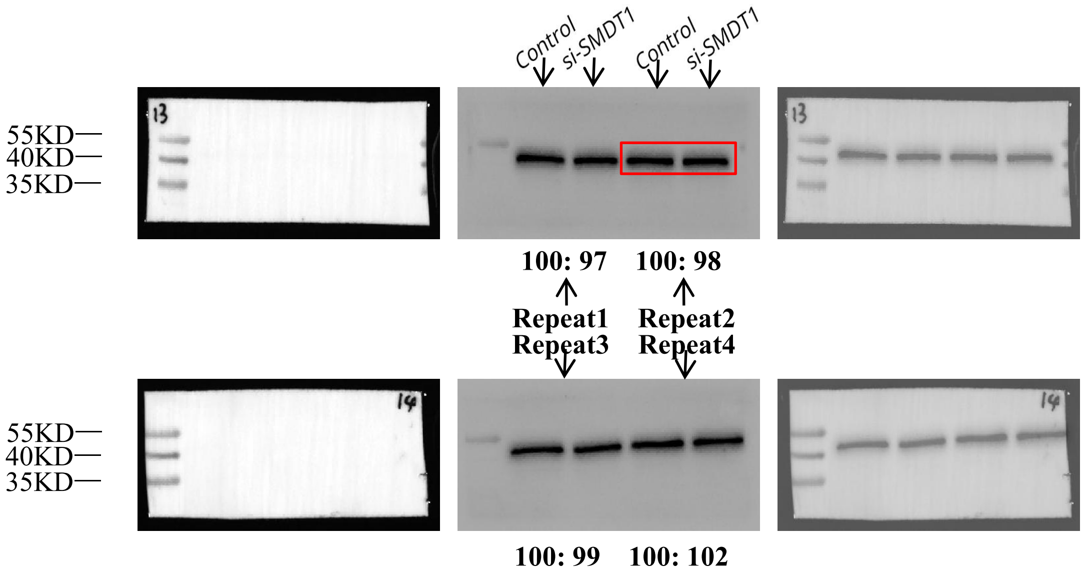

12.1.3 SMDT1 (11KD):

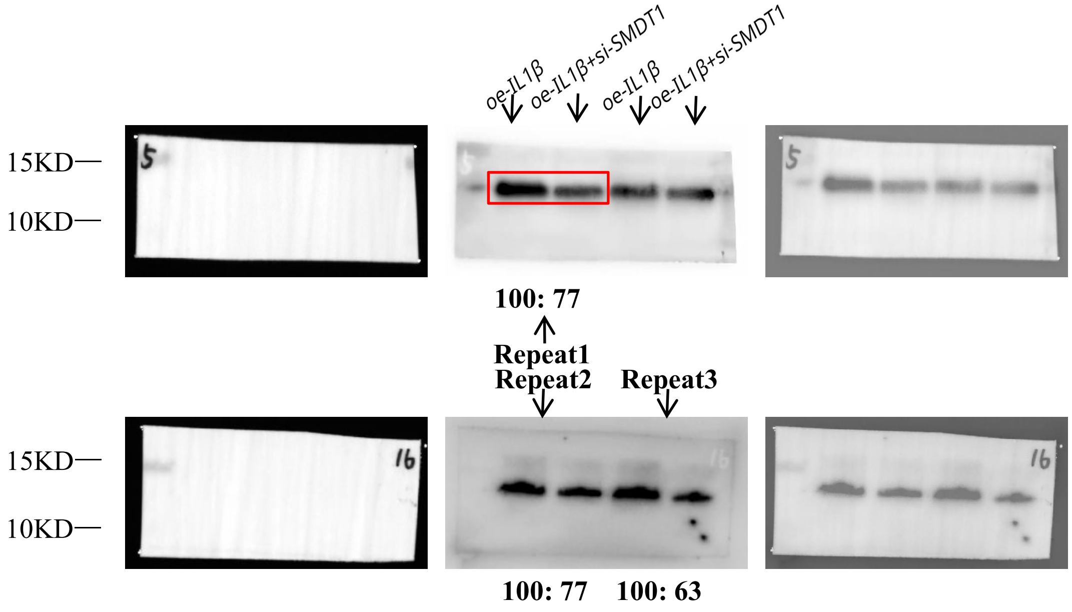

12.1.4 β-actin (42KD):

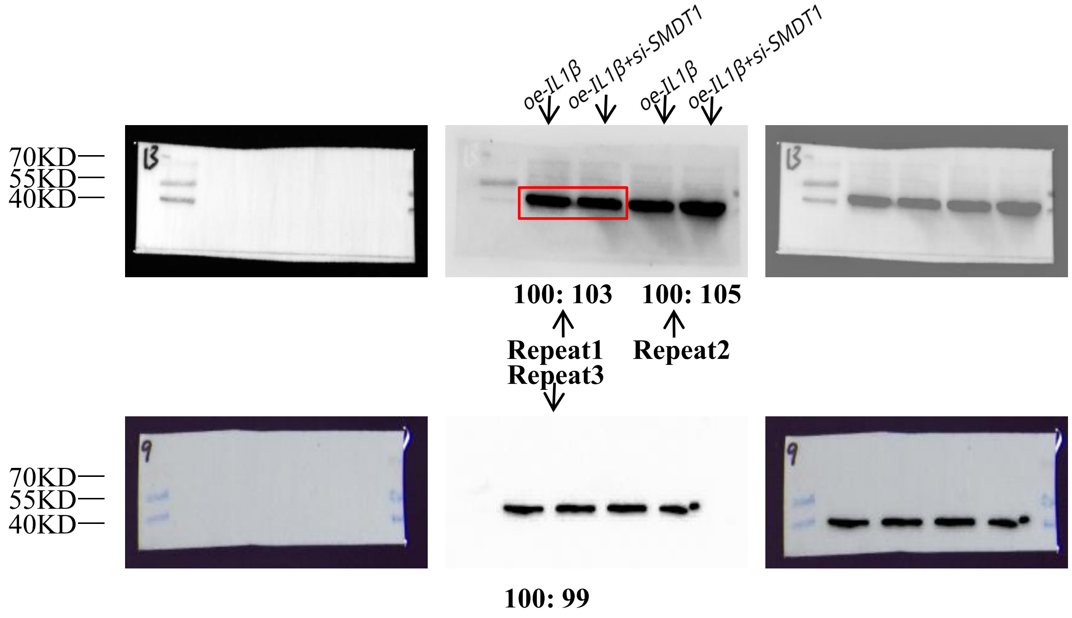

12.2 KTC-1

12.2.1 SMDT1 (11KD):

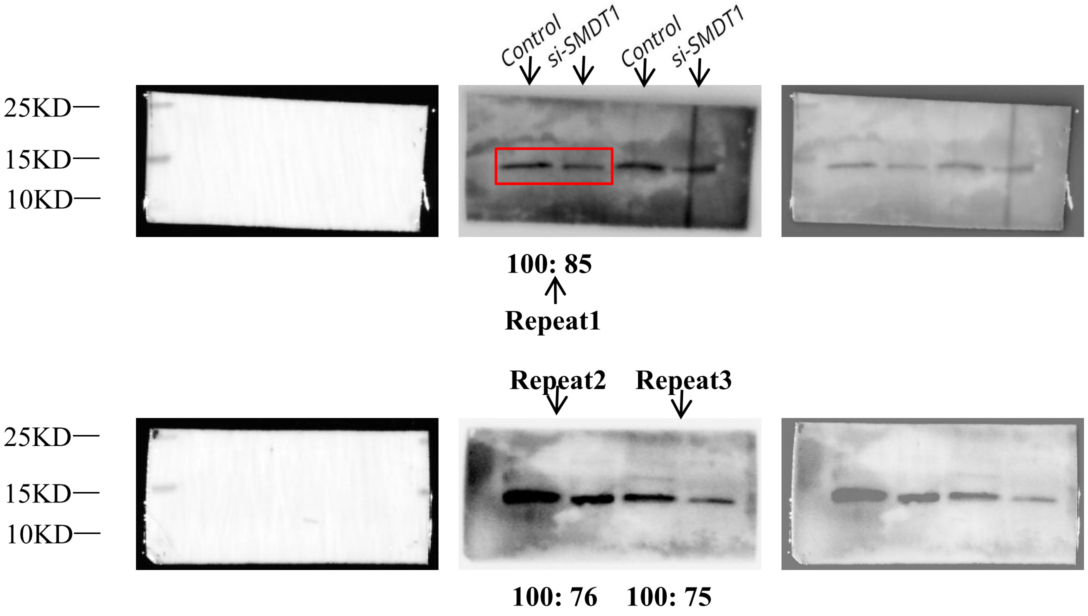

12.2.2 β-actin (42KD):

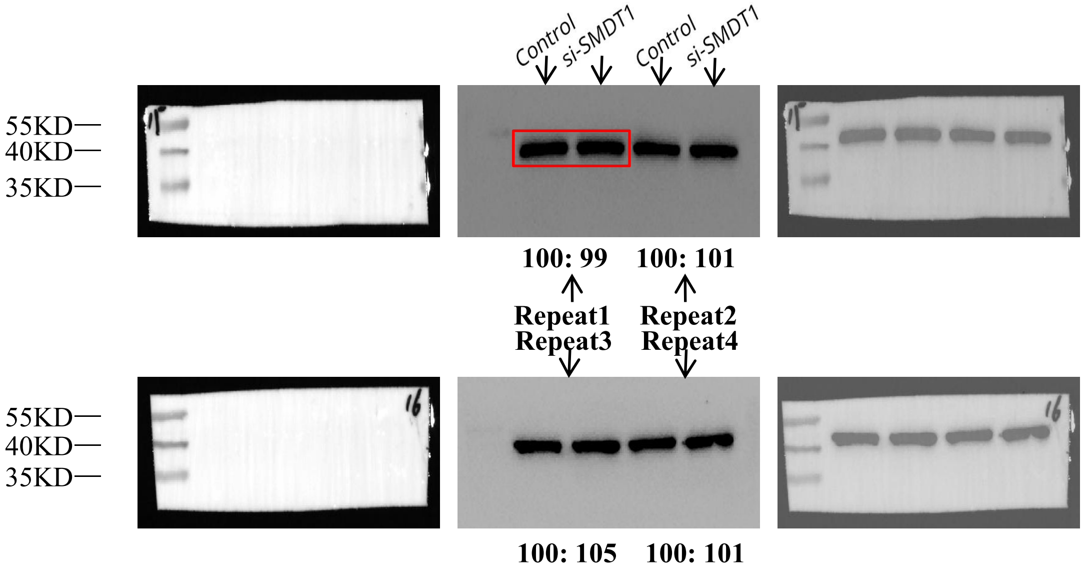

12.2.3 SMDT1 (11KD):

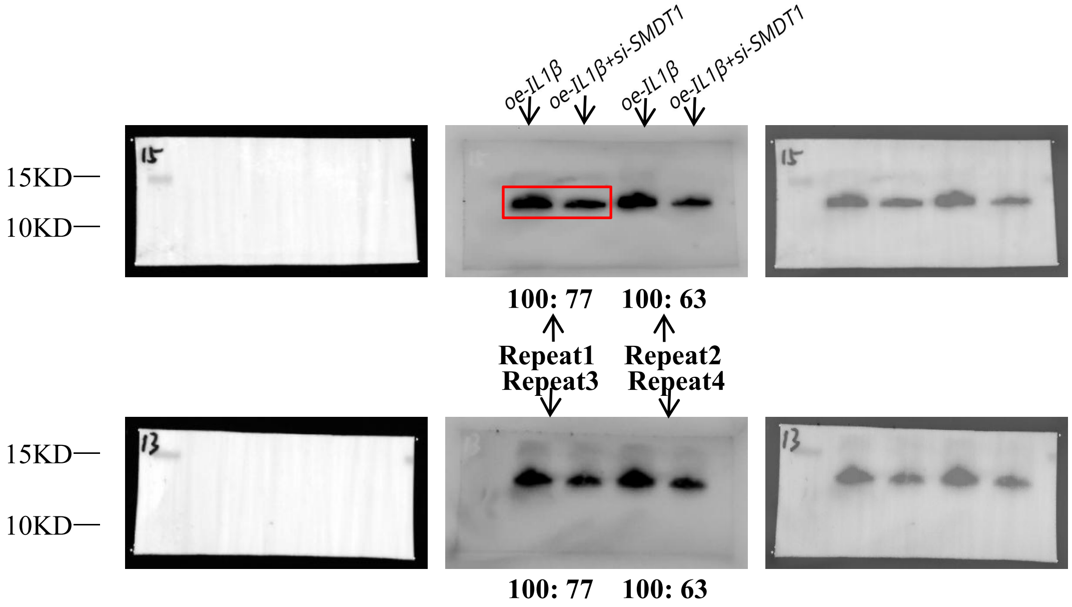

12.2.4 β-actin (42KD):

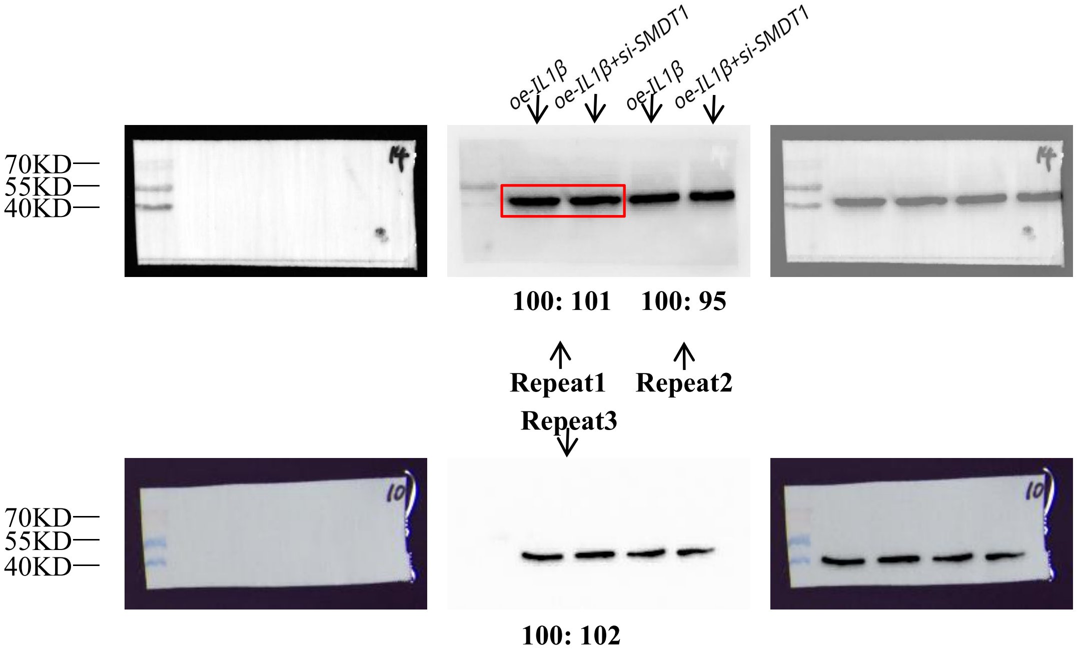

Supplement: Supplementary file 1 [file cancers-18-02918-s001.zip › WB original images-IL1β.pdf]
